# Supplementary material for: Characterization of Worldwide Olive Germplasm Banks of Marrakech (Morocco) and Córdoba (Spain): Towards management and use of olive germplasm in breeding programs
Source: PLoS One. 2019 Oct 17;14(10):e0223716. doi: 10.1371/journal.pone.0223716 (PMC6797134; doi:10.1371/journal.pone.0223716)
Supplement: S2 Table — Each allele size for each locus and each accession in two different genotyping conditions were superimposed. (DOCX) [file pone.0223716.s002.docx]

**S2 Table.** List of the 47 accessions from WOGBC used for alignment of alleles from both collections with 20 SSR markers. Each allele size for each locus and each accession in two different genotyping conditions were superimposed.

| **Code accessions** |  | **ssrOeUA-DCA1** | **ssrOeUA-DCA3** | **ssrOeUA-DCA4** | **ssrOeUA-DCA5** | **ssrOeUA-DCA8** | **ssrOeUA-DCA9** | **ssrOeUA-DCA10** | **ssrOeUA-DCA11** | **ssrOeUA-DCA15** | **ssrOeUA-DCA16** | **ssrOeUA-DCA18** | **EMO90** | **GAPU59** | **GAPU71A** | **GAPU71B** | **GAPU101** | **GAPU103A** | **UDO99-011** | **UDO99-017** | **UDO99-043** |
| --- | --- | --- | --- | --- | --- | --- | --- | --- | --- | --- | --- | --- | --- | --- | --- | --- | --- | --- | --- | --- | --- |
| COR000723 | New genotyping | 203/274 | 234/240 | 128/130 | 193/201 | 133/139 | 179/203 | 175/175 | 129/177 | 242/242 | 147/154 | 174/174 | 182/189 | 206/210 | 211/221 | 119/138 | 181/195 | 159/172 | 108/118 | 152/160 | 174/214 |
|  | Trujillo et al. (2014) | 204/274 | 234/241 | 129/130 | 195/203 | 134/139 | 180/204 | 176/176 | 130/178 | 243/243 | 148/154 | 174/174 | 183/189 | 206/210 | 212/221 | 121/141 | 183/197 | 159/171 | 114/125 | 160/160 | 175/214 |
| COR001026 | New genotyping | 203/203 | 231/246 | 116/128 | 201/205 | 133/139 | 173/191 | 151/151 | 177/181 | 242/242 | 123/123 | 172/172 | 182/208 | 206/210 | 211/211 | 116/122 | 195/201 | 147/172 | 108/112 | 160/160 | 170/212 |
|  | Trujillo et al. (2014) | 204/204 | 231/247 | 116/129 | 203/207 | 134/139 | 174/192 | 152/152 | 178/182 | 243/243 | 124/124 | 172/172 | 183/208 | 206/210 | 212/212 | 118/124 | 197/203 | 147/171 | 114/119 | 160/160 | 170/212 |
| COR001029 | New genotyping | 203/203 | 229/246 | 130/157 | 201/205 | 133/133 | 195/201 | 153/153 | 145/181 | 242/242 | 155/210 | 166/188 | 182/184 | 216/216 | 207/211 | 116/119 | 195/195 | 137/174 | 108/112 | 152/160 | 208/208 |
|  | Trujillo et al. (2014) | 204/204 | 229/247 | 130/157 | 203/207 | 134/133 | 196/202 | 154/154 | 146/182 | 243/243 | 154/211 | 166/189 | 183/185 | 216/216 | 207/212 | 118/121 | 197/197 | 137/174 | 114/119 | 152/160 | 169/208 |
| COR000653 | New genotyping | 203/211 | 242/242 | 130/164 | 189/201 | 137/164 | 183/191 | 145/145 | 125/145 | 242/242 | 121/123 | 162/166 | 180/180 | 220/220 | 207/209 | 119/138 | 187/189 | 147/157 | 118/118 | 152/152 | 172/214 |
|  | Trujillo et al. (2014) | 204/212 | 243/243 | 130/163 | 191/203 | 137/163 | 184/192 | 146/146 | 126/146 | 243/243 | 122/124 | 162/166 | 181/181 | 220/220 | 207/210 | 121/141 | 189/191 | 147/157 | 125/125 | 152/152 | 214/214 |
| COR000077 | New genotyping | 211/213 | 236/236 | 130/148 | 197/201 | 133/152 | 169/181 | 161/161 | 139/145 | 242/252 | 147/160 | 162/174 | 184/184 | 210/220 | 207/211 | 122/138 | 181/195 | 157/172 | 98/118 | 154/154 | 174/185 |
|  | Trujillo et al. (2014) | 212/214 | 237/237 | 130/148 | 199/203 | 134/151 | 170/182 | 162/162 | 140/146 | 243/254 | 148/161 | 162/174 | 185/185 | 210/220 | 207/212 | 124/141 | 183/197 | 157/171 | 103/125 | 154/154 | 175/185 |
| COR000118 | New genotyping | 211/213 | 236/250 | 161/188 | 197/201 | 123/135 | 169/173 | 155/155 | 135/159 | 252/252 | 144/147 | 168/170 | 182/182 | 210/220 | 211/229 | 116/122 | 187/197 | 165/184 | 110/124 | 168/168 | 172/179 |
|  | Trujillo et al. (2014) | 212/214 | 237/251 | 162/188 | 199/203 | 123/135 | 170/174 | 156/156 | 136/160 | 254/254 | 144/148 | 168/170 | 183/183 | 210/220 | 212/230 | 118/124 | 189/199 | 165/184 | 116/131 | 168/168 | 172/179 |
| COR000090 | New genotyping | 203/203 | 238/240 | 130/161 | 197/201 | 135/137 | 160/183 | 151/155 | 125/139 | 242/242 | 147/162 | 166/172 | 189/193 | 206/206 | 211/211 | 119/125 | 189/203 | 147/157 | 120/129 | 152/156 | 208/212 |
|  | Trujillo et al. (2014) | 204/204 | 239/241 | 130/161 | 199/203 | 135/137 | 160/184 | 152/156 | 126/140 | 243/243 | 148/163 | 166/172 | 189/193 | 206/206 | 212/212 | 121/127 | 191/205 | 147/157 | 127/138 | 152/157 | 208/212 |
| COR001081 | New genotyping | 203/203 | 236/242 | 128/161 | 201/201 | 129/133 | 169/183 | 193/193 | 129/145 |  | 125/171 | 158/172 | 189/189 | 206/220 | 211/211 | 119/119 | 189/189 | 147/172 | 112/112 | 152/152 | 174/174 |
|  | Trujillo et al. (2014) | 204/204 | 237/243 | 129/161 | 203/203 | 129/134 | 170/184 | 194/238 | 130/146 | 263/263 | 126/173 | 158/172 | 183/189 | 206/206 | 212/212 | 121/121 | 191/191 | 147/171 | 119/119 | 152/152 | 175/175 |
| COR000079 | New genotyping | 203/268 | 236/240 | 128/161 | 193/201 | 135/139 | 179/191 | 175/175 | 129/169 | 242/242 | 147/170 | 172/176 | 182/189 | 210/210 |  | 119/138 | 195/215 | 133/159 | 108/124 | 154/160 | 174/198 |
|  | Trujillo et al. (2014) | 204/268 | 237/241 | 129/161 | 195/203 | 139/139 | 180/192 | 176/176 | 130/170 | 243/243 | 148/171 | 172/176 | 183/189 | 210/210 | 207/221 | 121/141 | 197/217 | 133/159 | 114/131 | 154/160 | 175/198 |
| COR000659 | New genotyping | 211/215 | 236/246 | 161/186 | 189/201 | 133/135 | 169/191 | 153/153 | 159/177 | 242/242 | 123/147 | 176/176 | 182/189 | 210/210 | 207/207 | 125/138 | 195/197 | 172/182 | 120/124 | 152/156 | 172/214 |
|  | Trujillo et al. (2014) | 212/212 | 237/247 | 161/186 | 191/203 | 135/135 | 170/192 | 154/154 | 160/178 | 243/243 | 124/148 | 176/176 | 183/189 | 210/210 | 207/207 | 127/141 | 197/199 | 171/182 | 127/131 | 152/157 | 172/214 |
| COR000993 | New genotyping | 203/203 | 242/246 | 130/160 | 201/205 | 129/133 | 160/205 | 155/217 | 145/155 | 242/242 | 121/218 | 166/172 | 180/182 | 210/216 | 211/211 | 119/138 | 187/195 | 137/174 | 108/112 | 152/160 | 208/216 |
|  | Trujillo et al. (2014) | 204/204 | 243/247 | 130/159 | 203/207 |  | 160/206 |  | 146/156 | 243/243 | 122/218 | 166/172 | 181/183 | 210/216 | 212/212 | 121/141 | 189/197 | 137/174 | 114/119 |  | 208/216 |
| COR001142 | New genotyping | 203/203 | 229/250 | 161/161 | 201/201 | 123/123 | 183/205 | 182/182 | 141/145 | 242/242 | 121/121 | 174/174 | 180/182 | 206/216 | 209/211 | 119/133 | 189/189 | 141/210 | 112/120 | 152/160 | 174/176 |
|  | Trujillo et al. (2014) | 204/204 | 229/251 | 161/161 | 203/203 | 123/123 | 184/206 | 184/184 | 142/146 | 243/243 | 122/122 | 174/174 | 181/183 | 206/216 | 210/212 | 121/135 | 191/191 | 141/208 | 119/127 | 152/160 | 175/177 |
| COR000695 | New genotyping | 203/203 | 246/250 | 128/160 | 201/203 | 135/135 | 160/179 | 155/215 | 175/181 | 242/242 | 152/181 | 166/172 | 182/182 | 210/210 | 205/211 | 116/119 | 197/215 | 133/178 | 112/120 | 152/152 | 172/174 |
|  | Trujillo et al. (2014) | 204/204 | 247/251 | 129/159 | 203/205 | 135/135 | 160/180 | 216/216 | 176/182 | 243/243 | 152/183 | 166/172 | 183/183 | 210/210 | 206/212 | 118/121 | 199/217 | 133/178 | 119/127 | 152/152 | 172/175 |
| COR000128 | New genotyping | 203/229 | 236/250 | 136/161 | 201/209 | 123/135 | 189/191 | 155/155 | 125/177 | 242/242 | 162/177 | 168/176 | 182/182 | 210/222 | 211/225 | 116/122 | 181/197 | 157/184 | 112/124 | 152/152 | 170/170 |
|  | Trujillo et al. (2014) | 204/229 | 237/251 | 136/161 | 203/211 | 123/135 | 190/192 | 156/156 | 126/178 | 243/243 | 163/179 | 168/176 | 183/183 | 210/222 | 212/225 | 118/124 | 183/199 | 157/184 | 119/131 | 152/152 | 170/172 |
| COR000680 | New genotyping | 203/239 | 236/236 | 130/152 | 189/189 | 137/145 | 179/191 | 155/155 | 139/145 | 259/261 | 144/147 | 168/170 | 182/189 | 206/210 | 211/211 | 119/125 | 189/203 | 147/157 | 118/118 | 152/152 | 170/206 |
|  | Trujillo et al. (2014) | 204/240 | 237/237 | 130/152 | 191/191 | 137/145 | 180/192 | 156/156 | 140/146 | 261/263 | 144/148 | 168/170 | 183/189 | 206/210 | 212/212 | 121/127 | 191/205 | 147/157 | 125/125 | 152/152 | 170/206 |
| COR001054 | New genotyping | 203/203 | 246/246 | 159/159 | 201/201 | 125/133 | 169/185 | 155/229 | 145/159 | 242/242 | 123/179 | 172/178 | 180/180 | 210/210 | 207/211 | 122/138 | 193/195 | 155/172 | 108/120 | 160/160 | 172/214 |
|  | Trujillo et al. (2014) | 204/204 | 247/247 | 159/159 | 203/203 | 125/134 | 170/186 | 230/230 | 146/160 | 243/243 | 124/181 | 172/178 | 181/181 | 210/210 | 207/212 | 124/141 | 195/197 | 155/171 | 114/127 |  | 172/214 |
| COR001002 | New genotyping | 203/203 | 242/248 | 159/159 | 201/201 | 133/150 | 173/201 | 197/197 | 145/145 | 242/242 | 121/121 | 182/184 | 184/195 | 212/218 | 211/211 | 119/122 | 189/201 | 133/143 | 120/120 | 152/160 | 170/170 |
|  | Trujillo et al. (2014) | 204/204 | 243/249 | 159/159 | 203/203 | 134/149 | 174/202 |  | 146/146 | 243/243 | 122/122 | 183/185 |  | 212/218 | 212/212 | 121/124 | 191/203 | 133/143 | 127/127 | 152/160 | 170/170 |
| COR000667 | New genotyping | 211/213 | 229/236 | 130/138 | 193/201 | 129/160 | 177/181 | 151/151 | 133/173 | 242/242 | 147/157 | 168/176 | 182/184 | 210/210 | 211/211 | 116/138 | 181/189 | 147/161 | 106/118 | 154/154 | 176/212 |
|  | Trujillo et al. (2014) | 212/214 | 229/237 | 130/138 | 195/203 | 129/159 | 178/182 | 152/152 | 134/174 | 243/243 | 148/159 | 168/176 | 183/185 | 210/210 | 212/212 | 118/141 | 183/191 | 147/161 | 112/125 | 154/154 | 177/212 |
| COR000105 | New genotyping | 203/203 | 229/250 | 154/154 | 201/201 | 133/135 | 163/191 | 173/173 | 133/159 | 259/261 | 121/123 | 180/182 | 182/193 | 206/210 | 211/211 | 116/122 | 189/197 | 159/184 | 108/112 | 152/160 | 208/214 |
|  | Trujillo et al. (2014) | 204/204 | 229/251 | 154/154 | 203/203 | 135/135 | 164/192 | 174/174 | 134/160 | 262/263 | 122/124 | 180/183 | 183/193 | 206/210 | 212/212 | 118/124 | 191/199 | 159/184 | 114/119 | 152/160 | 208/214 |
| COR001171 | New genotyping | 203/203 | 242/244 | 138/159 | 193/201 | 129/152 | 169/191 | 171/171 | 133/177 | 242/252 | 152/173 | 166/172 | 189/189 | 220/220 | 211/225 | 119/119 | 189/191 | 147/155 | 112/118 | 152/152 | 172/174 |
|  | Trujillo et al. (2014) | 204/204 | 243/245 | 138/159 | 195/203 | 129/151 | 170/192 | 172/172 | 134/178 | 243/254 | 152/175 | 166/172 | 183/189 | 206/220 | 212/225 | 121/121 | 191/193 | 133/174 | 119/125 | 152/152 | 172/175 |
| COR001151 | New genotyping | 203/211 | 229/240 | 128/130 | 189/203 | 129/133 | 181/183 | 151/167 | 129/139 | 242/242 | 121/147 | 172/174 | 184/193 | 216/220 |  | 119/138 | 189/195 | 147/172 | 108/124 | 154/160 | 174/214 |
|  | Trujillo et al. (2014) | 204/212 | 229/241 | 129/130 | 191/205 | 129/134 | 182/184 | 152/168 | 130/140 | 243/254 | 122/147 | 172/174 | 185/193 | 216/220 | 212/242 | 121/141 | 191/197 | 147/171 | 114/131 | 154/160 | 175/214 |
| COR000688 | New genotyping | 203/205 | 234/250 | 159/159 | 195/195 | 135/143 | 173/173 | 151/151 | 179/181 | 242/242 | 121/228 | 178/188 | 180/187 | 204/210 | 209/211 | 116/144 | 187/203 | 137/137 | 108/112 | 152/160 | 174/204 |
|  | Trujillo et al. (2014) | 204/206 | 234/251 | 159/159 | 197/197 | 135/143 | 174/174 | 152/152 | 180/180 | 243/243 | 122/228 | 178/189 | 181/187 | 204/210 | 210/212 | 118/147 | 189/205 | 137/137 | 114/119 | 152/160 | 175/204 |
| COR001472 | New genotyping | 203/257 | 240/250 | 128/130 | 193/201 | 133/135 | 160/203 | 153/175 | 129/177 | 252/252 | 147/171 | 172/172 | 182/189 | 206/210 | 211/221 | 119/138 | 195/197 | 172/184 | 108/127 | 154/160 | 210/214 |
|  | Trujillo et al. (2014) | 204/258 | 241/251 | 129/130 | 195/203 | 135/135 | 160/204 | 154/176 | 130/178 | 254/254 | 148/173 | 172/172 | 183/189 | 206/210 | 212/221 | 121/141 | 197/199 | 171/184 | 114/134 | 154/160 | 210/214 |
| COR000703 | New genotyping | 203/211 | 236/236 | 130/166 | 201/203 | 133/133 | 160/203 | 175/175 | 159/177 | 261/261 | 142/171 | 172/193 | 182/184 | 210/220 | 211/211 | 119/122 | 187/195 | 172/176 | 112/124 | 152/154 | 179/179 |
|  | Trujillo et al. (2014) | 204/212 | 237/237 | 130/165 | 203/205 | 134/133 | 160/204 | 176/176 | 160/178 | 263/263 | 143/173 | 172/193 | 183/185 | 210/220 | 212/212 | 121/124 | 189/197 | 171/176 | 119/131 | 152/154 | 175/179 |
| COR001118 | New genotyping | 203/203 | 231/250 | 128/130 | 201/201 | 125/168 | 185/199 | 186/186 | 139/159 | 244/242 | 123/171 | 184/190 | 180/191 | 210/210 | 207/209 | 122/122 | 193/193 | 147/180 | 114/116 | 152/152 | 172/172 |
|  | Trujillo et al. (2014) | 204/204 | 232/251 | 129/130 | 203/203 | 125/168 | 186/200 | 188/188 | 140/160 | 245/245 | 124/173 | 183/191 | 181/191 | 210/210 | 207/210 | 124/124 | 195/195 | 147/180 | 121/123 | 152/152 | 172/172 |
| COR000226 | New genotyping | 211/231 | 240/242 | 192/192 | 193/201 | 125/162 | 183/199 | 139/139 | 125/149 | 261/265 | 121/135 | 162/176 | 182/182 | 210/220 | 207/211 | 122/125 | 181/189 | 172/172 | 108/118 | 160/160 | 174/216 |
|  | Trujillo et al. (2014) | 212/232 | 241/243 | 192/192 | 195/203 | 125/161 | 184/200 | 140/140 | 126/150 | 243/263 | 122/136 | 162/176 | 183/183 | 210/220 | 207/212 | 124/127 | 183/191 | 171/171 | 114/125 | 160/160 | 175/216 |
| COR001125 | New genotyping | 203/211 | 246/246 | 159/159 | 201/201 | 125/129 | 169/191 | 151/153 | 159/159 | 261/261 | 121/175 | 170/172 | 182/197 | 206/210 | 207/211 | 116/119 | 187/193 | 147/153 | 112/120 | 160/168 | 172/210 |
|  | Trujillo et al. (2014) | 204/212 | 247/247 | 159/159 | 203/203 | 125/129 | 170/192 | 152/154 | 160/160 | 263/263 | 122/177 | 170/172 | 183/198 | 206/210 | 207/212 | 118/121 | 189/195 | 147/153 | 103/119 | 160/168 | 172/210 |
| COR000809 | New genotyping | 211/211 | 240/242 | 182/196 | 193/201 | 137/150 | 183/209 | 145/145 | 145/145 | 242/242 | 147/173 | 168/182 | 184/184 | 210/220 | 211/211 | 122/125 | 181/203 | 147/159 | 98/112 | 152/154 | 172/174 |
|  | Trujillo et al. (2014) | 212/212 | 241/243 | 182/196 | 195/203 |  | 184/210 |  | 146/146 | 243/243 | 148/175 | 168/183 |  | 210/220 | 212/212 | 124/127 | 183/205 | 147/159 | 103/119 |  | 172/175 |
| COR001143 | New genotyping | 203/203 | 231/250 | 159/161 | 201/201 | 123/135 | 185/185 | 179/179 | 141/167 | 242/242 | 121/175 | 166/174 | 182/182 | 216/216 | 209/209 | 122/133 | 191/191 | 133/206 | 112/120 | 152/160 | 172/174 |
|  | Trujillo et al. (2014) | 204/204 | 232/251 | 159/161 | 203/203 | 123/135 | 186/186 | 180/180 | 142/168 | 243/243 | 122/177 | 166/174 | 183/183 | 216/216 | 210/210 | 124/135 | 193/193 | 133/204 | 119/127 | 152/160 | 172/175 |
| COR000345 | New genotyping | 203/203 | 229/240 | 182/190 | 189/201 | 137/145 | 179/203 | 155/155 | 139/145 | 259/261 | 121/144 | 158/166 | 182/184 | 206/206 | 211/211 | 125/138 | 189/203 | 147/157 | 108/118 | 160/160 | 174/185 |
|  | Trujillo et al. (2014) | 204/204 | 229/241 | 182/190 | 191/203 | 137/145 | 180/204 | 156/156 | 140/146 | 261/263 | 122/144 | 158/166 | 183/185 | 206/206 | 212/212 | 127/141 | 191/205 | 147/157 | 114/125 | 160/160 | 185/185 |
| COR001001 | New genotyping | 203/203 | 229/242 | 145/159 | 201/201 | 133/152 | 179/201 | 182/182 | 145/151 | 242/244 | 121/224 | 166/174 | 180/182 | 206/210 | 211/211 | 122/138 | 187/195 | 137/141 | 112/120 | 152/152 | 216/219 |
|  | Trujillo et al. (2014) | 204/204 | 229/243 | 145/159 | 203/203 | 134/151 | 180/202 | 182/182 | 146/152 | 243/245 | 122/224 | 166/174 | 181/183 | 206/210 | 212/212 | 124/141 | 189/197 | 137/141 | 119/127 | 152/152 | 216/218 |
| COR000699 | New genotyping | 203/211 | 229/236 | 128/138 | 201/207 | 129/133 | 179/203 | 206/206 | 139/139 | 242/252 | 121/164 | 170/172 | 184/193 | 216/218 | 207/211 | 122/138 | 189/195 | 147/176 | 108/122 | 152/160 | 176/176 |
|  | Trujillo et al. (2014) | 204/212 | 229/237 | 129/138 | 203/209 | 129/134 | 180/204 | 208/208 | 140/140 | 243/254 | 122/165 | 170/172 | 185/193 | 216/218 | 207/212 | 124/141 | 191/197 | 147/176 | 114/129 | 152/160 | 175/177 |
| COR000152 | New genotyping | 203/211 | 236/242 |  | 203/203 | 125/133 | 191/191 | 157/157 | 145/145 | 242/242 | 123/123 | 166/168 | 182/197 | 212/216 | 207/207 | 116/122 | 195/203 | 155/172 | 110/120 | 152/154 | 216/216 |
|  | Trujillo et al. (2014) | 204/212 | 237/243 |  | 205/205 | 125/134 | 192/192 | 158/158 | 146/146 | 243/243 | 124/124 | 166/168 | 183/198 | 212/216 | 207/207 | 118/124 | 197/205 | 155/171 | 116/127 | 154/154 | 169/216 |
| COR000108 | New genotyping | 203/211 | 229/240 | 138/186 | 201/209 | 137/152 | 163/169 | 155/155 | 129/177 | 261/265 | 147/208 | 172/174 | 182/189 | 218/218 | 207/211 | 122/138 | 181/203 | 147/157 | 110/112 | 168/168 | 174/202 |
|  | Trujillo et al. (2014) | 204/212 | 229/241 | 138/186 | 203/211 | 137/151 | 164/170 | 156/156 | 130/178 | 263/267 | 148/209 | 172/174 | 183/189 | 206/218 | 207/212 | 124/141 | 183/205 | 147/157 | 116/119 | 168/168 | 175/202 |
| COR000669 | New genotyping | 211/213 | 229/229 | 142/157 | 195/201 | 129/156 | 183/191 | 193/193 | 125/139 | 242/242 | 121/147 | 166/176 | 180/184 | 206/226 | 207/211 | 122/122 | 181/187 | 172/172 | 120/122 | 152/160 | 174/174 |
|  | Trujillo et al. (2014) | 212/214 | 229/229 | 142/157 | 197/203 | 129/155 | 184/192 | 194/194 | 126/140 | 243/243 | 122/148 | 166/176 | 181/185 | 206/227 | 207/212 | 124/124 | 183/189 | 171/171 | 127/129 | 152/160 | 175/175 |
| COR000846 | New genotyping | 205/237 | 234/248 | 153/198 | 199/203 | 135/137 | 169/195 | 215/261 | 161/177 | 242/242 | 123/171 | 172/188 | 187/195 | 206/210 | 211/211 | 116/122 | 203/215 | 141/172 | 108/110 | 152/160 | 170/172 |
|  | Trujillo et al. (2014) | 206/206 | 234/249 | 152/198 | 201/205 | 135/135 | 170/196 | 216/260 | 162/178 | 243/243 | 124/173 | 172/189 | 187/196 | 206/210 | 212/212 | 118/124 | 205/217 | 141/171 | 114/116 | 152/160 | 170/172 |
| COR000224 | New genotyping | 211/211 | 240/242 | 184/194 | 193/201 | 137/150 | 183/205 | 145/145 | 145/145 | 242/242 | 147/173 | 168/180 | 184/184 | 210/220 | 211/211 | 122/125 | 181/203 | 147/159 | 98/112 | 152/154 | 172/174 |
|  | Trujillo et al. (2014) | 212/212 | 241/243 | 184/194 | 195/203 | 137/149 | 184/206 | 146/146 | 146/146 | 243/243 | 148/175 | 168/180 | 185/185 | 210/220 | 212/212 | 124/127 | 183/205 | 147/159 | 103/119 | 152/154 | 172/175 |
| COR000562 | New genotyping | 211/213 | 227/236 | 130/130 | 201/205 | 135/145 | 169/183 | 153/153 | 139/159 | 242/242 | 121/170 | 164/172 | 182/182 | 210/216 | 211/211 | 116/119 | 181/197 | 157/184 | 108/120 | 156/160 | 172/172 |
|  | Trujillo et al. (2014) | 212/214 | 227/237 | 130/130 | 203/207 | 135/145 | 170/184 | 154/154 | 140/160 | 243/243 | 122/171 | 164/172 | 183/183 | 210/216 | 212/212 | 118/121 | 183/199 | 157/184 | 114/127 | 157/160 | 172/172 |
| COR000070 | New genotyping | 203/264 | 229/250 | 159/159 | 197/201 | 135/137 | 191/191 | 193/217 | 129/177 | 242/242 | 144/171 | 166/176 | 182/189 | 210/210 | 211/211 | 119/138 | 197/203 | 149/188 | 108/120 | 156/160 | 208/216 |
|  | Trujillo et al. (2014) | 204/265 | 229/251 | 159/159 | 199/203 | 135/137 | 192/192 | 194/218 | 130/178 | 243/243 | 144/173 | 166/176 | 183/189 | 210/210 | 212/212 | 121/141 | 199/205 | 149/188 | 114/127 | 157/160 | 208/216 |
| COR000969 | New genotyping | 203/215 | 231/236 | 138/186 | 193/201 | 129/133 | 191/203 | 177/177 | 129/139 | 242/242 | 121/157 | 170/172 | 180/193 | 216/220 | 207/211 | 119/119 | 189/189 | 147/172 | 110/124 | 168/168 | 174/198 |
|  | Trujillo et al. (2014) | 204/204 | 231/237 | 138/186 | 195/203 | 129/134 | 192/204 | 178/178 | 130/140 | 243/243 | 122/157 | 170/172 | 181/193 | 216/220 | 207/212 | 121/121 | 191/191 | 147/171 | 116/131 | 168/168 | 175/198 |
| COR000705 | New genotyping | 203/211 | 236/246 | 130/186 | 189/201 | 129/135 | 191/191 | 193/217 | 139/159 | 252/252 | 123/168 | 168/172 | 180/182 | 210/210 | 207/211 | 125/125 | 189/215 | 133/147 | 108/112 | 152/160 | 174/174 |
|  | Trujillo et al. (2014) | 204/212 | 237/247 | 130/186 | 191/203 | 129/135 | 192/192 | 194/218 | 140/160 | 254/254 | 124/169 | 168/172 | 181/183 | 210/210 | 207/212 | 127/127 | 191/217 | 133/147 | 114/119 | 152/160 | 175/175 |
| COR000119 | New genotyping | 203/211 | 236/250 | 130/130 | 201/201 | 135/137 | 191/203 | 153/153 | 139/159 | 242/252 | 152/171 | 168/176 | 182/184 | 210/220 | 211/211 | 116/125 | 195/215 | 133/147 | 120/127 | 152/154 | 172/212 |
|  | Trujillo et al. (2014) | 204/212 | 237/251 | 130/130 | 203/203 | 137/137 | 192/204 | 154/154 | 140/160 | 243/254 | 152/173 | 168/176 | 183/185 | 210/220 | 212/212 | 118/127 | 197/217 | 133/147 | 127/134 | 152/154 | 172/212 |
| COR000095 | New genotyping | 203/203 | 250/250 | 116/132 | 199/201 | 129/135 | 160/201 | 147/147 | 145/163 | 242/242 | 152/171 | 168/172 | 180/182 | 210/216 | 207/211 | 116/138 | 187/197 | 172/184 | 108/120 | 156/160 | 208/214 |
|  | Trujillo et al. (2014) | 204/204 | 251/251 | 116/132 | 201/203 | 129/135 | 160/202 | 148/148 | 146/164 | 243/243 | 152/173 | 168/172 | 181/183 | 210/216 | 207/212 | 118/141 | 189/199 | 171/184 | 114/127 | 157/160 | 208/214 |
| COR000394 | New genotyping | 203/211 | 242/246 | 130/130 | 201/201 | 135/139 | 160/203 | 153/153 | 159/171 | 242/242 | 123/152 | 168/176 | 182/182 | 210/210 | 207/211 | 116/125 | 189/215 | 184/184 | 110/120 | 156/168 | 208/216 |
|  | Trujillo et al. (2014) | 204/212 | 243/247 | 130/130 | 203/203 | 135/139 | 160/204 | 154/154 | 160/172 | 243/243 | 124/152 | 168/176 | 183/183 | 210/210 | 207/212 | 118/127 | 191/217 | 184/184 | 116/127 | 157/168 | 208/216 |
| COR000364 | New genotyping | 203/211 | 250/252 | 128/161 | 201/201 | 129/145 | 160/179 | 217/217 | 139/177 | 242/242 | 144/171 | 172/176 | 182/193 | 206/210 | 211/211 | 116/119 |  | 147/157 | 112/118 | 152/152 | 172/216 |
|  | Trujillo et al. (2014) | 204/212 | 251/253 | 129/161 | 203/203 | 129/145 | 160/180 | 218/218 | 140/178 | 243/243 | 144/173 | 172/176 | 183/193 | 206/210 | 212/212 | 118/121 |  | 147/157 | 119/125 | 152/152 | 172/216 |
| COR000051 | New genotyping | 211/213 | 246/250 |  | 201/205 | 137/139 | 181/191 | 153/153 | 139/165 | 252/252 | 123/123 | 166/176 | 182/184 | 206/220 | 211/211 | 125/138 | 189/195 | 147/147 | 108/110 | 160/168 | 202/216 |
|  | Trujillo et al. (2014) | 212/214 | 247/251 |  | 203/207 | 137/139 | 182/192 | 154/154 | 140/166 | 254/254 | 124/124 | 166/176 | 183/185 | 206/220 | 212/212 | 127/141 | 191/197 | 147/147 | 114/116 | 160/168 | 202/216 |
| COR000725 | New genotyping | 203/211 | 229/229 | 128/186 | 195/201 | 125/152 | 169/213 | 153/169 | 139/151 | 242/242 |  | 170/172 | 182/184 | 206/210 |  | 138/141 | 189/195 | 141/157 | 118/118 | 152/172 | 166/193 |
|  | Trujillo et al. (2014) | 204/212 | 229/229 | 129/186 | 197/203 | 125/151 | 170/214 | 154/170 | 140/152 | 243/243 | 144/148 | 170/172 | 183/185 | 206/210 | 210/212 | 141/144 | 191/197 | 141/157 | 125/125 | 152/173 | 166/194 |

**Note.** A total of 47 accessions from WOGBC, analyzed by Trujill et al. (2014), were genotyped using 20 SSR markers in the same laboratory conditions as for those issued from WOGBM. Allele’s sizes revealed were converted into allele’s sizes detected by Trujillo et al. (2014). We note that most allele’s size differences observed between both genotyping conditions were one or two bases. for instance at the locus DCA01, when aligning same accessions genotyped in the two laboratory conditions we observed that Trujillo et al. (2014) detected an allele’s size of 204 and 212 whereas in our condition we detected 203 and 211, respectively. Thus, 203 and 211 allele’s sizes observed in the WOGBM at the DCA01 locus were converted into 204 and 212, respectively.
