# Supplementary material for: Characterization of Worldwide Olive Germplasm Banks of Marrakech (Morocco) and Córdoba (Spain): Towards management and use of olive germplasm in breeding programs
Source: PLoS One. 2019 Oct 17;14(10):e0223716. doi: 10.1371/journal.pone.0223716 (PMC6797134; doi:10.1371/journal.pone.0223716)
Supplement: S3 Table — Shared genotypes and those specific to each collection are indicated in the WOGB column. (DOCX) [file pone.0223716.s003.docx]

**S3 Table.** Data for the 672 SSR profiles observed in both WOGBM and WOGBC collections. Shared genotypes and those specific to each collection are indicated in the WOGB column.

| **No.** ^1^ | **WOGB ^2^** | **Code SSR** | **ssrOeUA-DCA1** | **ssrOeUA-DCA3** | **ssrOeUA-DCA4** | **ssrOeUA-DCA5** | **ssrOeUA-DCA8** | **ssrOeUA-DCA9** | **ssrOeUA-DCA10** | **ssrOeUA-DCA11** | **ssrOeUA-DCA15** | **ssrOeUA-DCA16** | **ssrOeUA-DCA18** | **EMO90** | **GAPU59** | **GAPU71A** | **GAPU71B** | **GAPU101** | **GAPU103** | **UDO99-011** | **UDO99-017** | **UDO99-043** |
| --- | --- | --- | --- | --- | --- | --- | --- | --- | --- | --- | --- | --- | --- | --- | --- | --- | --- | --- | --- | --- | --- | --- |
| 1 | C | 1 | 212/214 | 227/237 | 130/130 | 203/207 | 135/145 | 170/184 | 154/154 | 140/160 | 243/243 | 122/171 | 164/172 | 183/183 | 210/216 | 212/212 | 118/121 | 183/199 | 157/184 | 114/127 | 157/160 | 172/172 |
| 2 | C | 2 | 212/214 | 227/237 | 130/130 | 203/207 | 135/145 | 170/184 | 154/154 | 140/160 | 243/243 | 122/171 | 164/172 | 183/183 | 210/216 | 212/212 | 118/121 | 183/199 | 157/190 | 114/127 | 157/160 | 172/172 |
| **3** | **C & M** | **3** | **204/212** | **227/237** | **130/150** | **203/203** | **123/134** | **178/204** | **162/162** | **126/178** | **243/243** | **144/148** | **174/176** | **183/185** | **220/220** | **207/212** | **121/141** | **183/197** | **157/171** | **103/125** | **154/154** | **175/216** |
| 4 | C | 4 | 204/212 | 227/237 | 130/150 | 203/203 | 123/134 | 178/204 | 162/162 | 126/178 | 243/243 | 144/148 | 174/176 | 183/185 | 220/220 | 207/212 | 121/141 | 183/197 | 157/171 | 103/125 | 154/154 | 175/218 |
| 5 | C | 5 | 204/214 | 227/243 | 130/162 | 203/207 | 123/135 | 182/192 | 154/154 | 160/178 | 263/263 | 144/173 | 166/172 | 183/183 | 210/210 | 207/212 | 118/121 | 191/199 | 157/184 | 116/119 | 152/154 | 208/212 |
| 6 | C | 6 | 204/212 | 227/253 | 130/161 | 203/203 | 129/135 | 160/184 | 154/154 | 160/178 | 243/243 | 122/173 | 172/176 | 183/193 | 206/210 | 207/212 | 118/121 | 191/199 | 147/182 | 125/127 | 157/157 | 172/218 |
| 7 | C | 7 | 204/204 | 229/229 | 129/161 | 203/203 | 129/135 | 160/192 | 218/218 | 146/182 | 243/243 | 122/173 | 172/180 | 181/183 | 206/210 | 207/212 | 118/121 | 189/199 | 147/186 | 114/127 | 157/160 | 170/218 |
| 8 | C | 8 | 204/204 | 229/229 | 129/161 | 203/203 | 129/135 | 160/192 | 218/218 | 146/182 | 243/243 | 122/173 | 172/180 | 181/183 | 206/210 | 207/212 | 118/121 | 189/199 | 147/184 | 114/127 | 157/160 | 170/218 |
| 9 | C | 9 | 204/204 | 229/229 | 129/161 | 203/203 | 129/135 | 160/192 | 218/218 | 146/182 | 243/243 | 122/173 | 176/180 | 181/183 | 206/210 | 207/212 | 118/121 | 189/199 | 147/184 | 114/127 | 157/160 | 170/218 |
| 10 | C | 10 | 204/204 | 229/229 | 129/129 | 203/203 | 135/151 | 160/214 | 218/218 | 140/146 | 243/243 | 148/148 | 166/172 | 183/183 | 206/210 | 207/212 | 118/144 | 191/199 | 141/188 | 119/125 | 160/173 | 194/216 |
| 11 | C | 11 | 204/212 | 229/229 | 130/130 | 191/203 | 135/135 | 170/192 | 154/170 | 140/152 | 243/243 | 122/144 | 172/174 | 183/185 | 210/210 | 207/212 | 141/144 | 197/199 | 174/186 | 125/125 | 173/173 | 166/175 |
| 12 | C | 12 | 204/212 | 229/229 | 129/186 | 197/203 | 125/151 | 170/214 | 154/170 | 140/152 | 243/243 | 144/148 | 170/172 | 183/185 | 206/210 | 210/212 | 141/144 | 191/197 | 141/157 | 125/125 | 152/173 | 166/194 |
| 13 | C | 13 | 212/214 | 229/229 | 129/186 | 197/203 | 125/135 | 170/214 | 230/230 | 146/178 | 243/243 | 122/122 | 172/174 | 183/185 | 206/206 | 207/212 | 141/144 | 191/199 | 157/190 | 114/131 | 160/160 | 166/194 |
| 14 | C | 14 | 204/212 | 229/234 | 130/159 | 203/205 | 129/134 | 160/184 | 182/182 | 146/160 | 243/243 | 122/161 | 166/174 | 189/193 | 216/216 | 212/212 | 124/124 | 191/201 | 147/171 | 116/119 | 157/168 | 175/175 |
| 15 | C | 15 | 204/206 | 229/234 | 130/159 | 197/203 | 134/141 | 174/184 | 152/152 | 146/180 | 243/243 | 152/173 | 168/172 | 183/193 | 216/216 | 212/212 | 118/121 | 191/199 | 137/147 | 114/127 | 160/160 | 172/175 |
| 16 | C | 16 | 204/206 | 229/234 | 159/163 | 197/201 | 129/143 | 174/202 | 152/152 | 140/180 | 243/243 | 122/173 | 162/174 | 181/183 | 206/206 | 210/212 | 124/147 | 189/199 | 137/171 | 114/116 | 152/160 | 169/216 |
| **17** | **C & M** | **17** | **204/204** | **229/237** | **159/161** | **203/203** | **129/135** | **160/184** | **194/194** | **140/182** | **243/243** | **122/148** | **174/180** | **183/193** | **206/210** | **212/212** | **121/141** | **191/217** | **133/147** | **114/127** | **160/168** | **175/175** |
| 18 | C | 18 | 212/212 | 229/237 | 130/186 | 191/197 | 125/134 | 170/170 | 170/170 | 140/178 | 263/263 | 122/144 | 170/174 | 183/185 | 210/210 | 210/212 | 121/141 | 191/197 | 157/174 | 125/131 | 152/157 | 166/212 |
| 19 | C | 19 | 212/214 | 229/237 | 130/138 | 195/203 | 129/159 | 178/182 | 152/152 | 134/174 | 243/243 | 148/159 | 168/176 | 183/185 | 210/210 | 212/212 | 118/141 | 183/191 | 147/161 | 112/125 | 154/154 | 177/212 |
| **20** | **C & M** | **20** | **204/212** | **229/237** | **129/138** | **203/209** | **129/134** | **180/204** | **208/208** | **140/140** | **243/254** | **122/165** | **170/172** | **185/193** | **216/218** | **207/212** | **124/141** | **191/197** | **147/176** | **114/129** | **152/160** | **175/177** |
| 21 | C | 21 | 212/214 | 229/237 | 129/130 | 203/203 | 129/137 | 182/184 | 152/152 | 140/178 | 243/243 | 122/152 | 168/172 | 183/193 | 210/216 | 212/212 | 121/127 | 191/199 | 147/184 | 114/116 | 154/160 | 172/175 |
| 22 | C | 22 | 204/223 | 229/237 | 136/186 | 195/199 | 134/141 | 182/204 | 160/188 | 140/178 | 263/263 | 148/148 | 170/180 | 185/185 | 212/212 | 212/240 | 124/127 | 183/197 | 157/167 | 131/134 | 152/154 | 175/187 |
| 23 | C | 23 | 204/204 | 229/237 | 129/186 | 191/203 | 129/134 | 184/204 | 152/152 | 140/146 | 243/243 | 122/148 | 168/172 | 185/193 | 216/216 | 212/212 | 121/127 | 191/197 | 147/174 | 116/127 | 160/168 | 175/175 |
| **24** | **C & M** | **24** | **204/204** | **229/237** | **129/184** | **191/203** | **129/134** | **184/204** | **152/152** | **140/146** | **243/243** | **122/148** | **168/172** | **185/193** | **216/216** | **212/212** | **121/127** | **191/197** | **147/171** | **116/127** | **160/168** | **175/175** |
| **25** | **C & M** | **25** | **204/204** | **229/237** | **130/159** | **191/203** | **135/135** | **192/192** | **154/154** | **140/146** | **243/243** | **122/173** | **170/174** | **189/189** | **210/212** | **207/225** | **118/124** | **191/205** | **133/155** | **116/131** | **168/168** | **175/214** |
| 26 | C | 26 | 214/214 | 229/237 | 132/138 | 203/203 | 129/135 | 192/192 | 152/156 | 130/134 | 243/267 | 124/173 | 172/176 | 181/183 | 210/210 | 207/212 | 118/118 | 189/199 | 171/180 | 112/119 | 154/154 | 208/214 |
| 27 | C | 27 | 204/212 | 229/241 | 138/186 | 203/211 | 137/151 | 164/170 | 156/156 | 130/178 | 263/267 | 148/209 | 172/174 | 183/189 | 206/218 | 207/212 | 124/141 | 183/205 | 147/157 | 116/119 | 168/168 | 175/202 |
| 28 | C | 28 | 204/204 | 229/241 | 159/159 | 203/209 | 129/129 | 170/192 | 162/162 | 140/146 | 243/243 | 144/152 | 158/166 | 189/189 | 206/220 | 207/212 | 121/141 | 189/191 | 147/169 | 127/129 | 160/160 | 170/214 |
| **29** | **C & M** | **29** | **212/212** | **229/241** | **159/186** | **203/203** | **129/135** | **170/192** | **152/152** | **140/178** | **243/243** | **122/148** | **166/168** | **183/193** | **210/216** | **212/212** | **121/141** | **191/199** | **147/184** | **114/127** | **157/160** | **175/212** |
| 30 | C | 30 | 212/212 | 229/241 | 159/186 | 203/203 | 129/135 | 170/192 | 152/152 | 140/178 | 243/243 | 122/148 | 166/168 | 183/193 | 210/216 | 212/212 | 121/141 | 191/199 | 147/184 | 114/127 | 157/160 | 175/214 |
| 31 | C | 31 | 204/212 | 229/241 | 129/130 | 203/203 | 129/137 | 170/202 | 152/152 | 140/146 | 243/243 | 122/122 | 166/168 | 189/193 | 210/216 | 212/212 | 121/127 | 191/205 | 147/147 | 116/127 | 160/168 | 175/214 |
| 32 | C | 32 | 204/212 | 229/241 | 129/130 | 191/205 | 129/134 | 182/184 | 152/168 | 130/140 | 243/254 | 122/147 | 172/174 | 185/193 | 216/220 | 212/242 | 121/141 | 191/197 | 147/171 | 114/131 | 154/160 | 175/214 |
| **33** | **C & M** | **33** | **204/214** | **229/241** | **130/159** | **199/203** | **137/137** | **182/204** | **162/162** | **140/178** | **243/243** | **122/144** | **164/174** | **183/189** | **206/220** | **212/212** | **121/141** | **183/205** | **147/157** | **116/129** | **157/168** | **175/175** |
| **34** | **C & M** | **34** | **212/214** | **229/241** | **129/129** | **203/203** | **137/139** | **182/204** | **162/162** | **140/178** | **243/254** | **144/148** | **174/180** | **183/189** | **210/220** | **212/212** | **121/127** | **191/205** | **147/147** | **116/134** | **154/168** | **175/216** |
| 35 | C | 35 | 212/214 | 229/241 | 129/186 | 203/203 | 137/139 | 182/208 | 162/162 | 140/178 | 243/254 | 144/148 | 174/180 | 183/189 | 210/220 | 212/212 | 121/127 | 191/205 | 147/147 | 116/134 | 154/168 | 175/216 |
| 36 | C | 36 | 204/204 | 234/243 | 129/186 | 191/203 | 129/134 | 192/192 | 152/152 | 140/146 | 243/243 | 124/154 | 172/178 | 185/193 | 206/216 | 212/212 | 121/127 | 191/197 | 147/171 | 116/127 | 160/168 | 172/214 |
| 37 | C | 37 | 204/212 | 229/241 | 159/186 | 203/205 | 129/137 | 184/204 | 152/152 | 146/176 | 243/243 | 122/152 | 166/174 | 189/193 | 210/216 | 212/212 | 124/127 | 191/205 | 147/147 | 125/127 | 160/160 | 214/216 |
| 38 | C | 38 | 204/204 | 229/241 | 129/130 | 191/203 | 129/134 | 184/204 | 162/162 | 140/146 | 243/243 | 122/152 | 168/180 | 181/189 | 206/210 | 212/212 | 121/127 | 189/205 | 171/171 | 114/116 | 160/168 | 185/210 |
| 39 | C | 39 | 204/214 | 229/241 | 132/159 | 203/203 | 129/137 | 192/204 | 152/162 | 134/140 | 243/243 | 122/124 | 164/176 | 181/183 | 210/220 | 207/212 | 118/141 | 183/189 | 157/171 | 116/119 | 154/168 | 175/208 |
| 40 | C | 40 | 204/204 | 229/243 | 138/161 | 203/203 | 135/135 | 160/206 | 146/158 | 178/178 | 243/243 | 124/152 | 168/172 | 183/185 | 210/220 | 207/207 | 121/141 | 199/217 | 133/133 | 103/119 | 152/154 | 172/210 |
| 41 | C | 41 | 204/212 | 229/243 | 159/186 | 191/205 | 129/134 | 170/184 | 152/162 | 130/146 | 243/243 | 122/144 | 166/168 | 181/181 | 206/220 | 212/212 | 121/121 | 189/197 | 171/171 | 116/127 | 160/168 | 170/175 |
| 42 | C | 42 | 204/212 | 229/243 | 159/182 | 191/205 | 129/134 | 174/184 | 152/162 | 130/146 | 243/243 | 122/144 | 166/168 | 181/181 | 206/220 | 212/212 | 121/121 | 189/197 | 171/171 | 116/127 | 160/168 | 170/175 |
| 43 | C | 43 | 204/204 | 229/243 | 145/159 | 203/203 | 134/151 | 180/202 | 182/182 | 146/152 | 243/245 | 122/224 | 166/174 | 181/183 | 206/210 | 212/212 | 124/141 | 189/197 | 137/141 | 119/127 | 152/152 | 216/218 |
| **44** | **C & M** | **44** | **204/212** | **237/237** | **159/159** | **203/203** | **129/135** | **192/204** | **156/218** | **140/178** | **243/243** | **122/148** | **166/174** | **181/189** | **206/206** | **212/212** | **124/127** | **189/199** | **171/184** | **114/119** | **152/160** | **175/210** |
| 45 | C | 45 | 212/214 | 229/229 | 129/159 | 203/203 | 129/129 | 182/184 | 162/162 | 140/146 | 243/243 | 122/173 | 172/174 | 183/185 | 206/220 | 207/212 | 118/141 | 183/197 | 157/171 | 119/129 | 152/157 | 206/206 |
| **46** | **C & M** | **46** | **204/212** | **229/243** | **129/159** | **203/205** | **129/129** | **184/202** | **152/152** | **140/146** | **243/243** | **122/152** | **166/172** | **181/193** | **206/216** | **212/212** | **121/124** | **189/191** | **147/171** | **114/127** | **160/160** | **175/216** |
| 47 | C | 47 | 204/212 | 229/243 | 129/159 | 203/205 | 129/129 | 192/202 | 152/152 | 140/146 | 243/243 | 122/152 | 166/172 | 181/193 | 206/216 | 212/212 | 121/124 | 189/191 | 147/171 | 114/127 | 160/160 | 175/214 |
| 48 | C | 48 | 204/212 | 229/243 | 129/159 | 203/203 | 129/129 | 192/202 | 152/152 | 140/146 | 243/243 | 122/152 | 166/172 | 181/193 | 206/216 | 212/212 | 121/124 | 189/191 | 147/171 | 114/127 | 160/160 | 175/214 |
| 49 | C | 49 | 204/212 | 229/243 | 129/159 | 203/205 | 129/129 | 192/202 | 152/152 | 140/146 | 243/243 | 122/152 | 166/172 | 181/193 | 206/216 | 212/212 | 121/124 | 189/191 | 147/174 | 114/127 | 160/160 | 175/216 |
| **50** | **C & M** | **50** | **204/212** | **229/229** | **129/130** | **191/203** | **129/137** | **184/192** | **152/194** | **140/146** | **243/254** | **148/152** | **166/168** | **181/183** | **206/210** | **212/212** | **121/141** | **189/191** | **147/157** | **114/125** | **152/160** | **166/216** |
| 51 | C | 51 | 212/214 | 229/229 | 142/157 | 197/203 | 129/155 | 184/192 | 194/194 | 126/140 | 243/243 | 122/148 | 166/176 | 181/185 | 206/226 | 207/212 | 124/124 | 183/189 | 171/171 | 127/129 | 152/160 | 175/175 |
| 52 | C | 52 | 212/212 | 229/247 | 159/161 | 203/207 | 137/137 | 160/204 | 162/162 | 140/178 | 243/243 | 124/152 | 166/176 | 185/189 | 206/210 | 212/212 | 121/141 | 197/205 | 147/184 | 114/119 | 152/160 | 175/208 |
| **53** | **C & M** | **53** | **204/214** | **229/247** | **129/186** | **191/203** | **129/135** | **170/192** | **152/152** | **156/180** | **243/243** | **124/161** | **168/176** | **183/189** | **210/226** | **207/212** | **121/121** | **197/199** | **161/171** | **103/116** | **168/168** | **175/175** |
| 54 | C | 54 | 204/204 | 229/247 | 129/161 | 203/205 | 129/134 | 174/202 | 204/204 | 140/146 | 243/243 | 122/124 | 166/172 | 183/193 | 206/210 | 212/212 | 124/124 | 191/197 | 147/171 | 114/114 | 160/160 | 210/216 |
| 55 | C | 55 | 204/214 | 229/247 | 159/159 | 203/203 | 123/137 | 182/192 | 162/162 | 134/140 | 243/243 | 144/144 | 166/174 | 183/183 | 218/220 | 212/212 | 118/121 | 183/189 | 157/157 | 103/129 | 157/157 | 172/172 |
| 56 | C | 56 | 204/212 | 229/247 | 129/152 | 203/205 | 129/141 | 186/192 | 152/152 | 140/160 | 243/243 | 124/152 | 172/178 | 183/193 | 216/216 | 212/212 | 124/124 | 191/199 | 141/147 | 114/119 | 152/160 | 172/175 |
| 57 | C | 57 | 204/212 | 229/247 | 129/129 | 203/203 | 129/137 | 186/190 | 218/218 | 146/176 | 263/263 | 122/177 | 168/178 | 183/198 | 210/210 | 212/212 | 118/124 | 189/205 | 137/171 | 114/127 | 154/160 | 175/216 |
| **58** | **C & M** | **58** | **204/204** | **229/247** | **130/161** | **203/205** | **134/135** | **192/206** | **158/158** | **160/178** | **243/243** | **124/152** | **168/172** | **183/185** | **210/210** | **207/212** | **121/141** | **197/199** | **133/171** | **103/119** | **152/154** | **175/210** |
| 59 | C | 59 | 204/204 | 229/247 | 129/161 | 203/203 | 129/135 | 194/198 | 152/152 | 146/146 | 243/243 | 124/152 | 166/172 | 183/193 | 210/216 | 212/212 | 124/141 | 191/199 | 147/184 | 114/119 | 152/160 | 172/216 |
| 60 | C | 60 | 204/212 | 243/251 | 130/130 | 203/203 | 137/137 | 160/204 | 194/219 | 140/180 | 243/254 | 152/173 | 166/172 | 183/185 | 210/210 | 207/212 | 127/141 | 197/217 | 133/147 | 116/119 | 152/168 | 172/216 |
| 61 | M | 61 | 204/204 | 229/251 | 129/161 | 191/203 | 134/135 | 160/196 | 152/152 | 146/176 | 243/243 | 124/152 | 174/176 | 183/193 | 210/216 | 212/212 | 118/127 | 191/217 | 133/171 | 114/119 | 152/160 | 172/175 |
| 62 | C | 62 | 204/204 | 229/247 | 130/157 | 203/207 | 133/134 | 196/202 | 154/154 | 146/182 | 243/243 | 154/211 | 166/189 | 183/185 | 216/216 | 207/212 | 118/121 | 197/197 | 137/174 | 114/119 | 152/160 | 169/208 |
| **63** | **C & M** | **63** | **204/204** | **229/253** | **159/161** | **203/203** | **134/139** | **170/192** | **154/154** | **140/160** | **243/243** | **122/173** | **166/180** | **183/185** | **210/210** | **207/212** | **118/124** | **197/199** | **171/184** | **119/125** | **152/152** | **172/175** |
| 64 | C | 64 | 204/204 | 229/253 | 159/161 | 203/203 | 123/129 | 186/204 | 182/182 | 146/168 | 243/243 | 122/177 | 166/174 | 181/183 | 206/216 | 210/212 | 121/124 | 191/193 | 147/204 | 119/127 | 152/160 | 172/175 |
| 65 | C | 65 | 204/204 | 229/251 | 129/161 | 191/203 | 134/135 | 160/198 | 152/152 | 146/178 | 243/243 | 124/152 | 174/176 | 183/193 | 210/216 | 212/212 | 118/127 | 191/217 | 133/171 | 114/119 | 152/160 | 172/175 |
| 66 | C | 66 | 204/212 | 229/251 | 129/161 | 191/203 | 134/135 | 160/196 | 152/152 | 146/178 | 243/243 | 124/152 | 174/176 | 183/193 | 210/216 | 212/212 | 118/127 | 191/217 | 133/171 | 114/119 | 152/160 | 172/175 |
| **67** | **C & M** | **67** | **204/214** | **237/247** | **130/161** | **203/203** | **139/139** | **182/192** | **154/154** | **178/182** | **243/243** | **122/124** | **168/172** | **183/183** | **210/210** | **212/212** | **118/127** | **191/217** | **133/133** | **116/119** | **152/168** | **172/212** |
| **68** | **C & M** | **68** | **204/204** | **229/251** | **129/152** | **203/205** | **125/129** | **160/202** | **152/152** | **146/146** | **243/243** | **152/177** | **166/170** | **181/193** | **210/216** | **212/212** | **118/124** | **191/195** | **153/171** | **119/127** | **152/160** | **172/225** |
| 69 | C | 69 | 204/204 | 229/251 | 154/154 | 203/203 | 135/135 | 164/192 | 174/174 | 134/160 | 262/263 | 122/124 | 180/183 | 183/193 | 206/210 | 212/212 | 118/124 | 191/199 | 159/184 | 114/119 | 152/160 | 208/214 |
| 70 | C | 70 | 204/214 | 229/251 | 130/154 | 203/203 | 129/139 | 170/178 | 156/156 | 140/160 | 254/254 | 122/173 | 166/168 | 181/183 | 206/206 | 207/212 | 124/127 | 189/191 | 147/159 | 119/119 | 152/152 | 185/216 |
| 71 | C | 71 | 204/204 | 229/251 | 159/161 | 203/203 | 135/135 | 170/196 | 154/154 | 140/160 | 243/243 | 122/173 | 166/180 | 183/185 | 210/210 | 207/212 | 118/124 | 197/199 | 171/184 | 119/125 | 152/152 | 172/175 |
| 72 | C | 72 | 204/204 | 229/251 | 161/161 | 203/203 | 123/123 | 184/206 | 184/184 | 142/146 | 243/243 | 122/122 | 174/174 | 181/183 | 206/216 | 210/212 | 121/135 | 191/191 | 141/208 | 119/127 | 152/160 | 175/177 |
| 73 | C | 73 | 204/265 | 229/251 | 159/159 | 199/203 | 135/137 | 192/192 | 194/218 | 130/178 | 243/243 | 144/173 | 166/176 | 183/189 | 210/210 | 212/212 | 121/141 | 199/205 | 149/188 | 114/127 | 157/160 | 208/216 |
| **74** | **C & M** | **74** | **204/265** | **229/251** | **159/159** | **199/203** | **135/137** | **192/192** | **194/218** | **130/178** | **243/243** | **144/173** | **166/176** | **183/189** | **210/210** | **212/212** | **121/141** | **199/205** | **147/188** | **114/127** | **157/160** | **208/216** |
| **75** | **C & M** | **75** | **204/214** | **237/247** | **138/165** | **203/203** | **135/139** | **192/204** | **154/154** | **160/178** | **243/243** | **124/152** | **168/176** | **183/185** | **210/220** | **212/212** | **118/121** | **197/199** | **184/184** | **119/134** | **152/154** | **172/216** |
| 76 | C | 76 | 204/204 | 231/237 | 138/186 | 195/203 | 129/134 | 192/204 | 178/178 | 130/140 | 243/243 | 122/157 | 170/172 | 181/193 | 216/220 | 207/212 | 121/121 | 191/191 | 147/171 | 116/131 | 168/168 | 175/198 |
| 77 | C | 77 | 204/204 | 231/247 | 116/129 | 203/207 | 134/139 | 174/192 | 152/152 | 178/182 | 243/243 | 124/124 | 172/172 | 183/208 | 206/210 | 212/212 | 118/124 | 197/203 | 147/171 | 114/119 | 160/160 | 170/212 |
| 78 | C | 78 | 204/204 | 232/253 | 159/161 | 203/203 | 129/137 | 170/202 | 182/190 | 142/146 | 243/243 | 122/122 | 174/174 | 181/196 | 216/216 | 207/212 | 121/135 | 191/217 | 147/147 | 119/119 | 152/152 | 170/175 |
| 79 | C | 79 | 204/204 | 232/251 | 159/161 | 203/203 | 123/135 | 186/186 | 180/180 | 142/168 | 243/243 | 122/177 | 166/174 | 183/183 | 216/216 | 210/210 | 124/135 | 193/193 | 133/204 | 119/127 | 152/160 | 172/175 |
| 80 | C | 80 | 204/204 | 232/251 | 129/130 | 203/203 | 125/168 | 186/200 | 188/188 | 140/160 | 245/245 | 124/173 | 183/191 | 181/191 | 210/210 | 207/210 | 124/124 | 195/195 | 147/180 | 121/123 | 152/152 | 172/172 |
| 81 | C | 81 | 204/272 | 234/241 | 129/130 | 195/203 | 134/139 | 180/208 | 176/176 | 130/178 | 243/243 | 148/154 | 172/174 | 183/189 | 206/210 | 212/221 | 121/141 | 183/197 | 159/171 | 114/125 | 160/160 | 175/214 |
| 82 | C | 82 | 204/252 | 234/241 | 129/130 | 195/203 | 134/139 | 180/204 | 176/176 | 130/178 | 243/243 | 148/154 | 172/174 | 183/189 | 206/210 | 212/221 | 121/141 | 183/197 | 159/171 | 114/125 | 160/160 | 175/214 |
| **83** | **C & M** | **83** | **204/268** | **234/241** | **129/130** | **195/203** | **134/139** | **180/204** | **176/176** | **130/178** | **243/243** | **148/154** | **172/174** | **183/189** | **206/210** | **212/221** | **121/141** | **183/197** | **159/171** | **114/125** | **160/160** | **175/212** |
| 84 | C | 84 | 204/214 | 237/247 | 130/130 | 203/203 |  | 182/190 | 154/154 | 140/160 | 263/263 | 152/175 | 166/172 | 183/183 | 210/220 | 207/212 | 118/121 | 191/199 | 184/184 | 116/119 |  | 208/212 |
| 85 | C | 85 | 204/274 | 234/241 | 129/130 | 195/203 | 134/139 | 180/204 | 176/176 | 130/178 | 243/243 | 148/154 | 174/174 | 183/189 | 206/210 | 212/221 | 121/141 | 183/197 | 159/171 | 114/125 | 160/160 | 175/214 |
| 86 | C | 86 | 206/212 | 234/243 | 152/163 | 203/205 | 129/137 | 170/192 | 152/152 | 178/182 | 243/243 | 124/173 | 172/172 | 181/187 | 210/216 | 212/212 | 121/124 | 191/217 | 147/171 | 116/127 | 152/160 | 170/218 |
| 87 | C | 87 | 204/204 | 229/247 | 129/129 | 203/203 |  | 192/204 |  | 146/160 | 243/243 | 122/173 | 168/172 | 183/189 | 206/210 | 207/212 | 124/141 | 205/217 | 133/171 | 114/119 |  | 175/210 |
| 88 | C | 88 | 206/212 | 234/243 | 129/196 | 201/203 |  | 170/192 |  | 140/162 | 243/243 | 124/173 | 185/189 | 181/187 | 210/210 | 212/212 | 118/121 | 191/205 | 147/171 | 116/119 |  | 170/216 |
| 89 | C | 89 | 204/204 | 234/243 | 130/198 | 201/203 |  | 170/202 |  | 146/166 | 243/243 | 173/199 | 172/172 | 183/187 | 210/216 | 212/212 | 118/121 | 197/205 | 137/141 | 114/114 |  | 170/216 |
| 90 | C | 90 | 204/212 | 234/243 | 163/163 | 203/207 | 129/137 | 192/192 | 152/152 | 146/178 | 263/263 | 124/154 | 172/178 | 181/198 | 206/210 | 212/212 | 124/124 | 189/205 | 137/147 | 114/127 | 152/160 | 172/214 |
| 91 | C | 91 | 206/206 | 234/249 | 152/198 | 201/205 | 135/135 | 170/196 | 216/260 | 162/178 | 243/243 | 124/173 | 172/189 | 187/196 | 206/210 | 212/212 | 118/124 | 205/217 | 141/171 | 114/116 | 152/160 | 170/172 |
| **92** | **C & M** | **92** | **204/212** | **234/251** | **130/130** | **195/203** | **135/137** | **160/182** | **158/158** | **140/160** | **263/267** | **144/173** | **176/176** | **183/185** | **210/220** | **207/212** | **118/121** | **197/217** | **133/147** | **119/134** | **152/154** | **172/172** |
| 93 | C | 93 | 204/206 | 234/251 | 159/159 | 197/197 | 135/143 | 174/174 | 152/152 | 180/180 | 243/243 | 122/228 | 178/189 | 181/187 | 204/210 | 210/212 | 118/147 | 189/205 | 137/137 | 114/119 | 152/160 | 175/204 |
| 94 | C | 94 | 204/204 | 234/251 | 152/159 | 203/203 | 137/141 | 174/186 | 216/216 | 160/180 | 263/263 | 173/177 | 168/170 | 183/198 | 210/210 | 212/212 | 118/118 | 199/205 | 137/137 | 119/127 | 152/152 | 172/210 |
| **95** | **C & M** | **95** | **204/268** | **234/251** | **129/161** | **195/203** | **123/139** | **180/192** | **158/158** | **130/140** | **243/243** | **154/173** | **174/180** | **183/185** | **206/210** | **207/212** | **121/127** | **183/189** | **157/159** | **119/125** | **152/152** | **175/214** |
| **96** | **C & M** | **96** | **212/268** | **234/251** | **129/129** | **203/203** | **135/135** | **180/204** | **176/176** | **130/140** | **243/254** | **144/154** | **172/174** | **183/189** | **206/210** | **212/221** | **121/141** | **197/199** | **171/184** | **114/127** | **157/160** | **170/214** |
| **97** | **C & M** | **97** | **212/268** | **234/251** | **129/129** | **203/203** | **135/135** | **180/204** | **176/176** | **130/140** | **243/243** | **144/154** | **172/174** | **183/189** | **206/210** | **212/221** | **121/141** | **197/199** | **171/186** | **114/127** | **157/160** | **170/214** |
| **98** | **C & M** | **98** | **212/268** | **234/251** | **129/130** | **203/203** | **139/139** | **204/204** | **154/176** | **130/140** | **254/254** | **148/171** | **172/172** | **183/183** | **206/210** | **212/221** | **141/141** | **183/195** | **159/184** | **125/134** | **154/154** | **175/210** |
| 99 | C | 99 | 204/212 | 237/237 | 130/165 | 203/205 | 133/134 | 160/204 | 176/176 | 160/178 | 263/263 | 143/173 | 172/193 | 183/185 | 210/220 | 212/212 | 121/124 | 189/197 | 171/176 | 119/131 | 152/154 | 175/179 |
| **100** | **C & M** | **100** | **212/214** | **237/237** | **130/148** | **199/203** | **134/151** | **170/182** | **162/162** | **140/146** | **243/254** | **148/161** | **162/174** | **185/185** | **210/220** | **207/212** | **124/141** | **183/197** | **157/171** | **103/125** | **154/154** | **175/185** |
| 101 | C | 101 | 204/212 | 237/241 | 129/192 | 195/203 | 129/135 | 160/192 | 154/154 | 146/160 | 243/243 | 148/148 | 172/176 | 181/183 | 206/216 | 212/212 | 118/124 | 189/217 | 133/171 | 114/125 | 160/160 | 185/214 |
| **102** | **C & M** | **102** | **204/212** | **237/241** | **129/130** | **203/209** | **129/137** | **178/204** | **178/178** | **140/174** | **243/243** | **122/159** | **166/168** | **185/193** | **206/206** | **212/212** | **124/141** | **191/197** | **147/147** | **125/131** | **154/154** | **170/214** |
| **103** | **C & M** | **103** | **204/268** | **237/241** | **129/161** | **195/203** | **139/139** | **180/192** | **176/176** | **130/170** | **243/243** | **148/171** | **172/176** | **183/189** | **210/210** | **207/221** | **121/141** | **197/217** | **133/159** | **114/131** | **154/160** | **175/198** |
| 104 | M | 104 | 212/214 | 229/241 | 129/186 | 203/203 | 137/139 | 182/204 | 164/164 | 140/178 | 243/254 | 144/148 | 174/180 | 183/189 | 210/220 | 212/212 | 121/127 | 191/205 | 147/147 | 116/134 | 154/168 | 175/216 |
| 105 | C | 105 | 212/214 | 237/241 | 184/186 | 195/203 | 149/159 | 184/184 | 146/146 | 126/130 | 243/243 | 122/173 | 162/168 | 183/183 | 206/220 | 207/212 | 124/124 | 183/191 | 157/159 | 114/119 | 152/160 | 208/216 |
| 106 | C | 106 | 204/240 | 237/237 | 130/152 | 191/191 | 137/145 | 180/192 | 156/156 | 140/146 | 261/263 | 144/148 | 168/170 | 183/189 | 206/210 | 212/212 | 121/127 | 191/205 | 147/157 | 125/125 | 152/152 | 170/206 |
| 107 | C | 107 | 204/204 | 237/241 | 163/163 | 203/205 | 135/135 | 192/192 | 154/154 | 160/160 | 243/243 | 148/173 | 172/176 | 183/185 | 210/220 | 212/212 | 118/141 | 197/217 | 133/133 | 119/134 | 152/154 | 172/172 |
| **108** | **C & M** | **108** | **204/240** | **237/237** | **130/152** | **191/191** | **137/145** | **180/204** | **156/156** | **140/146** | **261/263** | **144/148** | **168/170** | **183/189** | **206/210** | **212/212** | **121/127** | **191/205** | **147/157** | **123/125** | **152/152** | **170/214** |
| 109 | C | 109 | 204/204 | 237/243 | 136/136 | 203/205 | 141/141 | 160/170 | 154/154 | 146/178 | 243/243 | 144/173 | 166/172 | 183/189 | 210/210 | 207/207 | 118/141 | 191/205 | 147/155 | 127/131 | 154/160 | 177/181 |
| **110** | **C & M** | **110** | **204/212** | **237/243** | **142/154** | **203/205** | **129/135** | **170/180** | **140/152** | **126/160** | **243/263** | **154/173** | **166/172** | **181/183** | **206/226** | **207/212** | **124/126** | **189/199** | **133/147** | **119/131** | **152/152** | **214/216** |
| 111 | C | 111 | 204/204 | 237/243 | 129/161 | 203/203 | 129/134 | 170/184 | 194/238 | 130/146 | 263/263 | 126/173 | 158/172 | 183/189 | 206/206 | 212/212 | 121/121 | 191/191 | 147/171 | 119/119 | 152/152 | 175/175 |
| **112** | **C & M** | **112** | **212/214** | **237/243** | **163/186** | **199/203** | **129/159** | **170/184** | **140/140** | **126/140** | **243/243** | **122/148** | **162/166** | **181/183** | **206/220** | **207/212** | **121/124** | **189/191** | **157/176** | **114/125** | **160/160** | **214/216** |
| 113 | C | 113 | 204/214 | 237/243 | 130/142 | 199/203 | 129/139 | 174/182 | 156/156 | 140/140 | 243/254 | 124/152 | 166/170 | 181/183 | 206/220 | 212/212 | 121/124 | 189/191 | 147/147 | 114/134 | 154/160 | 194/216 |
| **114** | **C & M** | **114** | **212/214** | **237/243** | **130/138** | **203/203** | **137/139** | **182/204** | **194/194** | **140/178** | **254/254** | **122/152** | **166/168** | **183/185** | **210/220** | **212/212** | **121/127** | **191/197** | **147/147** | **116/134** | **154/168** | **212/216** |
| 115 | C | 115 | 212/229 | 237/243 | 136/136 | 203/211 | 135/135 | 182/190 | 156/156 | 178/178 | 243/243 | 163/173 | 168/168 | 183/183 | 210/220 | 212/225 | 121/124 | 199/217 | 133/184 | 116/119 | 152/168 | 170/212 |
| **116** | **C & M** | **116** | **204/204** | **237/243** | **129/130** | **203/205** | **129/135** | **184/192** | **152/152** | **146/178** | **243/243** | **122/173** | **172/172** | **181/183** | **210/216** | **212/212** | **124/127** | **189/217** | **133/147** | **114/119** | **152/160** | **175/214** |
| 117 | C | 117 | 204/212 | 237/243 |  | 205/205 | 125/134 | 192/192 | 158/158 | 146/146 | 243/243 | 124/124 | 166/168 | 183/198 | 212/216 | 207/207 | 118/124 | 197/205 | 155/171 | 116/127 | 154/154 | 169/216 |
| 118 | C | 118 | 204/212 | 237/243 | 159/186 | 191/205 | 129/134 | 202/204 | 152/152 | 140/178 | 243/243 | 148/152 | 166/168 | 181/185 | 210/216 | 212/212 | 124/141 | 189/197 | 171/171 | 116/127 | 160/168 | 175/216 |
| 119 | C | 119 | 204/204 | 237/247 | 130/161 | 191/203 | 137/137 | 160/170 | 154/154 | 182/182 | 243/243 | 124/148 | 174/180 | 183/189 | 210/210 | 212/212 | 127/141 | 205/217 | 133/147 | 116/127 | 157/168 | 175/198 |
| 120 | C | 120 | 204/214 | 237/247 | 138/138 | 203/203 | 135/137 | 160/182 | 154/154 | 140/160 | 254/254 | 122/124 | 166/176 | 183/185 | 210/210 | 207/212 | 127/141 | 197/199 | 147/184 | 127/131 | 154/157 | 172/212 |
| **121** | **C & M** | **121** | **204/212** | **237/247** | **130/161** | **203/203** | **135/139** | **160/182** | **218/218** | **178/178** | **243/243** | **122/124** | **166/172** | **183/183** | **210/220** | **207/212** | **118/127** | **191/199** | **133/133** | **127/134** | **154/157** | **212/218** |
| 122 | C | 122 | 204/214 | 237/247 | 130/161 | 203/203 |  | 160/182 |  | 140/160 | 263/263 | 152/173 | 166/176 |  | 210/220 | 207/212 | 121/141 | 191/217 | 133/133 | 116/127 |  | 204/208 |
| **123** | **C & M** | **123** | **204/214** | **237/247** | **130/161** | **203/203** | **139/139** | **160/182** | **154/154** | **140/178** | **254/254** | **122/124** | **168/172** | **183/183** | **210/220** | **212/212** | **118/127** | **191/217** | **133/133** | **119/134** | **152/154** | **208/216** |
| 124 | C | 125 | 204/204 | 243/247 | 130/159 | 203/207 |  | 160/202 |  | 146/156 | 243/243 | 122/222 | 166/172 | 181/183 | 210/216 | 212/212 | 121/141 | 189/197 | 137/171 | 114/119 |  | 208/210 |
| 125 | M | 126 | 204/204 | 241/247 | 161/186 | 191/203 | 134/135 | 160/170 | 152/152 | 140/178 | 243/263 | 148/173 | 168/172 | 183/185 | 210/210 | 212/212 | 118/124 | 197/199 | 171/184 | 114/119 | 152/160 | 175/208 |
| 126 | C | 127 | 204/212 | 237/247 | 130/161 | 203/203 | 135/137 | 160/184 | 154/154 | 140/160 | 243/254 | 152/173 | 166/172 | 183/185 | 194/210 | 207/212 | 118/121 | 197/199 | 147/184 | 119/134 | 152/154 | 172/212 |
| 127 | C | 128 | 204/214 | 237/247 | 138/138 | 203/203 | 137/137 | 160/204 | 154/154 | 140/160 | 254/254 | 152/173 | 166/172 | 183/185 | 210/220 | 212/212 | 118/121 | 197/207 | 133/147 | 116/119 | 152/168 | 172/210 |
| 128 | C | 129 | 204/204 | 229/241 | 182/190 | 191/203 | 137/145 | 180/204 | 156/156 | 140/146 | 261/263 | 122/144 | 158/166 | 183/185 | 206/206 | 212/212 | 127/141 | 191/205 | 147/157 | 114/125 | 160/160 | 185/185 |
| **129** | **C & M** | **130** | **204/214** | **237/247** | **138/161** | **203/203** | **137/137** | **160/204** | **194/219** | **140/160** | **254/254** | **122/124** | **168/172** | **183/185** | **210/220** | **207/212** | **118/121** | **197/217** | **133/147** | **119/134** | **152/154** | **172/216** |
| **130** | **C & M** | **131** | **204/212** | **237/247** | **138/138** | **203/203** | **135/137** | **160/208** | **218/218** | **160/178** | **243/243** | **152/177** | **168/176** | **183/185** | **210/220** | **212/212** | **127/141** | **197/199** | **147/184** | **119/134** | **152/154** | **172/216** |
| **131** | **C & M** | **132** | **212/214** | **237/247** | **130/163** | **203/203** | **129/134** | **170/184** | **140/162** | **126/178** | **243/243** | **144/148** | **162/162** | **181/185** | **206/210** | **207/212** | **124/141** | **189/197** | **133/171** | **114/116** | **160/168** | **214/221** |
| 132 | C | 133 | 212/212 | 237/247 | 161/186 | 191/203 | 135/135 | 170/192 | 154/154 | 160/178 | 243/243 | 124/148 | 176/176 | 183/189 | 210/210 | 207/207 | 127/141 | 197/199 | 171/182 | 127/131 | 152/157 | 172/214 |
| 133 | C | 134 | 212/212 | 237/247 | 161/186 | 191/203 | 135/135 | 170/192 | 154/154 | 160/178 | 243/243 | 124/148 | 176/176 | 183/189 | 210/210 | 207/207 | 127/141 | 197/199 | 171/184 | 127/131 | 152/157 | 172/214 |
| 134 | C | 135 | 204/214 | 237/247 | 130/161 | 203/203 | 139/139 | 174/192 | 154/154 | 140/178 | 243/254 | 122/173 | 168/172 | 183/183 | 210/220 | 212/212 | 118/124 | 191/217 | 133/133 | 116/127 | 157/168 | 172/216 |
| 135 | C | 136 | 204/212 | 237/247 | 129/163 | 203/203 | 135/137 | 178/192 | 178/178 | 140/160 | 243/243 | 124/159 | 166/176 | 183/185 | 210/210 | 207/212 | 118/124 | 197/199 | 147/188 | 127/131 | 154/157 | 208/214 |
| **136** | **C & M** | **137** | **204/214** | **237/247** | **129/161** | **195/203** | **135/139** | **180/192** | **154/172** | **146/178** | **243/243** | **122/124** | **168/176** | **183/183** | **210/220** | **207/212** | **124/141** | **183/199** | **159/186** | **125/127** | **157/157** | **172/175** |
| **137** | **C & M** | **138** | **204/212** | **237/247** | **138/161** | **203/203** | **135/139** | **182/192** | **194/219** | **140/160** | **254/254** | **122/124** | **166/172** | **183/185** | **210/210** | **212/212** | **118/124** | **197/199** | **184/184** | **127/134** | **154/157** | **208/216** |
| **138** | **C & M** | **139** | **204/214** | **237/247** | **130/161** | **203/203** | **137/137** | **182/192** | **156/156** | **140/160** | **254/254** | **124/152** | **166/176** | **183/183** | **210/220** | **212/212** | **118/121** | **197/217** | **133/147** | **116/119** | **152/168** | **172/216** |
| 139 | C | 140 | 204/214 | 237/247 | 130/161 | 203/203 | 139/139 | 182/192 | 154/154 | 178/182 | 243/243 | 122/124 | 168/168 | 183/183 | 210/210 | 212/212 | 118/127 | 191/217 | 133/133 | 116/119 | 152/168 | 172/212 |
| **140** | **C & M** | **141** | **204/212** | **237/247** | **130/161** | **203/203** | **139/139** | **182/192** | **194/218** | **160/182** | **243/263** | **122/124** | **168/172** | **181/183** | **210/220** | **207/212** | **127/141** | **191/217** | **133/133** | **119/134** | **152/154** | **172/212** |
| **141** | **C & M** | **142** | **204/214** | **237/247** | **130/161** | **203/203** | **139/139** | **182/192** | **154/154** | **140/178** | **243/254** | **122/173** | **168/172** | **183/183** | **210/220** | **212/212** | **118/124** | **191/217** | **133/133** | **116/127** | **157/168** | **172/216** |
| **142** | **C & M** | **143** | **204/214** | **237/247** | **130/161** | **203/203** | **139/139** | **182/192** | **154/154** | **140/178** | **243/254** | **122/173** | **168/172** | **183/183** | **210/220** | **212/212** | **118/124** | **191/217** | **133/133** | **116/127** | **157/168** | **172/218** |
| **143** | **C & M** | **144** | **204/214** | **237/247** | **130/130** | **203/203** | **135/139** | **182/192** | **154/154** | **140/160** | **263/263** | **152/175** | **168/176** | **183/183** | **210/220** | **207/212** | **118/121** | **191/199** | **184/184** | **116/119** | **152/168** | **172/212** |
| 144 | C | 145 | 204/214 | 237/247 | 154/161 | 203/207 | 135/137 | 182/192 | 218/218 | 140/178 | 243/243 | 161/173 | 172/172 | 183/185 | 210/222 | 212/212 | 118/121 | 197/199 | 147/184 | 127/142 | 154/157 | 210/210 |
| **145** | **C & M** | **146** | **204/214** | **237/247** | **130/130** | **203/203** | **139/139** | **182/190** | **154/154** | **140/176** | **243/254** | **124/152** | **166/172** | **183/183** | **210/220** | **212/212** | **118/127** | **191/217** | **133/133** | **116/119** | **152/168** | **208/212** |
| 146 | C | 147 | 204/214 | 237/247 | 130/130 | 203/203 | 139/139 | 182/190 | 154/154 | 140/176 | 243/254 | 124/152 | 166/172 | 183/183 | 210/220 | 212/212 | 118/127 | 191/217 | 133/133 | 116/119 | 152/168 | 208/214 |
| 147 | C | 148 | 204/214 | 237/247 | 130/130 | 203/203 |  | 182/190 |  | 140/176 | 243/254 | 124/152 | 166/172 | 183/183 | 210/220 | 212/212 | 118/127 | 191/207 | 133/133 | 116/119 |  | 208/212 |
| 148 | C | 149 | 204/214 | 237/247 | 130/130 | 203/203 |  | 168/190 |  | 140/176 | 243/254 | 124/152 | 166/172 | 183/183 | 210/220 | 212/212 | 118/127 | 191/217 | 133/133 | 116/119 |  | 208/212 |
| 149 | C | 150 | 204/212 | 243/251 | 130/130 | 203/203 |  | 160/204 |  | 140/160 | 254/254 | 152/173 | 168/183 | 183/185 | 210/210 | 212/212 | 121/141 | 197/217 | 133/147 | 119/134 |  | 210/212 |
| 150 | C | 151 | 204/212 | 237/247 | 129/129 | 191/203 | 134/135 | 192/192 | 218/218 | 146/178 | 243/243 | 124/148 | 168/172 | 181/183 | 206/210 | 212/212 | 127/141 | 197/217 | 133/174 | 127/127 | 157/160 | 172/175 |
| 151 | C | 152 | 204/212 | 237/247 | 130/188 | 191/203 | 129/135 | 192/192 | 194/218 | 140/160 | 254/254 | 124/169 | 168/172 | 181/183 | 210/210 | 207/212 | 127/127 | 191/217 | 133/147 | 114/119 | 152/160 | 175/175 |
| 152 | C | 153 | 204/204 | 237/247 | 159/161 | 191/203 | 137/137 | 192/192 | 194/218 | 146/160 | 243/243 | 124/152 | 174/180 | 183/189 | 206/210 | 212/212 | 141/141 | 199/205 | 133/147 | 127/127 | 157/157 | 172/175 |
| 153 | C | 154 | 204/212 | 237/247 | 138/161 | 203/203 | 139/139 | 192/204 | 154/154 | 160/178 | 254/254 | 122/173 | 166/172 | 183/183 | 210/210 | 212/212 | 121/141 | 191/217 | 133/133 | 127/134 | 154/157 | 208/212 |
| **154** | **C & M** | **155** | **204/214** | **237/247** | **130/161** | **203/203** | **135/139** | **192/204** | **218/218** | **178/178** | **243/243** | **124/141** | **166/176** | **183/183** | **210/220** | **207/212** | **118/121** | **197/199** | **147/184** | **116/119** | **152/168** | **204/208** |
| 155 | C | 156 | 204/212 | 237/247 | 130/161 | 203/203 | 137/137 | 192/204 | 154/154 | 140/178 | 243/243 | 152/173 | 166/176 | 183/185 | 210/220 | 212/212 | 118/127 | 197/217 | 147/184 | 119/134 | 152/154 | 208/216 |
| 156 | C | 157 | 204/212 | 237/247 | 138/161 | 203/203 | 137/137 | 192/204 | 154/154 | 140/176 | 243/243 | 148/173 | 168/172 | 183/185 | 210/210 | 212/212 | 121/141 | 189/217 | 133/157 | 103/119 | 152/154 | 172/175 |
| 157 | C | 158 | 204/214 | 237/247 | 130/130 | 203/203 | 135/137 | 192/204 | 219/219 | 178/178 | 243/243 | 122/173 | 168/172 | 183/185 | 210/220 | 212/212 | 118/127 | 197/199 | 147/184 | 119/134 | 152/154 | 172/216 |
| **158** | **C & M** | **159** | **204/212** | **237/247** | **130/161** | **203/203** | **139/139** | **192/204** | **194/219** | **160/178** | **263/263** | **124/152** | **168/176** | **183/183** | **210/210** | **212/212** | **118/127** | **191/217** | **133/133** | **119/134** | **152/154** | **172/216** |
| 159 | C | 160 | 204/212 | 237/247 | 130/161 | 203/203 | 135/139 | 192/204 | 194/218 | 160/178 | 263/263 | 124/152 | 168/176 | 183/183 | 210/210 | 212/212 | 118/127 | 191/217 | 133/133 | 119/134 | 152/154 | 172/218 |
| **160** | **C & M** | **161** | **204/214** | **237/247** | **138/161** | **203/203** | **135/137** | **192/204** | **154/154** | **140/178** | **243/254** | **124/152** | **168/176** | **183/185** | **210/220** | **207/212** | **121/141** | **197/199** | **147/186** | **119/134** | **152/154** | **208/216** |
| 161 | C | 162 | 204/214 | 237/247 | 138/161 | 203/203 | 135/137 | 192/204 | 154/154 | 140/178 | 243/254 | 124/152 | 168/176 | 183/185 | 210/220 | 207/212 | 121/141 | 197/199 | 147/184 | 119/134 | 152/154 | 208/216 |
| 162 | C | 163 | 204/214 | 237/247 | 138/161 | 203/203 | 135/137 | 192/204 | 154/154 | 140/178 | 243/254 | 124/152 | 168/176 | 183/185 | 210/220 | 207/212 | 121/141 | 197/199 | 147/186 | 119/134 | 152/154 | 208/218 |
| **163** | **C & M** | **164** | **204/214** | **237/249** | **130/130** | **203/203** | **139/139** | **182/190** | **154/154** | **140/176** | **243/254** | **124/152** | **166/172** | **183/183** | **210/220** | **212/212** | **118/127** | **191/217** | **133/133** | **116/119** | **152/168** | **208/212** |
| 164 | C | 165 | 204/212 | 237/253 | 130/130 | 203/203 | 137/137 | 192/204 | 218/218 | 140/160 | 243/243 | 122/175 | 168/176 | 183/183 | 210/220 | 207/212 | 118/121 | 197/217 | 133/147 | 119/134 | 152/168 | 208/216 |
| 165 | C | 166 | 204/212 | 237/251 | 130/161 | 191/203 | 137/137 | 160/170 | 154/154 | 146/178 | 243/243 | 122/124 | 174/176 | 183/189 | 206/210 | 212/212 | 127/141 | 205/217 | 133/147 | 125/127 | 157/157 | 172/212 |
| **166** | **C & M** | **167** | **204/204** | **237/251** | **142/142** | **203/205** | **135/149** | **160/180** | **152/154** | **130/160** | **243/243** | **152/173** | **170/172** | **183/183** | **210/226** | **207/212** | **124/141** | **183/199** | **133/157** | **119/131** | **152/152** | **208/214** |
| **167** | **C & M** | **168** | **204/214** | **237/251** | **138/138** | **203/203** | **137/137** | **160/182** | **219/219** | **140/160** | **243/243** | **122/173** | **166/176** | **183/185** | **210/210** | **212/212** | **121/141** | **197/217** | **133/147** | **127/134** | **154/157** | **172/212** |
| 168 | C | 169 | 204/214 | 237/251 | 130/161 | 203/203 | 137/137 | 160/182 | 154/154 | 140/160 | 254/254 | 122/124 | 168/172 | 183/185 | 210/210 | 207/212 | 118/127 | 197/217 | 133/133 | 116/119 | 152/168 | 172/216 |
| 169 | C | 170 | 204/214 | 237/251 | 130/161 | 203/203 | 137/137 | 160/182 | 152/152 | 140/160 | 254/254 | 122/124 | 168/172 | 183/185 | 210/210 | 207/212 | 118/127 | 197/217 | 133/133 | 116/119 | 152/168 | 172/212 |
| 170 | C | 171 | 204/214 | 237/251 | 130/130 | 203/203 | 135/137 | 160/182 | 154/154 | 140/178 | 243/254 | 124/152 | 168/176 | 183/185 | 210/220 | 207/212 | 118/121 | 197/217 | 147/184 | 119/134 | 152/154 | 172/216 |
| 171 | C | 172 | 204/214 | 237/251 | 130/130 | 203/203 | 135/139 | 160/182 | 218/218 | 160/178 | 263/263 | 124/152 | 168/176 | 183/183 | 210/220 | 212/212 | 121/141 | 191/199 | 184/184 | 127/134 | 154/157 | 208/216 |
| **172** | **C & M** | **173** | **204/204** | **237/251** | **129/161** | **203/205** | **134/135** | **160/202** | **218/218** | **160/178** | **243/263** | **148/173** | **168/172** | **181/183** | **206/210** | **212/212** | **118/121** | **189/217** | **133/171** | **119/125** | **152/152** | **172/175** |
| 173 | C | 174 | 204/204 | 237/251 | 129/161 | 203/203 |  | 160/202 |  | 140/160 | 243/243 | 152/173 | 168/172 | 181/183 | 206/210 | 212/212 | 121/141 | 189/217 | 133/171 | 119/125 |  | 172/212 |
| **174** | **C & M** | **175** | **204/212** | **237/251** | **130/161** | **203/203** | **139/139** | **160/204** | **154/154** | **160/178** | **263/263** | **122/173** | **166/172** | **183/183** | **210/220** | **207/212** | **118/121** | **191/217** | **133/133** | **116/119** | **152/168** | **172/216** |
| **175** | **C & M** | **176** | **204/214** | **237/251** | **138/138** | **203/203** | **137/139** | **160/204** | **154/154** | **160/178** | **243/243** | **122/124** | **166/176** | **183/185** | **210/210** | **207/212** | **127/141** | **191/199** | **178/184** | **116/119** | **152/168** | **172/214** |
| **176** | **C & M** | **177** | **204/214** | **237/251** | **138/161** | **203/203** | **139/139** | **160/204** | **154/154** | **140/178** | **243/254** | **122/124** | **166/176** | **183/183** | **210/220** | **207/212** | **121/141** | **191/219** | **133/133** | **119/134** | **152/154** | **208/216** |
| 177 | C | 178 | 204/212 | 237/251 | 186/186 | 203/203 | 137/137 | 160/204 | 218/244 | 146/160 | 243/243 | 148/173 | 168/168 | 183/189 | 210/220 | 207/212 | 127/141 | 205/217 | 133/147 | 116/119 | 152/168 | 172/212 |
| 178 | C | 179 | 204/214 | 237/251 | 130/161 | 203/203 | 135/137 | 160/204 | 154/154 | 140/178 | 243/254 | 122/173 | 168/168 | 183/185 | 210/210 | 207/212 | 118/127 | 197/199 | 147/184 | 127/134 | 154/157 | 172/214 |
| **179** | **C & M** | **180** | **204/214** | **237/251** | **130/161** | **203/203** | **135/137** | **160/204** | **154/154** | **140/174** | **243/254** | **122/173** | **168/172** | **183/185** | **210/210** | **207/212** | **118/127** | **197/199** | **147/184** | **127/134** | **154/157** | **172/214** |
| **180** | **C & M** | **181** | **204/214** | **237/251** | **130/161** | **203/203** | **134/137** | **160/204** | **154/154** | **140/178** | **243/254** | **122/173** | **168/172** | **183/185** | **210/210** | **207/212** | **118/127** | **197/199** | **147/184** | **127/134** | **154/157** | **172/214** |
| 181 | C | 182 | 204/212 | 237/251 | 130/130 | 203/203 | 137/137 | 160/204 | 218/218 | 140/178 | 243/254 | 124/152 | 168/172 | 183/185 | 210/220 | 212/212 | 121/141 | 197/217 | 133/147 | 116/119 | 152/168 | 172/216 |
| 182 | C | 183 | 204/212 | 237/251 | 138/138 | 203/203 | 137/137 | 160/204 | 154/154 | 140/178 | 243/254 | 152/177 | 168/176 | 183/185 | 210/220 | 212/212 | 118/121 | 197/217 | 133/147 | 119/134 | 154/157 | 172/216 |
| 183 | C | 184 | 204/214 | 237/251 | 138/161 | 203/203 | 135/137 | 160/204 | 154/154 | 140/160 | 263/263 | 122/124 | 168/176 | 183/185 | 210/210 | 212/212 | 118/127 | 197/217 | 184/184 | 116/119 | 152/168 | 208/216 |
| 184 | C | 185 | 204/214 | 237/251 | 130/161 | 203/203 | 137/137 | 160/204 | 218/218 | 140/160 | 243/263 | 152/173 | 168/176 | 183/185 | 210/220 | 207/212 | 118/121 | 197/217 | 133/147 | 119/134 | 152/154 | 214/216 |
| **185** | **C & M** | **186** | **204/229** | **237/251** | **136/161** | **203/211** | **123/139** | **160/190** | **154/154** | **126/140** | **254/254** | **122/163** | **168/176** | **183/183** | **210/210** | **212/225** | **124/127** | **183/191** | **157/157** | **119/134** | **152/154** | **172/208** |
| **186** | **C & M** | **187** | **212/214** | **237/251** | **162/188** | **199/203** | **123/135** | **170/174** | **156/156** | **136/160** | **254/254** | **144/148** | **168/170** | **183/183** | **210/220** | **212/230** | **118/124** | **189/199** | **165/184** | **116/131** | **168/168** | **172/179** |
| 187 | C | 188 | 204/214 | 237/251 | 130/130 | 203/203 | 137/137 | 182/192 | 194/218 | 160/178 | 263/263 | 152/173 | 166/172 | 183/185 | 210/210 | 212/212 | 121/141 | 197/217 | 133/147 | 116/127 | 157/168 | 172/212 |
| 188 | C | 189 | 204/214 | 237/251 | 130/130 | 203/203 | 135/137 | 182/192 | 154/154 | 178/178 | 243/243 | 152/173 | 166/172 | 183/185 | 210/220 | 212/212 | 118/121 | 197/199 | 147/190 | 127/134 | 154/157 | 208/212 |
| **189** | **C & M** | **190** | **204/214** | **237/251** | **130/130** | **203/203** | **135/137** | **182/192** | **154/154** | **178/178** | **243/243** | **152/173** | **166/172** | **183/185** | **210/220** | **212/212** | **118/121** | **197/199** | **147/184** | **127/134** | **154/157** | **208/212** |
| **190** | **C & M** | **191** | **204/214** | **237/251** | **138/161** | **203/203** | **137/137** | **182/192** | **154/154** | **160/178** | **243/243** | **122/175** | **166/172** | **183/185** | **210/210** | **212/212** | **118/127** | **197/217** | **133/147** | **127/134** | **154/157** | **208/216** |
| 191 | C | 192 | 204/214 | 237/251 | 130/130 | 203/203 | 135/139 | 182/192 | 218/218 | 160/178 | 243/243 | 122/177 | 166/176 | 183/183 | 210/220 | 207/212 | 121/141 | 191/199 | 184/184 | 127/134 | 154/157 | 172/212 |
| 192 | C | 193 | 204/212 | 237/251 | 130/130 | 203/203 | 135/137 | 182/192 | 218/218 | 176/176 | 243/243 | 122/124 | 168/172 | 183/183 | 210/210 | 207/212 | 121/141 | 191/199 | 147/184 | 119/131 | 152/154 | 172/212 |
| 193 | C | 194 | 204/214 | 237/251 | 130/161 | 203/203 | 135/139 | 182/192 | 218/218 | 178/178 | 243/243 | 152/175 | 168/172 | 183/183 | 210/220 | 212/212 | 118/121 | 191/199 | 184/184 | 116/127 | 157/168 | 208/216 |
| 194 | C | 195 | 204/229 | 237/251 | 136/161 | 203/211 | 123/139 | 182/192 | 154/154 | 126/140 | 254/254 | 152/173 | 168/176 | 183/183 | 210/210 | 212/225 | 124/127 | 183/191 | 157/157 | 119/134 | 152/154 | 172/212 |
| **195** | **C & M** | **196** | **204/214** | **237/251** | **130/161** | **203/203** | **137/137** | **182/192** | **218/218** | **140/160** | **243/243** | **122/124** | **168/176** | **183/185** | **210/220** | **207/212** | **127/141** | **197/217** | **133/147** | **119/134** | **152/154** | **172/216** |
| 196 | C | 197 | 204/212 | 237/251 | 182/182 | 203/203 | 135/135 | 182/204 | 154/154 | 160/178 | 243/263 | 122/148 | 172/176 | 183/189 | 206/210 | 207/212 | 121/141 | 217/217 | 133/133 | 116/119 | 152/168 | 172/212 |
| 197 | C | 198 | 204/212 | 237/251 | 186/186 | 203/203 | 135/135 | 192/200 | 194/218 | 160/178 | 243/243 | 148/173 | 172/174 | 183/185 | 210/210 | 207/212 | 127/144 | 197/199 | 171/184 | 116/119 | 152/168 | 172/175 |
| 198 | C | 199 | 204/212 | 237/251 | 130/161 | 203/203 |  | 160/182 |  | 140/176 | 243/254 | 124/152 | 168/172 | 183/183 | 210/220 | 212/212 | 127/141 | 191/199 | 184/184 | 127/134 |  | 208/210 |
| **199** | **C & M** | **200** | **204/212** | **237/251** | **130/130** | **203/207** | **137/137** | **192/202** | **154/154** | **166/178** | **243/263** | **122/124** | **172/176** | **183/185** | **206/210** | **212/212** | **124/141** | **197/217** | **133/147** | **114/127** | **157/160** | **172/216** |
| 200 | C | 201 | 204/212 | 237/251 | 130/161 | 203/203 | 135/137 | 192/204 | 154/154 | 140/160 | 243/254 | 122/124 | 166/172 | 183/185 | 210/210 | 212/212 | 127/141 | 197/199 | 147/184 | 116/119 | 152/168 | 172/212 |
| 201 | C | 202 | 204/212 | 237/251 | 138/138 | 203/203 |  | 192/204 |  | 178/178 | 243/254 | 124/152 | 166/176 | 183/183 | 210/210 | 212/212 | 118/121 | 191/199 | 147/184 | 119/134 |  | 172/216 |
| 202 | C | 203 | 204/212 | 237/251 | 130/130 | 203/203 | 135/139 | 192/204 | 154/154 | 140/160 | 263/263 | 152/173 | 168/172 | 183/183 | 210/210 | 207/212 | 118/121 | 189/219 | 184/184 | 116/119 | 152/168 | 210/216 |
| **203** | **C & M** | **204** | **204/212** | **237/251** | **130/130** | **203/203** | **135/139** | **192/204** | **154/154** | **140/160** | **263/263** | **152/173** | **168/172** | **183/183** | **210/210** | **207/212** | **118/121** | **191/217** | **184/184** | **116/119** | **152/168** | **210/216** |
| 204 | C | 205 | 204/268 | 229/241 | 129/130 | 195/203 |  | 180/204 |  | 130/178 | 243/243 | 148/154 | 172/174 | 183/189 | 206/210 | 212/221 | 121/141 | 183/197 | 159/171 | 114/125 |  | 175/212 |
| 205 | C | 206 | 204/212 | 237/251 | 130/130 | 203/203 | 137/137 | 192/202 | 154/154 | 140/160 | 243/254 | 152/173 | 168/176 | 183/185 | 210/220 | 212/212 | 118/127 | 197/217 | 133/147 | 127/134 | 152/154 | 172/212 |
| **206** | **C & M** | **208** | **204/212** | **237/251** | **130/130** | **203/203** | **137/137** | **192/204** | **154/154** | **140/160** | **243/254** | **152/173** | **168/176** | **183/185** | **210/220** | **212/212** | **118/127** | **197/217** | **133/147** | **127/134** | **152/154** | **172/210** |
| 207 | C | 209 | 204/212 | 237/251 | 138/161 | 203/203 | 135/139 | 192/204 | 154/154 | 140/164 | 254/254 | 122/173 | 168/176 | 183/185 | 210/220 | 207/212 | 121/141 | 197/199 | 184/184 | 116/119 | 152/168 | 172/216 |
| **208** | **C & M** | **210** | **204/212** | **237/251** | **130/130** | **203/203** | **137/137** | **192/204** | **216/216** | **140/160** | **243/243** | **122/175** | **168/176** | **183/183** | **210/220** | **207/212** | **118/121** | **197/217** | **133/147** | **119/134** | **152/168** | **208/216** |
| 209 | C | 211 | 212/212 | 237/251 | 161/186 | 191/203 | 135/135 | 200/204 | 154/154 | 130/178 | 263/263 | 143/148 | 172/191 | 183/189 | 210/220 | 207/207 | 121/141 | 191/197 | 171/171 | 131/134 | 152/157 | 172/210 |
| **210** | **C & M** | **212** | **204/229** | **237/251** | **136/161** | **203/211** | **123/135** | **190/192** | **156/156** | **126/178** | **243/243** | **163/179** | **168/176** | **183/183** | **210/222** | **212/225** | **118/124** | **183/199** | **157/184** | **119/131** | **152/152** | **170/172** |
| **211** | **C & M** | **213** | **212/212** | **241/241** | **161/186** | **191/203** | **129/135** | **164/178** | **138/138** | **126/160** | **243/263** | **124/143** | **166/174** | **181/185** | **206/210** | **207/207** | **124/127** | **189/199** | **133/171** | **116/125** | **152/168** | **170/181** |
| 212 | C | 214 | 204/212 | 241/241 | 130/186 | 199/203 | 129/135 | 174/192 | 152/152 | 146/152 | 243/243 | 122/173 | 170/170 | 183/185 | 210/210 | 212/212 | 124/127 | 191/199 | 147/184 | 116/116 | 168/168 | 175/175 |
| 213 | C | 215 | 204/212 | 241/243 | 129/186 | 191/205 | 129/134 | 170/184 | 238/238 | 140/146 | 243/243 | 122/148 | 166/168 | 185/193 | 206/206 | 212/212 | 124/127 | 191/197 | 147/174 | 125/127 | 160/160 | 175/214 |
| **214** | **C & M** | **216** | **212/214** | **241/243** | **138/186** | **199/203** | **137/139** | **170/184** | **162/162** | **140/146** | **243/243** | **122/152** | **168/180** | **183/185** | **206/220** | **212/212** | **121/141** | **191/197** | **147/147** | **125/134** | **154/154** | **175/187** |
| 215 | C | 217 | 204/212 | 241/243 | 130/138 | 199/205 | 129/134 | 170/184 | 162/162 | 140/178 | 254/254 | 122/152 | 174/180 | 183/189 | 210/220 | 210/212 | 124/127 | 191/205 | 171/171 | 116/134 | 154/168 | 175/187 |
| 216 | C | 218 | 204/204 | 241/243 | 154/190 | 203/211 | 123/135 | 174/192 | 168/168 | 146/178 | 243/243 | 152/175 | 168/172 | 183/183 | 210/220 | 210/212 | 124/141 | 191/199 | 157/190 | 116/134 | 154/168 | 172/216 |
| **217** | **C & M** | **219** | **212/232** | **241/243** | **192/192** | **195/203** | **125/161** | **184/200** | **140/140** | **126/150** | **243/263** | **122/136** | **162/176** | **183/183** | **210/220** | **207/212** | **124/127** | **183/191** | **171/171** | **114/125** | **160/160** | **175/216** |
| **218** | **C & M** | **220** | **212/214** | **241/243** | **163/186** | **195/203** | **129/134** | **184/204** | **152/164** | **140/178** | **243/243** | **144/152** | **166/176** | **189/193** | **210/216** | **210/212** | **121/124** | **191/197** | **147/171** | **114/125** | **160/160** | **187/216** |
| **219** | **C & M** | **221** | **212/212** | **241/243** | **186/194** | **195/203** | **137/149** | **184/206** | **146/146** | **146/146** | **243/243** | **148/175** | **168/180** | **185/185** | **210/220** | **212/212** | **124/127** | **183/205** | **147/159** | **103/119** | **152/154** | **172/175** |
| 220 | C | 222 | 212/212 | 241/243 | 182/196 | 195/203 |  | 184/210 |  | 146/146 | 243/243 | 148/175 | 168/183 |  | 210/220 | 212/212 | 124/127 | 183/205 | 147/159 | 103/119 |  | 172/175 |
| 221 | C | 223 | 204/212 | 241/243 | 159/186 | 191/205 | 129/137 | 192/204 | 152/152 | 146/178 | 243/243 | 148/152 | 166/174 | 181/189 | 206/210 | 212/212 | 124/127 | 189/205 | 147/171 | 114/116 | 160/168 | 175/214 |
| 222 | C | 224 | 204/212 | 241/243 | 159/186 | 191/205 | 129/137 | 192/204 | 152/152 | 146/178 | 243/243 | 148/152 | 166/174 | 181/189 | 206/210 | 212/212 | 124/127 | 189/205 | 147/171 | 114/116 | 160/168 | 175/216 |
| 223 | C | 225 | 212/214 | 241/245 | 186/186 | 199/203 | 129/137 | 184/206 | 146/146 | 126/146 | 243/243 | 122/175 | 166/180 | 181/185 | 220/220 | 212/212 | 124/124 | 189/205 | 147/171 | 119/125 | 152/152 | 172/212 |
| **224** | **C & M** | **226** | **204/212** | **241/247** | **130/161** | **203/203** | **137/137** | **160/192** | **218/240** | **146/160** | **243/243** | **148/173** | **168/176** | **183/189** | **206/210** | **207/212** | **118/141** | **205/217** | **133/147** | **119/125** | **152/168** | **172/175** |
| 225 | C | 227 | 204/204 | 241/247 | 159/163 | 203/205 | 129/129 | 170/192 | 154/154 | 146/160 | 243/243 | 124/148 | 172/172 | 181/183 | 206/210 | 212/212 | 124/141 | 189/217 | 133/171 | 119/127 | 152/160 | 172/175 |
| 226 | C | 228 | 204/204 | 241/247 | 130/130 | 203/203 | 135/137 | 170/192 | 219/240 | 146/178 | 243/243 | 148/173 | 172/174 | 183/185 | 210/220 | 207/212 | 118/127 | 197/199 | 147/184 | 119/125 | 152/152 | 175/208 |
| **227** | **C & M** | **229** | **204/212** | **241/247** | **130/130** | **203/203** | **137/137** | **192/202** | **154/154** | **140/178** | **243/243** | **122/173** | **166/172** | **183/189** | **210/216** | **212/212** | **127/141** | **205/217** | **133/147** | **114/119** | **152/160** | **175/210** |
| 228 | C | 230 | 204/212 | 241/247 | 130/130 | 203/203 | 137/137 | 192/202 | 154/154 | 140/178 | 243/243 | 122/173 | 166/172 | 183/189 | 210/216 | 212/212 | 127/141 | 205/217 | 133/147 | 114/119 | 152/160 | 175/212 |
| **229** | **C & M** | **231** | **204/212** | **241/247** | **130/130** | **203/203** | **137/137** | **192/204** | **154/154** | **140/178** | **243/243** | **122/173** | **166/172** | **183/189** | **210/216** | **212/212** | **127/141** | **205/217** | **133/147** | **114/119** | **152/160** | **175/210** |
| 230 | C | 232 | 204/212 | 241/247 | 186/186 | 191/203 | 135/137 | 192/204 | 154/154 | 160/178 | 243/243 | 124/148 | 168/176 | 183/189 | 210/210 | 207/212 | 118/127 | 199/205 | 147/184 | 125/127 | 157/157 | 172/214 |
| 231 | C | 233 | 204/214 | 241/251 | 186/186 | 203/203 | 135/135 | 160/170 | 162/162 | 140/178 | 243/254 | 148/173 | 168/172 | 183/183 | 210/220 | 207/212 | 121/141 | 183/199 | 157/184 | 116/119 | 152/168 | 172/177 |
| 232 | C | 234 | 204/212 | 241/251 | 130/130 | 191/203 | 134/135 | 160/170 | 194/218 | 146/178 | 243/243 | 122/124 | 168/176 | 183/185 | 210/210 | 207/212 | 118/141 | 197/217 | 133/171 | 116/119 | 152/168 | 172/214 |
| **233** | **C & M** | **235** | **204/223** | **241/251** | **186/186** | **195/205** | **135/135** | **160/182** | **154/154** | **160/178** | **243/243** | **124/173** | **166/172** | **183/183** | **206/210** | **212/246** | **124/127** | **199/199** | **174/186** | **114/119** | **160/160** | **208/216** |
| **234** | **C & M** | **236** | **204/204** | **241/251** | **130/130** | **203/203** | **129/135** | **160/202** | **154/154** | **160/178** | **263/263** | **122/124** | **166/176** | **183/193** | **210/216** | **212/212** | **118/124** | **191/217** | **133/147** | **119/125** | **152/152** | **172/214** |
| 235 | C | 237 | 204/268 | 241/251 | 129/129 | 203/203 | 135/135 | 160/204 | 154/176 | 140/174 | 254/254 | 152/171 | 172/174 | 183/189 | 210/210 | 212/221 | 121/141 | 197/199 | 171/184 | 125/134 | 154/154 | 210/214 |
| **236** | **C & M** | **238** | **204/258** | **241/251** | **129/130** | **195/203** | **135/135** | **160/204** | **154/176** | **130/178** | **254/254** | **148/173** | **172/172** | **183/189** | **206/210** | **212/221** | **121/141** | **197/199** | **171/184** | **114/134** | **154/160** | **210/214** |
| 237 | C | 239 | 204/258 | 241/251 | 129/130 | 195/203 | 135/135 | 160/208 | 154/176 | 130/178 | 254/254 | 148/173 | 172/172 | 183/189 | 206/210 | 212/221 | 121/141 | 197/199 | 171/188 | 114/134 | 154/160 | 210/214 |
| 238 | C | 240 | 204/212 | 241/251 | 130/130 | 191/203 | 134/135 | 170/192 | 194/218 | 146/178 | 243/243 | 124/148 | 168/174 | 183/189 | 210/210 | 207/212 | 127/141 | 197/199 | 133/171 | 116/119 | 152/168 | 172/214 |
| **239** | **C & M** | **241** | **212/212** | **241/251** | **142/159** | **195/205** | **135/149** | **180/204** | **154/154** | **146/160** | **243/243** | **122/173** | **166/170** | **183/183** | **210/220** | **212/212** | **121/124** | **183/217** | **133/157** | **103/119** | **152/154** | **208/216** |
| 240 | C | 242 | 204/212 | 241/251 | 159/159 | 203/203 | 134/135 | 192/192 | 154/154 | 140/160 | 243/243 | 122/173 | 166/172 | 183/193 | 210/210 | 212/212 | 127/141 | 191/217 | 133/171 | 116/127 | 157/168 | 206/216 |
| 241 | C | 243 | 204/204 | 237/243 | 159/186 | 191/203 |  | 170/202 |  | 140/146 | 243/243 | 122/152 | 172/174 | 189/193 | 206/216 | 212/212 | 121/127 | 191/205 | 147/147 | 116/127 |  | 214/216 |
| 242 | C | 244 | 204/204 | 241/251 | 129/130 | 203/203 | 129/137 | 192/204 | 218/226 | 146/178 | 243/243 | 144/152 | 172/174 | 185/189 | 210/210 | 212/212 | 118/121 | 197/205 | 157/171 | 127/129 | 157/160 | 175/175 |
| 243 | C | 245 | 204/212 | 243/247 | 129/161 | 203/205 | 129/134 | 174/184 | 152/152 | 148/178 | 243/243 | 124/152 | 172/172 | 183/193 | 210/216 | 212/212 | 124/124 | 191/197 | 147/174 | 114/119 | 152/160 | 175/216 |
| 244 | C | 246 | 204/214 | 243/247 | 130/161 | 203/203 | 135/139 | 182/192 | 154/154 | 178/178 | 243/254 | 122/124 | 166/176 | 183/183 | 210/210 | 212/212 | 127/141 | 191/217 | 184/184 | 119/134 | 152/154 | 172/212 |
| 245 | C | 247 | 204/214 | 243/247 | 130/130 | 197/203 | 137/137 | 182/192 | 218/218 | 160/178 | 243/263 | 122/165 | 168/176 | 183/185 | 210/220 | 207/212 | 118/127 | 197/217 | 133/147 | 119/134 | 152/154 | 210/210 |
| **246** | **C & M** | **248** | **204/212** | **243/251** | **130/130** | **203/203** | **139/139** | **182/192** | **156/156** | **160/178** | **243/243** | **124/152** | **168/172** | **183/183** | **210/220** | **212/212** | **118/121** | **191/217** | **133/133** | **119/134** | **154/157** | **172/212** |
| 247 | C | 249 | 204/214 | 243/247 | 130/161 | 203/203 | 139/139 | 192/204 | 154/154 | 160/178 | 243/263 | 152/175 | 168/176 | 183/183 | 210/220 | 212/212 | 118/127 | 191/217 | 133/133 | 119/134 | 152/154 | 172/216 |
| **248** | **C & M** | **250** | **204/214** | **243/251** | **130/130** | **203/203** | **135/137** | **160/182** | **154/154** | **160/178** | **254/254** | **122/124** | **166/172** | **183/185** | **210/210** | **207/212** | **118/121** | **197/197** | **147/184** | **116/119** | **152/168** | **208/212** |
| 249 | C | 251 | 204/212 | 243/251 | 130/161 | 203/203 | 137/137 | 192/204 | 154/154 | 140/178 | 243/254 | 124/152 | 168/176 | 183/185 | 210/220 | 207/212 | 121/141 | 197/217 | 133/147 | 116/119 | 152/168 | 208/212 |
| 250 | C | 252 | 204/204 | 243/243 |  | 199/203 | 134/135 | 160/206 | 152/152 | 146/146 | 243/243 | 148/173 | 168/172 | 181/181 | 216/216 | 207/212 | 118/141 | 199/203 | 149/184 | 119/127 | 152/157 | 208/218 |
| 251 | C | 253 | 204/204 | 243/245 | 138/159 | 195/203 | 129/151 | 170/192 | 172/172 | 134/178 | 243/254 | 152/175 | 166/172 | 183/189 | 206/220 | 212/225 | 121/121 | 191/193 | 133/174 | 119/125 | 152/152 | 172/175 |
| 252 | C | 254 | 204/212 | 243/243 | 130/163 | 191/203 | 137/163 | 184/192 | 146/146 | 126/146 | 243/243 | 122/124 | 162/166 | 181/181 | 220/220 | 207/210 | 121/141 | 189/191 | 147/157 | 125/125 | 152/152 | 214/214 |
| 253 | C | 255 | 204/212 | 243/247 | 130/130 | 203/203 | 135/137 | 160/182 | 218/218 | 140/160 | 254/254 | 152/173 | 166/172 | 183/185 | 210/220 | 212/212 | 121/141 | 197/199 | 147/184 | 116/119 | 152/168 | 172/212 |
| **254** | **C & M** | **256** | **204/214** | **243/247** | **130/161** | **203/203** | **137/137** | **160/182** | **154/154** | **140/160** | **243/254** | **122/173** | **166/172** | **183/185** | **210/210** | **212/212** | **127/141** | **197/217** | **133/147** | **119/134** | **152/154** | **212/212** |
| 255 | C | 257 | 204/214 | 243/247 | 130/161 | 203/203 | 137/137 | 160/182 | 154/154 | 140/178 | 243/254 | 152/173 | 168/172 | 183/185 | 210/220 | 212/212 | 124/141 | 197/217 | 133/147 | 119/134 | 152/154 | 210/212 |
| 256 | C | 258 | 204/214 | 243/247 | 130/130 | 203/203 | 135/137 | 160/182 | 154/154 | 160/174 | 263/263 | 122/124 | 168/176 | 183/185 | 210/220 | 207/212 | 127/141 | 197/217 | 133/147 | 119/134 | 152/154 | 208/212 |
| 257 | C | 259 | 204/204 | 243/247 | 129/186 | 191/203 | 135/139 | 160/182 | 152/152 | 140/146 | 243/243 | 124/152 | 168/176 | 185/193 | 216/216 | 212/212 | 121/127 | 191/197 | 147/171 | 116/127 | 157/168 | 212/212 |
| 258 | C | 260 | 204/214 | 243/247 | 138/138 | 199/203 | 135/139 | 160/198 | 146/154 | 140/178 | 243/254 | 124/144 | 164/170 | 183/183 | 210/210 | 207/207 | 121/141 | 191/217 | 133/155 | 119/131 | 152/154 | 172/177 |
| **259** | **C & M** | **261** | **204/214** | **243/247** | **138/138** | **199/203** | **135/139** | **160/204** | **146/154** | **140/178** | **243/254** | **124/144** | **164/172** | **183/183** | **210/210** | **207/207** | **121/141** | **191/217** | **133/155** | **119/131** | **152/154** | **172/177** |
| 260 | C | 262 | 204/212 | 243/247 | 130/161 | 203/203 | 135/139 | 160/204 | 154/154 | 140/160 | 243/263 | 122/173 | 166/176 | 183/183 | 210/220 | 212/212 | 118/127 | 191/217 | 133/133 | 127/134 | 154/157 | 172/212 |
| **261** | **C & M** | **263** | **204/212** | **243/247** | **130/130** | **203/203** | **135/139** | **160/204** | **154/154** | **160/172** | **243/243** | **124/152** | **168/176** | **183/183** | **210/210** | **207/212** | **118/127** | **191/217** | **184/184** | **116/127** | **157/168** | **208/216** |
| 262 | C | 264 | 204/204 | 243/247 | 130/159 | 203/207 |  | 160/206 |  | 146/156 | 243/243 | 122/218 | 166/172 | 181/183 | 210/216 | 212/212 | 121/141 | 189/197 | 137/174 | 114/119 |  | 208/216 |
| **263** | **C & M** | **265** | **204/214** | **243/247** | **130/161** | **203/203** | **137/137** | **160/206** | **194/218** | **140/160** | **243/254** | **124/152** | **168/172** | **183/185** | **210/210** | **207/212** | **121/141** | **197/199** | **133/133** | **119/134** | **152/154** | **216/216** |
| 264 | C | 266 | 204/212 | 243/247 | 138/161 | 203/203 | 137/137 | 160/208 | 154/154 | 140/174 | 243/254 | 152/173 | 166/172 | 183/185 | 210/222 | 207/212 | 121/141 | 197/217 | 133/147 | 119/134 | 152/154 | 172/216 |
| 265 | C | 267 | 204/212 | 243/247 | 129/190 | 203/205 | 129/137 | 170/192 | 218/218 | 146/178 | 263/263 | 152/173 | 166/168 | 189/193 | 206/210 | 212/212 | 124/141 | 191/205 | 147/147 | 127/127 | 157/160 | 175/212 |
| 266 | C | 268 | 204/212 | 243/247 | 129/186 | 203/205 | 129/137 | 170/192 | 218/218 | 146/178 | 263/263 | 152/173 | 166/168 | 189/193 | 206/210 | 212/212 | 124/141 | 191/205 | 147/147 | 127/127 | 157/160 | 212/212 |
| 267 | C | 269 | 204/212 | 243/247 | 129/129 | 203/205 | 129/137 | 170/192 | 218/218 | 146/178 | 243/243 | 152/173 | 166/168 | 189/193 | 206/210 | 212/212 | 124/141 | 191/205 | 147/147 | 127/127 | 157/160 | 212/212 |
| 268 | C | 270 | 204/204 | 247/251 | 161/161 | 203/203 |  | 182/192 |  | 160/178 | 243/243 | 124/173 | 172/176 | 183/183 | 210/210 | 207/212 | 118/141 | 199/217 | 133/184 | 119/127 |  | 172/212 |
| **269** | **C & M** | **271** | **204/212** | **243/247** | **159/159** | **203/203** | **129/134** | **170/192** | **152/152** | **146/160** | **243/243** | **122/124** | **172/172** | **181/183** | **206/210** | **207/212** | **121/124** | **189/197** | **147/171** | **114/119** | **160/168** | **212/214** |
| **270** | **C & M** | **272** | **204/212** | **243/247** | **159/159** | **203/203** | **129/134** | **170/192** | **152/152** | **146/160** | **243/243** | **122/124** | **172/172** | **181/183** | **206/210** | **207/212** | **121/124** | **189/197** | **147/171** | **114/119** | **160/168** | **210/216** |
| 271 | C | 273 | 204/212 | 243/247 | 159/159 | 203/203 | 129/134 | 170/192 | 152/152 | 146/160 | 243/243 | 122/124 | 172/176 | 181/183 | 206/210 | 207/212 | 121/124 | 189/197 | 147/171 | 114/119 | 160/168 | 210/216 |
| **272** | **C & M** | **274** | **204/214** | **243/251** | **138/138** | **203/203** | **135/137** | **160/204** | **154/154** | **160/174** | **263/263** | **122/173** | **166/176** | **183/183** | **210/220** | **207/212** | **127/141** | **191/199** | **147/184** | **116/119** | **152/168** | **208/216** |
| 273 | C | 275 | 204/212 | 243/247 | 159/159 | 203/203 | 129/129 | 170/206 | 218/228 | 156/160 | 243/243 | 124/226 | 172/174 | 181/183 | 210/210 | 207/212 | 121/141 | 189/189 | 147/171 | 119/119 | 152/168 | 216/216 |
| 274 | C | 276 | 204/214 | 237/247 | 130/161 | 203/203 | 139/139 | 182/192 | 154/154 | 140/178 | 243/254 | 122/177 | 168/172 | 183/183 | 210/220 | 212/212 | 118/124 | 191/217 | 133/133 | 116/127 | 157/168 | 172/216 |
| 275 | C | 277 | 204/212 | 243/247 | 152/159 | 197/203 | 129/141 | 174/184 | 216/216 | 140/182 | 243/243 | 122/154 | 172/185 | 181/183 | 206/210 | 212/212 | 124/147 | 189/199 | 141/171 | 116/127 | 152/160 | 169/216 |
| **276** | **C & M** | **278** | **204/212** | **243/247** | **130/130** | **203/203** | **137/137** | **160/182** | **154/154** | **160/178** | **243/243** | **122/173** | **168/176** | **183/185** | **210/220** | **207/212** | **127/141** | **197/217** | **133/147** | **119/134** | **152/154** | **208/212** |
| 277 | C | 279 | 204/204 | 243/247 | 152/159 | 205/207 | 129/141 | 174/196 | 152/152 | 140/178 | 243/243 | 152/154 | 166/185 | 183/193 | 216/216 | 212/212 | 124/147 | 189/199 | 141/171 | 114/116 | 152/160 | 169/175 |
| 278 | C | 280 | 204/212 | 243/247 | 129/130 | 203/205 | 129/135 | 174/202 | 226/226 | 146/178 | 243/243 | 124/152 | 166/189 | 183/193 | 206/210 | 212/212 | 124/141 | 191/199 | 147/184 | 119/127 | 152/160 | 175/210 |
| 279 | C | 281 | 204/204 | 243/247 | 159/167 | 203/205 | 129/135 | 174/204 | 152/152 | 140/180 | 243/243 | 124/152 | 172/172 | 183/193 | 210/216 | 212/212 | 121/124 | 191/199 | 147/184 | 114/119 | 152/160 | 210/216 |
| 280 | C | 282 | 204/212 | 243/247 | 130/161 | 203/203 | 135/137 | 182/192 | 154/154 | 140/160 | 254/254 | 152/173 | 166/172 | 183/183 | 210/220 | 212/212 | 118/127 | 191/199 | 147/184 | 127/134 | 154/157 | 206/212 |
| **281** | **C & M** | **283** | **204/212** | **243/247** | **130/161** | **203/203** | **135/137** | **182/192** | **218/218** | **178/178** | **243/243** | **124/152** | **166/172** | **183/185** | **210/220** | **207/212** | **118/127** | **197/217** | **147/184** | **119/134** | **152/154** | **208/212** |
| **282** | **C & M** | **284** | **204/214** | **243/247** | **130/130** | **203/203** | **137/137** | **182/192** | **154/154** | **140/178** | **243/254** | **124/152** | **168/172** | **183/185** | **210/210** | **207/212** | **118/121** | **197/217** | **133/147** | **116/119** | **152/168** | **172/212** |
| 283 | C | 285 | 204/212 | 229/243 | 129/159 | 203/205 | 129/129 | 192/202 | 152/152 | 140/146 | 243/243 | 122/152 | 166/172 | 181/193 | 206/216 | 212/212 | 121/124 | 189/191 | 147/178 | 114/127 | 160/160 | 175/216 |
| **284** | **C & M** | **286** | **204/204** | **243/247** | **130/130** | **203/203** | **139/139** | **182/192** | **194/218** | **146/160** | **243/243** | **124/173** | **168/172** | **183/185** | **210/210** | **207/212** | **118/124** | **191/217** | **133/133** | **114/119** | **152/160** | **172/216** |
| **285** | **C & M** | **287** | **204/212** | **243/247** | **130/165** | **203/203** | **137/137** | **182/192** | **154/154** | **140/178** | **243/254** | **122/124** | **168/176** | **183/185** | **210/210** | **212/212** | **121/141** | **197/217** | **133/147** | **119/134** | **152/154** | **175/212** |
| 286 | C | 288 | 204/204 | 247/251 | 161/161 | 203/203 | 135/135 | 160/192 | 156/156 | 160/182 | 243/243 | 124/173 | 172/176 | 183/183 | 210/210 | 207/212 | 118/141 | 199/217 | 133/184 | 119/127 | 152/157 | 172/212 |
| **287** | **C & M** | **289** | **204/204** | **247/251** | **161/161** | **203/203** | **135/135** | **160/192** | **154/154** | **160/178** | **243/243** | **124/173** | **172/176** | **183/183** | **210/210** | **207/212** | **118/141** | **199/217** | **133/184** | **119/127** | **152/157** | **172/212** |
| 288 | C | 290 | 204/204 | 247/251 | 161/161 | 203/203 | 135/135 | 160/192 | 154/154 | 164/182 | 243/243 | 124/173 | 172/176 | 183/183 | 210/210 | 207/212 | 118/141 | 199/217 | 133/184 | 119/127 | 152/157 | 172/212 |
| 289 | C | 291 | 204/204 | 249/251 | 161/161 | 203/203 | 135/135 | 160/192 | 154/154 | 160/182 | 243/243 | 124/173 | 172/176 | 183/183 | 210/210 | 207/212 | 118/141 | 199/217 | 133/184 | 119/127 | 152/157 | 172/212 |
| 290 | C | 292 | 204/212 | 243/247 | 130/130 | 203/207 | 129/135 | 182/192 | 154/154 | 160/178 | 243/243 | 122/173 | 174/176 | 183/183 | 210/220 | 207/212 | 118/121 | 197/217 | 147/184 | 114/119 | 152/160 | 172/218 |
| 291 | C | 293 | 204/214 | 243/247 | 130/130 | 203/203 | 135/139 | 182/204 | 194/194 | 140/178 | 254/254 | 122/124 | 168/168 | 183/183 | 210/210 | 207/212 | 118/121 | 191/217 | 184/184 | 127/134 | 154/157 | 212/216 |
| 292 | C | 294 | 204/206 | 243/247 | 152/159 | 197/203 | 129/141 | 186/202 | 152/152 | 140/182 | 243/243 | 122/124 | 166/185 | 183/193 | 206/206 | 212/212 | 124/147 | 191/199 | 141/147 | 116/127 | 152/160 | 169/175 |
| 293 | C | 295 | 204/204 | 243/247 | 129/161 | 203/203 | 137/137 | 192/192 | 218/238 | 140/160 | 243/243 | 122/173 | 166/172 | 183/185 | 210/210 | 212/212 | 124/141 | 205/217 | 133/147 | 125/127 | 157/157 | 204/214 |
| 294 | C | 296 | 204/214 | 243/247 | 130/161 | 203/203 | 139/139 | 192/204 | 194/218 | 140/178 | 243/254 | 122/124 | 166/172 | 183/183 | 210/220 | 212/212 | 121/141 | 191/217 | 133/133 | 127/134 | 154/157 | 210/216 |
| 295 | C | 297 | 204/214 | 243/247 | 130/161 | 203/203 | 135/139 | 192/204 | 154/154 | 174/178 | 243/243 | 124/152 | 168/172 | 183/183 | 210/220 | 212/212 | 118/127 | 191/199 | 176/184 | 119/134 | 152/154 | 206/216 |
| 296 | C | 298 | 204/212 | 243/247 | 130/165 | 203/203 | 135/139 | 192/204 | 154/154 | 140/160 | 254/254 | 152/173 | 168/172 | 183/183 | 210/220 | 207/212 | 121/141 | 191/199 | 188/188 | 119/134 | 152/154 | 208/216 |
| 297 | C | 299 | 204/214 | 243/247 | 130/161 | 203/203 | 139/139 | 192/204 | 154/154 | 160/178 | 243/243 | 152/173 | 168/176 | 183/183 | 210/220 | 212/212 | 118/127 | 191/217 | 133/133 | 119/134 | 152/154 | 172/216 |
| 298 | C | 300 | 204/212 | 237/247 | 130/130 | 203/203 | 137/137 | 192/206 | 154/154 | 140/178 | 243/243 | 152/165 | 168/172 | 183/185 | 210/210 | 207/212 | 121/141 | 197/217 | 133/147 | 116/119 | 152/168 | 172/214 |
| **299** | **C & M** | **301** | **204/212** | **243/247** | **138/161** | **203/203** | **135/137** | **192/206** | **154/154** | **140/160** | **243/254** | **122/173** | **166/176** | **183/183** | **210/220** | **207/212** | **118/127** | **197/199** | **147/184** | **119/134** | **152/154** | **172/212** |
| **300** | **C & M** | **302** | **204/204** | **243/249** | **152/163** | **201/203** | **129/137** | **170/192** | **152/152** | **140/162** | **243/243** | **124/124** | **172/172** | **181/196** | **210/216** | **212/212** | **124/124** | **191/217** | **147/171** | **116/119** | **152/152** | **172/216** |
| 301 | C | 303 | 204/204 | 243/249 | 159/159 | 203/203 | 134/149 | 174/202 |  | 146/146 | 243/243 | 122/122 | 183/185 |  | 212/218 | 212/212 | 121/124 | 191/203 | 133/143 | 127/127 | 152/160 | 170/170 |
| 302 | C | 304 | 204/206 | 243/249 | 152/159 | 201/207 | 129/135 | 196/202 | 216/216 | 146/178 | 243/243 | 122/124 | 172/172 | 181/196 | 210/220 | 212/212 | 124/141 | 189/217 | 141/171 | 114/119 | 152/160 | 172/208 |
| 303 | C | 305 | 212/214 | 243/253 | 130/161 | 203/203 | 129/139 | 160/182 | 188/188 | 140/178 | 254/254 | 122/152 | 168/172 | 185/193 | 206/210 | 207/212 | 118/121 | 191/197 | 147/147 | 125/134 | 154/154 | 172/212 |
| 304 | C | 306 | 204/212 | 243/253 | 130/130 | 203/203 | 139/139 | 160/182 | 154/154 | 160/178 | 243/243 | 152/173 | 168/176 | 183/183 | 210/220 | 207/212 | 118/121 | 191/217 | 133/133 | 116/119 | 152/168 | 162/212 |
| 305 | C | 307 | 204/212 | 243/253 | 130/130 | 203/203 | 139/139 | 160/182 | 154/154 | 160/178 | 263/263 | 152/173 | 168/176 | 183/183 | 210/220 | 207/212 | 118/121 | 191/217 | 133/133 | 116/119 | 152/168 | 172/212 |
| 306 | C | 308 | 204/212 | 243/253 | 130/130 | 203/203 | 139/139 | 160/182 | 154/154 | 160/178 | 263/263 | 152/173 | 168/176 | 183/183 | 210/220 | 207/212 | 118/121 | 191/217 | 133/133 | 116/119 | 152/168 | 162/212 |
| 307 | C | 309 | 204/204 | 229/232 | 152/161 | 203/203 | 123/137 | 170/186 | 180/180 | 142/146 | 243/243 | 177/177 | 166/174 | 183/196 | 216/216 | 207/210 | 121/135 | 193/217 | 147/204 | 119/119 | 152/152 | 175/175 |
| 308 | C | 310 | 204/212 | 237/247 | 130/161 | 203/203 | 137/137 | 160/204 | 154/154 | 160/178 | 263/263 | 152/173 | 166/172 | 183/185 | 210/220 | 212/212 | 118/127 | 197/199 | 147/184 | 127/131 | 157/168 | 172/218 |
| 309 | C | 311 | 204/212 | 237/247 | 130/161 | 203/203 | 137/137 | 160/204 | 154/154 | 160/178 | 243/263 | 152/173 | 166/172 | 183/185 | 210/220 | 212/212 | 118/127 | 197/199 | 147/184 | 127/134 | 157/168 | 172/216 |
| 310 | C | 312 | 212/214 | 243/253 | 138/159 | 195/203 |  | 170/184 |  | 140/178 | 243/243 | 122/148 | 168/172 | 189/193 | 206/210 | 207/212 | 121/141 | 191/197 | 147/165 | 116/127 |  | 175/175 |
| **311** | **C & M** | **313** | **212/214** | **243/253** | **138/161** | **203/203** | **129/137** | **184/204** | **154/154** | **160/178** | **254/254** | **122/154** | **166/176** | **185/193** | **210/220** | **212/212** | **121/121** | **191/197** | **147/147** | **127/134** | **154/157** | **216/216** |
| 312 | C | 314 | 212/214 | 243/253 | 138/161 | 203/203 |  | 184/204 |  | 160/178 | 254/254 | 122/154 | 166/176 | 183/189 | 210/220 | 212/212 | 121/121 | 191/197 | 147/147 | 127/134 |  | 206/216 |
| 313 | C | 315 | 204/212 | 243/255 | 130/130 | 203/203 | 135/137 | 182/192 | 154/154 | 140/182 | 243/243 | 152/175 | 166/176 | 183/183 | 210/220 | 207/212 | 118/121 | 197/199 | 147/176 | 116/119 | 152/168 | 172/210 |
| 314 | C | 316 | 204/214 | 243/251 | 130/130 | 203/203 | 135/139 | 160/182 | 194/218 | 160/178 | 254/254 | 124/152 | 166/176 | 183/183 | 210/210 | 207/212 | 127/141 | 191/199 | 184/184 | 119/134 | 152/154 | 208/212 |
| **315** | **C & M** | **317** | **204/212** | **243/251** | **130/130** | **203/203** | **139/139** | **160/182** | **154/154** | **160/178** | **263/263** | **152/173** | **168/176** | **183/183** | **210/220** | **207/212** | **118/121** | **191/217** | **133/133** | **116/119** | **152/168** | **172/212** |
| **316** | **C & M** | **318** | **204/212** | **243/251** | **130/130** | **203/203** | **139/139** | **160/182** | **154/154** | **160/178** | **243/263** | **152/175** | **168/176** | **183/183** | **210/220** | **207/212** | **118/121** | **191/219** | **133/133** | **116/119** | **152/168** | **172/212** |
| **317** | **C & M** | **319** | **204/214** | **243/251** | **130/161** | **203/203** | **139/139** | **160/184** | **154/218** | **160/178** | **243/263** | **124/152** | **168/172** | **183/183** | **210/220** | **212/212** | **118/127** | **191/217** | **133/133** | **127/134** | **154/157** | **210/216** |
| 318 | C | 320 | 204/204 | 243/251 | 136/136 | 203/203 | 123/135 | 160/192 | 218/218 | 178/178 | 243/243 | 152/177 | 168/176 | 183/183 | 210/220 | 207/225 | 118/124 | 183/217 | 133/157 | 119/131 | 152/152 | 172/172 |
| 319 | C | 321 | 204/212 | 243/251 | 159/161 | 203/205 | 129/135 | 160/192 | 152/152 | 146/182 | 263/263 | 152/179 | 172/174 | 181/183 | 206/210 | 212/212 | 118/124 | 189/217 | 133/174 | 116/119 | 152/168 | 172/216 |
| 320 | C | 322 | 204/212 | 243/251 | 130/130 | 203/203 | 137/137 | 160/204 | 194/218 | 140/160 | 254/254 | 152/173 | 168/176 | 183/185 | 210/210 | 212/212 | 121/141 | 197/217 | 133/147 | 119/134 | 154/157 | 210/214 |
| 321 | C | 323 | 204/212 | 243/251 | 130/130 | 203/203 | 137/137 | 160/200 | 194/218 | 140/160 | 254/254 | 152/173 | 168/176 | 183/185 | 210/210 | 212/212 | 121/141 | 197/217 | 133/147 | 119/134 | 154/157 | 208/208 |
| **322** | **C & M** | **324** | **204/212** | **243/251** | **130/130** | **203/203** | **137/137** | **160/200** | **194/218** | **140/160** | **254/254** | **152/173** | **168/176** | **183/185** | **210/210** | **212/212** | **121/141** | **197/217** | **133/147** | **119/134** | **154/157** | **210/210** |
| **323** | **C & M** | **325** | **204/212** | **243/251** | **130/130** | **203/203** | **137/137** | **160/204** | **194/218** | **140/160** | **254/254** | **152/173** | **168/176** | **183/185** | **210/210** | **212/212** | **121/141** | **197/217** | **133/147** | **119/131** | **154/157** | **210/212** |
| **324** | **C & M** | **326** | **204/212** | **243/251** | **130/130** | **203/203** | **137/137** | **160/204** | **194/218** | **140/160** | **254/254** | **152/173** | **168/176** | **183/185** | **210/210** | **212/212** | **121/141** | **197/217** | **133/147** | **119/134** | **154/157** | **210/212** |
| 325 | C | 327 | 204/212 | 243/251 | 130/130 | 203/203 | 137/137 | 160/200 | 194/218 | 140/160 | 254/254 | 152/173 | 168/176 | 183/185 | 210/210 | 212/212 | 121/141 | 197/217 | 133/147 | 119/134 | 154/157 | 208/210 |
| **326** | **C & M** | **328** | **204/212** | **237/251** | **130/161** | **191/203** | **137/137** | **160/170** | **154/154** | **146/178** | **243/243** | **122/124** | **174/176** | **183/189** | **210/210** | **212/212** | **127/141** | **205/217** | **133/147** | **125/127** | **157/157** | **212/212** |
| 327 | C | 329 | 204/214 | 243/251 | 130/161 | 203/203 | 139/139 | 160/202 | 218/218 | 140/160 | 254/254 | 124/152 | 168/176 | 183/183 | 210/220 | 207/212 | 127/141 | 191/217 | 133/133 | 119/134 | 152/154 | 208/212 |
| 328 | C | 330 | 204/212 | 243/251 | 130/130 | 203/203 | 137/137 | 160/208 | 194/218 | 140/180 | 243/254 | 152/173 | 166/172 | 183/185 | 210/210 | 207/212 | 127/141 | 197/217 | 133/147 | 116/119 | 152/168 | 172/216 |
| **329** | **C & M** | **331** | **204/212** | **243/251** | **130/130** | **203/203** | **137/137** | **160/204** | **194/218** | **140/180** | **243/254** | **152/177** | **166/172** | **183/185** | **210/210** | **207/212** | **127/141** | **197/217** | **133/147** | **116/119** | **152/168** | **172/216** |
| **330** | **C & M** | **332** | **204/212** | **243/251** | **130/130** | **203/203** | **137/137** | **160/204** | **194/218** | **140/180** | **243/254** | **152/173** | **166/172** | **183/185** | **210/210** | **207/212** | **127/141** | **197/217** | **133/147** | **116/119** | **152/168** | **172/216** |
| 331 | C | 333 | 204/212 | 243/251 | 130/130 | 203/203 | 137/137 | 160/204 | 194/218 | 140/180 | 243/254 | 152/173 | 166/172 | 183/185 | 206/210 | 207/212 | 127/141 | 197/217 | 133/147 | 116/119 | 152/168 | 172/218 |
| 332 | C | 335 | 204/212 | 243/251 | 130/130 | 203/203 | 135/139 | 160/204 | 194/218 | 140/160 | 254/254 | 152/173 | 168/172 | 183/183 | 210/220 | 207/212 | 127/141 | 191/199 | 184/184 | 127/134 | 154/157 | 208/218 |
| **333** | **C & M** | **336** | **204/212** | **243/251** | **130/130** | **203/203** | **135/139** | **160/204** | **194/218** | **140/160** | **254/254** | **152/173** | **168/172** | **183/183** | **210/220** | **207/212** | **127/141** | **191/199** | **184/184** | **127/134** | **154/157** | **208/212** |
| 334 | C | 337 | 204/212 | 243/251 | 130/130 | 203/203 | 137/137 | 160/204 | 194/218 | 140/160 | 254/254 | 152/173 | 168/176 | 183/185 | 210/210 | 212/212 | 121/141 | 197/217 | 133/147 | 119/134 | 154/157 | 208/212 |
| 335 | C | 338 |  | 229/229 |  |  |  | 160/192 |  | 146/185 | 243/243 | 122/173 | 172/185 |  | 206/210 |  | 118/121 | 189/199 | 147/184 | 114/127 |  | 170/218 |
| 336 | C | 339 | 204/204 | 241/243 | 163/186 | 191/203 | 129/134 | 192/204 | 238/238 | 140/146 | 243/243 | 122/152 | 166/174 | 181/185 | 206/206 | 212/212 | 121/127 | 191/197 | 147/171 | 114/125 | 160/160 | 175/216 |
| 337 | C | 340 | 204/212 | 237/251 | 130/161 | 203/203 | 137/137 | 160/182 | 154/154 | 140/160 | 243/254 | 124/152 | 168/172 | 181/183 | 210/210 | 212/212 | 118/127 | 191/217 | 133/147 | 116/119 | 152/168 | 172/212 |
| **338** | **C & M** | **341** | **204/212** | **237/251** | **130/161** | **203/203** | **135/139** | **192/204** | **218/218** | **178/178** | **243/243** | **124/152** | **168/172** | **183/185** | **210/220** | **207/212** | **118/121** | **197/199** | **184/184** | **116/119** | **152/168** | **208/216** |
| 339 | C | 342 | 214/223 | 243/251 | 163/163 | 203/203 | 135/159 | 170/170 | 156/156 | 140/178 | 243/243 | 148/173 | 162/172 | 183/183 | 210/220 | 212/212 | 121/141 | 191/199 | 157/184 | 119/125 | 152/152 | 208/214 |
| 340 | C | 343 | 204/212 | 243/251 | 159/159 | 203/203 | 137/137 | 170/192 | 154/154 | 146/160 | 243/243 | 124/177 | 170/172 | 181/183 | 210/210 | 207/212 | 121/141 | 197/205 | 137/171 | 119/127 | 154/168 | 172/214 |
| **341** | **C & M** | **344** | **212/214** | **243/251** | **161/163** | **199/203** | **137/161** | **170/204** | **154/154** | **126/178** | **243/243** | **122/122** | **166/168** | **185/185** | **206/206** | **207/207** | **121/127** | **191/205** | **147/157** | **114/125** | **160/160** | **172/216** |
| 342 | C | 345 | 204/214 | 243/251 | 130/161 | 203/203 | 137/137 | 182/192 | 154/154 | 160/178 | 243/243 | 122/173 | 166/172 | 183/185 | 210/220 | 212/212 | 118/121 | 197/217 | 133/147 | 119/134 | 152/154 | 172/212 |
| 343 | C | 346 | 204/212 | 243/251 | 138/161 | 203/203 | 139/139 | 182/192 |  | 140/160 | 254/254 | 152/177 | 166/172 | 183/183 | 210/220 | 212/212 | 118/127 | 191/217 | 133/133 | 116/119 | 152/168 | 204/208 |
| 344 | C | 347 | 204/212 | 243/251 | 130/161 | 203/203 | 137/137 | 182/192 | 154/154 | 160/178 | 263/263 | 152/173 | 166/176 | 183/185 | 210/220 | 212/212 | 118/121 | 197/217 | 133/147 | 119/134 | 152/154 | 208/216 |
| 345 | C | 348 | 204/212 | 237/251 | 130/130 | 203/203 | 135/137 | 182/192 | 218/218 | 176/178 | 243/243 | 122/124 | 168/172 | 183/183 | 210/210 | 207/212 | 127/141 | 191/199 | 147/184 | 119/131 | 152/154 | 172/212 |
| 346 | C | 349 | 204/214 | 243/251 | 130/130 | 203/203 | 137/137 | 182/192 | 154/154 | 178/178 | 243/254 | 122/175 | 168/172 | 183/185 | 210/220 | 207/212 | 124/141 | 197/217 | 133/147 | 116/119 | 152/168 | 172/210 |
| 347 | C | 350 | 204/214 | 243/251 | 130/157 | 203/203 | 139/139 | 182/192 | 154/154 | 140/160 | 243/254 | 122/124 | 168/172 | 183/183 | 210/220 | 207/212 | 121/141 | 191/217 | 133/133 | 119/134 | 152/154 | 172/212 |
| 348 | C | 351 | 204/214 | 243/251 | 130/161 | 203/203 | 135/139 | 182/192 | 154/154 | 160/178 | 243/263 | 152/173 | 168/172 | 183/185 | 210/210 | 207/212 | 118/121 | 191/199 | 184/184 | 119/134 | 152/154 | 208/208 |
| **349** | **C & M** | **352** | **204/212** | **243/251** | **138/161** | **203/203** | **137/137** | **182/192** | **154/154** | **140/182** | **263/263** | **152/173** | **168/172** | **183/185** | **210/220** | **212/212** | **118/121** | **197/217** | **133/147** | **119/134** | **152/154** | **208/216** |
| 350 | C | 353 | 204/212 | 243/251 | 138/161 | 203/203 | 137/137 | 182/192 | 154/154 | 140/178 | 243/263 | 152/173 | 168/172 | 183/185 | 210/220 | 212/212 | 118/121 | 197/217 | 133/147 | 119/134 | 152/154 | 208/216 |
| **351** | **C & M** | **354** | **204/214** | **243/251** | **130/130** | **203/203** | **139/139** | **182/192** | **218/218** | **178/178** | **243/254** | **152/173** | **168/176** | **183/183** | **210/220** | **212/212** | **121/141** | **191/217** | **133/133** | **119/134** | **152/154** | **208/212** |
| 352 | C | 355 | 204/214 | 243/251 | 138/161 | 203/209 | 135/135 | 192/200 | 154/178 | 140/154 | 254/254 | 144/173 | 176/180 | 183/183 | 210/222 | 207/212 | 124/141 | 183/217 | 133/157 | 114/119 | 152/152 | 166/172 |
| 353 | C | 356 | 204/212 | 243/251 | 130/130 | 203/203 | 135/137 | 192/202 | 154/154 | 160/178 | 243/243 | 122/173 | 168/172 | 183/185 | 210/220 | 207/212 | 127/141 | 197/199 | 147/184 | 119/134 | 152/154 | 172/216 |
| 354 | C | 357 | 204/214 | 243/251 | 130/161 | 203/203 | 135/139 | 192/204 | 154/154 | 160/178 | 243/243 | 124/152 | 166/176 | 183/183 | 210/220 | 207/212 | 118/127 | 191/199 | 184/184 | 127/134 | 154/157 | 172/216 |
| 355 | C | 358 | 204/212 | 243/251 | 130/161 | 203/203 |  | 192/204 |  | 140/178 | 243/243 | 122/124 | 166/176 | 183/185 | 210/210 |  | 118/127 | 197/217 | 133/147 | 119/134 |  | 208/212 |
| **356** | **C & M** | **359** | **204/212** | **243/251** | **130/130** | **203/203** | **135/139** | **192/204** | **154/154** | **156/178** | **243/254** | **122/124** | **168/172** | **183/183** | **210/220** | **212/212** | **118/121** | **191/199** | **184/184** | **119/134** | **152/154** | **172/216** |
| **357** | **C & M** | **360** | **204/212** | **243/251** | **130/161** | **203/203** | **139/139** | **192/204** | **154/154** | **160/178** | **263/263** | **152/173** | **168/172** | **183/183** | **210/210** | **207/212** | **127/141** | **191/217** | **133/133** | **119/134** | **152/154** | **208/212** |
| **358** | **C & M** | **361** | **204/212** | **243/251** | **130/130** | **203/203** | **137/137** | **192/204** | **219/219** | **140/178** | **243/254** | **152/173** | **168/176** | **183/183** | **210/220** | **207/212** | **118/121** | **191/217** | **133/147** | **116/119** | **152/168** | **208/212** |
| 359 | C | 362 | 204/214 | 243/251 | 130/130 | 203/203 | 137/137 | 190/204 | 154/154 | 140/178 | 243/243 | 122/173 | 168/172 | 183/185 | 210/210 | 207/212 | 124/141 | 197/217 | 133/147 | 116/119 | 152/168 | 208/212 |
| 360 | C | 363 | 204/204 | 247/247 | 159/159 | 203/203 | 125/134 | 170/186 | 230/230 | 146/160 | 243/243 | 124/181 | 172/178 | 181/181 | 210/210 | 207/212 | 124/141 | 195/197 | 155/171 | 114/127 |  | 172/214 |
| 361 | C | 364 | 204/212 | 247/247 | 159/159 | 203/203 | 125/129 | 170/192 | 152/154 | 160/160 | 263/263 | 122/177 | 170/172 | 183/198 | 206/210 | 207/212 | 118/121 | 189/195 | 147/153 | 103/119 | 160/168 | 172/210 |
| 362 | C | 365 | 204/204 | 247/247 | 159/159 | 203/203 | 129/137 | 186/192 | 228/228 | 146/160 | 263/263 | 122/177 | 170/172 | 181/181 | 210/210 | 207/212 | 118/124 | 189/205 | 137/147 | 119/127 | 154/168 | 172/216 |
| **363** | **C & M** | **366** | **204/212** | **247/249** | **130/161** | **203/203** | **137/137** | **192/204** | **154/154** | **140/160** | **243/243** | **124/152** | **168/168** | **183/183** | **210/220** | **207/212** | **127/141** | **197/217** | **133/147** | **119/134** | **152/154** | **172/216** |
| 364 | C | 367 | 204/204 | 247/251 | 129/159 | 203/205 | 135/135 | 160/180 | 216/216 | 176/182 | 243/243 | 152/183 | 166/172 | 183/183 | 210/210 | 206/212 | 118/121 | 199/217 | 133/178 | 119/127 | 152/152 | 172/175 |
| 365 | C | 368 | 204/214 | 237/251 | 138/161 | 203/203 | 137/137 | 182/192 | 154/154 | 160/178 | 243/243 | 122/177 | 166/172 | 183/185 | 210/210 | 212/212 | 118/127 | 197/217 | 133/147 | 127/134 | 154/157 | 208/216 |
| 366 | C | 369 | 212/214 | 247/251 |  | 203/207 | 137/139 | 182/192 | 154/154 | 140/166 | 254/254 | 124/124 | 166/176 | 183/185 | 206/220 | 212/212 | 127/141 | 191/197 | 147/147 | 114/116 | 160/168 | 202/216 |
| **367** | **C & M** | **370** | **204/204** | **247/251** | **159/159** | **203/203** | **125/137** | **186/192** | **154/154** | **146/160** | **243/243** | **124/177** | **170/178** | **181/198** | **210/210** | **212/212** | **118/141** | **195/205** | **137/153** | **127/127** | **154/160** | **172/175** |
| 368 | C | 371 | 204/204 | 249/249 | 129/159 | 201/207 | 134/137 | 170/170 | 154/154 | 150/162 | 263/263 | 124/124 | 172/178 | 183/196 | 206/210 | 212/212 | 124/124 | 197/199 | 147/171 | 119/119 | 160/160 | 170/170 |
| 369 | C | 372 | 204/204 | 251/251 | 116/132 | 201/203 | 129/135 | 160/202 | 148/148 | 146/164 | 243/243 | 152/173 | 168/172 | 181/183 | 210/216 | 207/212 | 118/141 | 189/199 | 171/184 | 114/127 | 157/160 | 208/214 |
| **370** | **C & M** | **373** | **204/212** | **251/253** | **129/161** | **203/203** | **129/145** | **160/180** | **218/218** | **140/178** | **243/243** | **144/173** | **172/176** | **183/193** | **206/210** | **212/212** | **118/121** | **189/195** | **147/157** | **119/125** | **152/152** | **172/216** |
| 371 | C | 374 | 204/214 | 251/255 | 138/161 | 207/211 | 129/151 | 164/182 | 152/154 | 130/182 | 254/254 | 122/152 | 166/166 | 181/183 | 206/216 | 207/207 | 121/124 | 189/199 | 157/186 | 127/131 | 160/160 | 175/181 |
| 372 | C | 375 | 204/204 | 241/243 | 159/186 | 203/203 | 129/137 | 170/184 | 194/194 | 146/172 | 243/243 | 148/152 | 166/174 | 181/185 | 206/210 | 212/212 | 124/141 | 189/197 | 147/171 | 114/125 | 160/160 | 214/214 |
| **373** | **C & M** | **376** | **204/212** | **229/247** | **130/161** | **191/203** | **135/135** | **160/170** | **152/154** | **148/160** | **243/243** | **124/152** | **166/176** | **183/189** | **210/216** | **207/212** | **141/141** | **197/199** | **171/184** | **116/119** | **152/168** | **208/208** |
| 374 | C | 377 | 204/214 | 237/251 | 130/161 | 203/203 | 135/137 | 160/204 | 154/154 | 140/178 | 243/254 | 122/173 | 168/172 | 183/185 | 210/210 | 207/212 | 118/127 | 197/199 | 147/184 | 127/134 | 154/157 | 172/216 |
| 375 | C | 378 | 204/204 | 239/241 | 130/161 | 199/203 | 135/137 | 160/184 | 152/156 | 126/140 | 243/243 | 148/163 | 166/172 | 189/193 | 206/206 | 212/212 | 121/127 | 191/205 | 147/157 | 127/138 | 152/157 | 208/212 |
| **376** | **C & M** | **379** | **204/214** | **229/247** | **161/186** | **203/203** | **137/137** | **184/192** | **194/218** | **130/178** | **243/243** | **144/173** | **172/174** | **183/189** | **210/210** | **207/212** | **118/121** | **205/217** | **133/133** | **119/131** | **152/154** | **208/208** |
| 377 | C | 380 | 204/212 | 243/247 | 159/159 | 203/203 | 129/129 | 170/202 | 218/228 | 156/160 | 243/243 | 124/228 | 172/172 | 181/183 | 210/210 | 207/212 | 121/141 | 189/189 | 147/171 | 119/119 | 152/168 | 216/216 |
| **378** | **C & M** | **381** | **204/214** | **237/251** | **138/161** | **203/203** | **135/137** | **160/182** | **154/154** | **140/160** | **243/254** | **122/173** | **166/176** | **183/185** | **210/220** | **207/212** | **118/121** | **197/199** | **147/184** | **119/134** | **152/154** | **172/212** |
| **379** | **C & M** | **382** | **204/214** | **237/247** | **130/161** | **203/203** | **135/139** | **182/192** | **154/154** | **160/178** | **243/263** | **122/124** | **168/176** | **183/183** | **210/210** | **212/212** | **121/141** | **191/199** | **184/184** | **127/134** | **154/157** | **172/212** |
| **380** | **C & M** | **383** | **204/212** | **229/251** | **129/129** | **203/205** | **129/134** | **160/202** | **152/152** | **140/178** | **243/243** | **122/173** | **172/176** | **181/183** | **206/210** | **212/212** | **124/141** | **189/199** | **147/171** | **114/127** | **157/160** | **208/208** |
| 381 | C | 384 | 204/212 | 237/247 | 138/161 | 203/203 | 135/139 | 182/192 | 194/218 | 140/178 | 243/254 | 122/124 | 166/176 | 183/183 | 210/210 | 212/212 | 121/141 | 191/199 | 133/133 | 116/119 | 152/168 | 172/216 |
| **382** | **C & M** | **385** | **204/212** | **237/251** | **130/161** | **203/203** | **135/137** | **192/204** | **194/218** | **160/180** | **243/243** | **152/173** | **168/176** | **183/185** | **210/210** | **212/212** | **118/121** | **197/199** | **147/184** | **127/134** | **154/157** | **208/216** |
| 383 | C | 386 | 204/212 | 237/251 | 130/161 | 203/203 | 135/137 | 192/204 | 194/218 | 160/178 | 243/243 | 152/173 | 168/176 | 183/185 | 210/210 | 212/212 | 118/121 | 197/199 | 147/184 | 127/134 | 154/157 | 208/216 |
| 384 | C | 387 | 204/212 | 237/251 | 130/161 | 203/203 | 135/137 | 192/204 | 194/218 | 158/178 | 243/243 | 152/173 | 168/176 | 183/185 | 210/210 | 212/212 | 118/121 | 197/199 | 147/184 | 127/134 | 154/157 | 208/216 |
| 385 | C | 388 | 204/212 | 243/247 | 130/165 | 203/203 | 135/139 | 192/204 | 218/218 | 140/160 | 254/254 | 152/173 | 168/172 | 183/183 | 210/220 | 207/212 | 121/141 | 191/199 | 133/184 | 119/134 | 152/154 | 208/216 |
| 386 | C | 389 | 204/214 | 237/247 | 138/161 | 203/203 | 135/137 | 160/182 | 218/218 | 140/178 | 243/254 | 152/173 | 168/176 | 183/185 | 210/220 | 207/212 | 118/127 | 197/217 | 147/184 | 127/134 | 154/157 | 172/212 |
| 387 | C | 390 | 204/214 | 237/247 | 130/145 |  |  | 192/194 |  | 140/180 | 243/254 | 122/124 | 168/172 |  | 210/210 | 207/212 | 121/141 |  | 133/133 | 116/119 |  | 172/212 |
| 388 | C | 391 | 204/214 | 243/247 | 138/138 | 203/203 | 123/135 | 160/174 | 166/166 | 178/178 | 243/243 | 152/173 | 166/172 | 183/183 | 210/220 | 207/242 | 118/124 | 183/199 | 157/184 | 119/134 | 152/154 | 172/216 |
| 389 | C | 392 | 204/212 | 229/241 | 130/163 | 203/203 | 129/134 | 202/204 | 152/152 | 146/174 | 243/243 | 148/152 | 166/174 | 181/185 | 206/206 | 212/212 | 124/127 | 189/197 | 171/171 | 125/127 | 160/160 | 175/175 |
| **390** | **C & M** | **393** | **204/204** | **229/229** | **129/161** | **203/203** | **129/135** | **160/192** | **218/218** | **146/182** | **243/243** | **122/173** | **172/180** | **181/183** | **206/210** | **207/212** | **118/121** | **189/199** | **147/184** | **114/127** | **157/160** | **170/216** |
| 391 | C | 394 | 204/214 | 237/247 | 129/161 | 195/203 | 134/139 | 178/192 | 154/172 | 140/160 | 243/243 | 124/159 | 166/176 | 183/183 | 206/210 | 207/212 | 118/124 | 197/199 | 159/186 | 127/131 | 157/157 | 208/214 |
| 392 | C | 395 | 204/212 | 229/241 | 138/186 | 203/211 | 137/137 | 164/170 | 156/156 | 130/178 | 243/243 | 148/209 | 172/174 | 183/189 | 206/218 | 207/212 | 124/141 | 183/205 | 147/157 | 116/119 | 168/168 | 175/202 |
| 393 | C | 396 | 204/206 | 229/234 | 159/159 | 197/201 | 129/143 | 174/202 | 152/152 | 140/180 | 243/243 | 122/173 | 162/174 | 181/183 | 206/206 | 210/212 | 124/147 | 189/199 | 137/171 | 114/116 | 152/160 | 169/216 |
| 394 | C | 397 | 204/204 | 229/237 | 129/186 | 191/203 | 129/134 | 184/204 | 152/152 | 140/146 | 243/243 | 122/148 | 168/172 | 185/193 | 216/216 | 212/212 | 121/127 | 191/197 | 147/171 | 116/127 | 160/168 | 175/175 |
| 395 | C | 399 | 204/204 | 229/243 | 145/159 | 203/203 | 134/151 | 180/202 | 182/182 | 146/152 | 243/245 | 122/226 | 166/174 | 181/183 | 206/210 | 212/212 | 124/141 | 189/197 | 137/141 | 119/127 | 152/152 | 218/218 |
| 396 | C | 400 | 204/272 | 234/241 | 129/130 | 195/203 | 139/139 | 180/204 | 176/176 | 130/178 | 243/243 | 148/154 | 172/174 | 183/189 | 206/210 | 212/221 | 121/141 | 183/197 | 159/171 | 114/125 | 160/160 | 175/214 |
| 397 | C | 404 | 204/214 | 237/247 | 130/130 | 203/203 | 135/141 | 182/192 | 154/154 | 140/160 | 263/263 | 152/175 | 168/176 | 183/183 | 210/220 | 207/212 | 118/121 | 191/199 | 184/184 | 116/119 | 152/168 | 172/212 |
| **398** | **C & M** | **405** | **204/212** | **237/247** | **130/186** | **191/203** | **129/135** | **192/192** | **194/218** | **140/160** | **254/254** | **124/169** | **168/172** | **181/183** | **210/210** | **207/212** | **127/127** | **191/217** | **133/147** | **114/119** | **152/160** | **175/175** |
| **399** | **C & M** | **406** | **204/214** | **237/251** | **130/161** | **203/203** | **137/137** | **160/182** | **154/154** | **140/160** | **254/254** | **122/124** | **168/172** | **183/185** | **210/210** | **207/212** | **118/127** | **197/217** | **133/133** | **116/119** | **152/168** | **172/212** |
| **400** | **C & M** | **407** | **204/212** | **237/251** | **130/130** | **203/203** | **137/137** | **192/204** | **154/154** | **140/160** | **243/254** | **152/173** | **168/176** | **183/185** | **210/220** | **212/212** | **118/127** | **197/217** | **133/147** | **127/134** | **152/154** | **172/212** |
| **401** | **C & M** | **409** | **212/212** | **241/243** | **184/194** | **195/203** | **137/149** | **184/206** | **146/146** | **146/146** | **243/243** | **148/175** | **168/180** | **185/185** | **210/220** | **212/212** | **124/127** | **183/205** | **147/159** | **103/119** | **152/154** | **172/175** |
| **402** | **C & M** | **410** | **204/204** | **241/247** | **159/163** | **203/205** | **129/135** | **170/192** | **154/154** | **146/160** | **243/243** | **124/148** | **172/172** | **181/183** | **206/210** | **212/212** | **124/141** | **189/217** | **133/171** | **119/127** | **152/160** | **172/175** |
| **403** | **C & M** | **413** | **204/212** | **243/247** | **130/165** | **203/203** | **135/139** | **192/204** | **218/218** | **140/160** | **254/254** | **152/173** | **168/172** | **183/183** | **210/220** | **207/212** | **121/141** | **191/199** | **188/188** | **119/134** | **152/154** | **208/216** |
| 404 | M | 415 | 212/214 | 241/253 | 132/132 | 195/203 | 129/145 | 170/182 | 176/240 | 140/140 | 243/263 | 122/144 | 158/174 | 183/193 | 216/220 | 207/212 | 121/127 | 183/191 | 147/157 | 112/114 | 160/164 | 175/187 |
| 405 | M | 416 | 204/212 | 237/251 | 130/130 | 203/203 | 137/137 | 192/204 | 154/154 | 140/160 | 243/254 | 152/169 | 168/176 | 183/185 | 210/220 | 207/212 | 118/127 | 197/217 | 133/147 | 127/134 | 152/154 |  |
| 406 | M | 417 | 204/204 | 247/251 | 165/165 | 203/203 | 135/135 | 160/192 | 154/154 | 160/180 | 243/243 | 124/173 | 172/180 | 183/183 | 210/210 | 207/212 | 118/141 | 199/217 | 133/184 | 119/127 | 152/157 | 172/210 |
| 407 | M | 418 | 204/212 | 243/251 | 130/130 | 203/203 | 135/137 | 160/204 | 194/221 | 140/180 | 243/254 | 152/173 | 166/172 | 183/185 | 210/210 | 207/212 | 127/141 | 197/217 | 133/147 | 116/119 | 152/168 | 172/216 |
| 408 | M | 419 | 206/212 | 234/243 | 152/163 | 203/205 | 129/137 | 170/192 | 152/152 | 178/182 | 243/243 | 124/173 | 172/172 | 181/187 | 210/216 | 212/212 | 121/124 | 191/217 | 147/171 | 116/127 | 152/160 | 170/216 |
| 409 | M | 420 | 204/204 | 247/251 | 161/161 | 203/203 | 135/135 | 160/192 | 154/154 | 162/180 | 243/243 | 124/173 | 172/180 | 183/183 | 210/210 | 207/212 | 118/141 | 199/217 | 133/188 | 119/127 | 152/157 | 172/210 |
| 410 | M | 421 | 204/212 | 243/247 | 129/161 | 203/205 | 129/134 | 174/184 | 152/152 | 148/178 | 243/243 | 124/152 | 172/172 | 183/193 | 210/216 | 212/212 | 124/124 | 191/197 | 147/171 | 114/119 | 152/160 | 175/216 |
| 411 | M | 422 | 204/212 | 243/247 | 129/165 | 203/205 | 129/134 | 174/184 | 152/152 | 148/178 | 243/243 | 124/152 | 172/172 | 183/193 | 210/216 | 212/212 | 124/124 | 191/197 | 147/171 | 114/119 | 152/160 | 175/216 |
| 412 | M | 423 | 204/204 | 247/251 | 161/161 | 203/203 | 135/135 | 160/192 | 154/154 | 158/180 | 243/243 | 124/173 | 172/180 | 183/183 | 210/210 | 207/212 | 118/141 | 199/217 | 133/184 | 119/127 | 152/157 | 172/210 |
| 413 | M | 424 | 212/268 | 234/251 | 129/130 | 203/203 | 139/145 | 204/204 | 154/176 | 130/140 | 254/254 | 148/171 | 172/176 | 183/183 | 206/210 | 212/221 | 141/141 | 183/195 | 159/184 | 125/134 | 152/154 | 175/210 |
| 414 | M | 425 | 204/212 | 237/247 | 130/161 | 203/203 | 135/137 | 192/204 | 154/154 | 140/160 | 254/263 | 122/173 | 168/176 | 183/185 | 210/220 | 207/212 | 127/141 | 197/199 | 147/171 | 116/119 | 152/168 | 172/216 |
| 415 | M | 426 | 204/212 | 229/243 | 129/159 | 203/205 | 129/129 | 192/202 | 152/152 | 140/146 | 243/243 | 122/152 | 166/172 | 181/193 | 206/216 | 212/212 | 121/124 | 189/191 | 147/171 | 114/127 | 160/160 | 175/216 |
| 416 | M | 427 | 204/212 | 229/243 | 129/159 | 203/205 | 129/129 | 192/202 | 152/152 | 140/146 | 243/243 | 122/152 | 166/172 | 181/193 | 206/216 | 212/212 | 121/124 | 189/191 | 147/186 | 114/127 | 160/160 | 175/216 |
| 417 | M | 428 | 204/212 | 229/243 | 129/159 | 203/205 | 129/129 | 192/202 | 152/152 | 140/146 | 243/243 | 122/154 | 166/172 | 181/193 | 206/216 | 212/212 | 121/124 | 189/191 | 147/171 | 114/127 | 160/160 | 175/216 |
| 418 | M | 429 | 204/212 | 229/243 | 129/159 | 203/205 | 129/129 | 196/202 | 152/152 | 140/146 | 243/243 | 122/152 | 166/172 | 181/193 | 206/216 | 212/212 | 121/124 | 189/191 | 147/171 | 114/127 | 160/160 | 175/216 |
| 419 | M | 430 | 204/212 | 229/243 | 129/159 | 203/205 | 129/132 | 192/202 | 152/152 | 140/146 | 243/243 | 122/152 | 166/172 | 181/193 | 206/216 | 212/212 | 121/124 | 189/191 | 147/176 | 114/127 | 160/160 | 175/214 |
| 420 | M | 431 | 204/214 | 229/247 | 157/182 | 203/203 | 137/137 | 184/192 | 194/218 | 130/178 | 243/243 | 144/173 | 172/174 | 183/189 | 210/210 | 207/212 | 118/121 | 205/217 | 133/133 | 119/131 | 152/154 | 208/208 |
| 421 | M | 432 | 204/214 | 237/247 | 129/161 | 195/203 | 135/141 | 180/192 | 154/172 | 146/178 | 243/243 | 122/124 | 168/176 | 183/183 | 210/220 | 207/212 | 124/141 | 183/199 | 159/186 | 125/127 | 157/157 | 172/175 |
| 422 | M | 433 | 204/206 | 234/247 | 152/163 | 197/207 | 135/141 | 174/186 | 216/226 | 178/182 | 243/243 | 124/154 | 168/185 | 183/183 | 210/239 | 212/212 | 124/147 | 199/199 | 141/186 | 116/119 | 152/152 | 169/210 |
| 423 | M | 434 | 212/212 | 237/237 | 148/190 | 191/205 | 129/134 | 170/192 | 166/166 | 140/178 | 254/263 | 122/169 | 168/172 | 181/185 | 206/210 | 212/212 | 121/127 | 191/197 | 147/171 | 114/116 | 160/168 | 170/175 |
| 424 | M | 435 | 204/204 | 229/251 | 159/161 | 203/203 | 134/135 | 170/192 | 154/154 | 140/160 | 243/243 | 122/173 | 166/180 | 183/185 | 210/210 | 207/212 | 118/124 | 197/199 | 171/184 | 119/125 | 152/152 | 172/175 |
| 425 | M | 436 | 204/212 | 243/247 | 159/159 | 203/203 | 129/134 | 170/192 | 152/152 | 148/160 | 243/243 | 122/124 | 172/172 | 181/183 | 206/210 | 207/212 | 121/124 | 189/197 | 147/171 | 114/119 | 160/168 | 210/214 |
| 426 | M | 437 | 204/212 | 243/247 | 159/159 | 203/203 | 129/134 | 170/192 | 152/152 | 146/160 | 243/243 | 122/124 | 172/172 | 181/183 | 206/210 | 207/212 | 121/124 | 189/197 | 147/171 | 114/119 | 160/168 | 202/210 |
| 427 | M | 438 | 204/212 | 243/247 | 159/159 | 203/203 | 129/134 | 170/192 | 152/152 | 146/160 | 243/243 | 122/124 | 172/172 | 181/183 | 206/210 | 207/212 | 121/124 | 189/197 | 147/171 | 114/119 | 160/168 | 210/221 |
| 428 | M | 439 | 204/212 | 243/249 | 159/159 | 203/203 | 129/134 | 170/192 | 152/152 | 146/160 | 243/243 | 122/124 | 172/172 | 181/183 | 206/210 | 207/212 | 121/124 | 189/197 | 147/171 | 114/119 | 160/168 | 210/216 |
| 429 | M | 440 | 204/212 | 243/247 | 159/159 | 203/203 | 129/134 | 170/192 | 152/154 | 146/160 | 243/243 | 122/124 | 172/172 | 181/183 | 206/210 | 207/212 | 121/124 | 189/197 | 147/171 | 114/119 | 160/168 | 210/216 |
| 430 | M | 441 | 204/212 | 243/247 | 159/159 | 203/203 | 129/134 | 170/192 | 152/152 | 146/160 | 243/243 | 122/124 | 172/174 | 181/183 | 206/210 | 207/212 | 121/124 | 189/197 | 147/171 | 114/119 | 160/168 |  |
| 431 | M | 442 | 204/212 | 243/247 | 159/161 | 203/203 | 129/134 | 170/192 | 152/152 | 146/160 | 243/243 | 122/124 | 172/172 | 181/183 | 206/210 | 207/212 | 121/124 | 189/197 | 147/171 | 114/119 | 160/168 | 210/214 |
| 432 | M | 443 | 204/206 | 234/247 | 152/163 | 197/207 | 135/141 | 174/186 | 216/226 | 178/182 | 243/243 | 124/154 | 168/185 | 183/183 | 210/239 | 212/212 | 124/147 | 199/199 | 141/184 | 116/119 | 152/152 | 169/210 |
| 433 | M | 445 | 204/206 | 234/251 | 152/152 | 203/207 | 137/141 | 186/186 | 158/226 | 160/178 | 243/263 | 124/179 | 168/178 | 181/183 | 210/239 | 212/212 | 118/124 | 199/205 | 137/141 | 116/127 | 152/152 | 172/172 |
| 434 | M | 446 | 204/204 | 229/241 | 190/192 | 191/203 | 137/145 | 180/204 | 156/156 | 140/146 | 263/263 | 122/144 | 158/166 | 183/185 | 206/206 | 212/212 | 127/141 | 191/205 | 147/157 | 114/125 | 160/160 | 185/185 |
| 435 | M | 447 | 204/212 | 243/247 | 138/161 | 203/203 | 137/137 | 160/204 | 154/154 | 140/178 | 243/254 | 152/173 | 166/172 | 183/185 | 210/222 | 207/212 | 121/141 | 197/217 | 133/147 | 119/134 | 152/154 | 172/216 |
| 436 | M | 448 | 204/206 | 234/251 | 152/152 | 203/207 | 137/141 | 186/186 | 158/226 | 160/178 | 243/263 | 124/177 | 168/178 | 181/183 | 210/239 | 212/212 | 118/124 | 199/205 | 137/141 | 116/127 | 152/152 | 172/172 |
| 437 | M | 449 | 204/204 | 229/251 | 129/161 | 191/203 | 134/135 | 160/196 | 152/152 | 146/178 | 243/243 | 124/152 | 174/176 | 183/193 | 210/216 | 212/212 | 118/127 | 191/217 | 133/171 | 114/119 | 152/160 | 170/175 |
| 438 | M | 450 | 212/212 | 237/237 | 148/186 | 191/205 | 129/134 | 170/192 | 166/166 | 140/178 | 254/263 | 122/169 | 168/172 | 181/185 | 206/210 | 212/212 | 121/127 | 191/197 | 147/171 | 114/116 | 160/168 | 170/175 |
| 439 | M | 451 | 204/204 | 229/237 | 130/159 | 191/203 | 135/135 | 192/192 | 154/154 | 140/146 | 243/243 | 122/179 | 170/174 | 189/189 | 210/212 | 207/225 | 118/124 | 191/205 | 133/155 | 116/131 | 168/168 | 175/214 |
| 440 | M | 453 | 204/204 | 229/237 | 130/159 | 191/203 | 135/135 | 188/192 | 154/154 | 140/146 | 243/243 | 122/179 | 170/174 | 189/189 | 210/212 | 207/225 | 118/124 | 191/205 | 133/155 | 116/131 | 168/168 | 175/214 |
| 441 | M | 454 | 204/212 | 243/247 | 130/161 | 203/203 | 135/137 | 182/192 | 154/154 | 140/160 | 254/254 | 152/173 | 166/172 | 183/183 | 210/220 | 212/212 | 118/127 | 191/199 | 147/184 | 127/134 | 154/157 | 202/206 |
| 442 | M | 455 | 204/212 | 243/247 | 130/161 | 203/203 | 135/137 | 182/192 | 154/154 | 140/160 | 254/254 | 152/175 | 166/172 | 183/183 | 210/220 | 212/212 | 118/127 | 191/199 | 147/184 | 127/134 | 154/157 | 202/206 |
| 443 | M | 456 | 204/212 | 243/251 | 130/130 | 203/203 | 137/137 | 160/204 | 194/218 | 140/160 | 254/254 | 152/173 | 168/176 | 183/185 | 210/210 | 212/212 | 121/141 | 197/217 | 133/147 | 119/134 | 154/157 | 210/210 |
| 444 | M | 457 | 204/212 | 243/251 | 129/161 | 203/203 | 129/135 | 192/196 | 154/154 | 160/178 | 243/263 | 148/173 | 168/172 | 181/183 | 206/210 | 207/212 | 118/127 | 189/199 | 133/171 | 114/119 | 152/160 | 172/214 |
| 445 | M | 458 | 212/214 | 229/241 | 129/186 | 203/203 | 137/139 | 182/210 | 162/162 | 140/178 | 243/254 | 144/148 | 174/180 | 183/189 | 210/220 | 212/212 | 121/127 | 191/205 | 147/147 | 116/134 | 154/168 | 175/216 |
| 446 | M | 459 | 212/214 | 229/241 | 129/186 | 203/203 | 137/139 | 182/202 | 162/162 | 140/178 | 243/254 | 144/148 | 174/180 | 183/189 | 210/220 | 212/212 | 121/127 | 191/205 | 147/147 | 116/134 | 154/168 | 175/216 |
| 447 | M | 460 | 212/214 | 229/241 | 129/184 | 203/203 | 137/139 | 182/204 | 162/162 | 140/178 | 243/254 | 144/148 | 174/180 | 183/189 | 210/220 | 212/212 | 121/127 | 191/205 | 147/147 | 116/134 | 154/168 | 175/216 |
| 448 | M | 461 | 212/214 | 229/241 | 129/186 | 203/203 | 137/139 | 182/204 | 162/162 | 140/174 | 243/254 | 144/148 | 174/180 | 183/189 | 210/220 | 212/212 | 121/127 | 191/205 | 147/147 | 116/134 | 154/168 | 175/216 |
| 449 | M | 462 | 204/212 | 241/243 | 130/138 | 199/203 | 129/134 | 170/170 | 162/162 | 140/178 | 254/254 | 122/152 | 174/180 | 183/189 | 210/220 | 210/212 | 124/127 | 191/205 | 171/171 | 116/134 | 154/168 | 175/187 |
| 450 | M | 463 | 204/212 | 243/251 | 129/161 | 203/203 | 129/135 | 192/196 | 154/154 | 160/174 | 243/263 | 148/173 | 168/172 | 181/183 | 206/210 | 207/212 | 118/127 | 189/199 | 133/171 | 114/119 | 152/160 | 172/216 |
| 451 | M | 464 | 204/212 | 241/243 | 130/138 | 199/203 | 129/134 | 170/184 | 162/162 | 140/178 | 254/254 | 122/152 | 174/180 | 183/189 | 210/220 | 210/212 | 124/127 | 191/205 | 171/171 | 116/134 | 154/168 | 175/185 |
| 452 | M | 465 | 204/204 | 237/247 | 161/186 | 203/203 | 135/137 | 170/192 | 194/218 | 146/160 | 243/263 | 148/173 | 174/180 | 183/185 | 210/210 | 207/212 | 118/144 | 197/217 | 133/147 | 116/119 | 152/168 | 172/214 |
| 453 | M | 466 | 204/212 | 237/247 | 130/161 | 203/203 | 137/137 | 192/204 | 154/154 | 140/176 | 243/243 | 148/173 | 168/172 | 183/185 | 210/210 | 212/212 | 121/141 | 189/217 | 133/157 | 103/119 | 152/154 | 172/175 |
| 454 | M | 467 | 204/212 | 237/247 | 130/161 | 203/203 | 137/137 | 192/198 | 154/154 | 140/176 | 243/243 | 148/173 | 168/172 | 183/185 | 210/210 | 212/212 | 121/141 | 189/217 | 133/157 | 103/119 | 152/154 | 172/175 |
| 455 | M | 468 | 204/261 | 241/251 | 129/130 | 195/203 | 135/135 | 160/204 | 154/176 | 130/178 | 254/254 | 148/173 | 172/172 | 183/189 | 206/210 | 212/221 | 121/141 | 197/199 | 171/184 | 114/134 | 154/160 | 210/214 |
| 456 | M | 469 | 204/212 | 243/247 | 188/188 | 203/203 | 129/135 | 184/192 | 154/154 | 178/178 | 243/263 | 148/173 | 168/176 | 181/183 | 206/210 | 212/212 | 118/141 | 189/199 | 171/184 | 114/119 | 152/160 | 210/214 |
| 457 | M | 470 | 204/212 | 243/247 | 180/188 | 203/203 | 129/135 | 184/192 | 154/154 | 140/178 | 243/263 | 148/173 | 168/176 | 181/183 | 206/210 | 212/212 | 118/141 | 189/199 | 171/184 | 114/119 | 152/160 | 210/214 |
| 458 | M | 471 | 204/204 | 247/251 | 159/159 | 203/203 | 125/137 | 186/192 | 154/154 | 146/160 | 243/243 | 124/179 | 170/178 | 181/198 | 210/210 | 212/212 | 118/141 | 195/205 | 137/153 | 127/127 | 154/160 | 172/175 |
| 459 | M | 472 | 204/204 | 247/247 | 159/159 | 203/203 | 129/137 | 186/192 | 228/228 | 146/160 | 263/263 | 122/177 | 170/172 | 181/181 | 210/210 | 207/212 | 118/124 | 189/205 | 137/147 | 119/127 | 152/168 | 172/214 |
| 460 | M | 473 | 204/204 | 247/247 | 159/159 | 203/203 | 129/137 | 186/192 | 228/228 | 146/164 | 263/263 | 122/177 | 170/172 | 181/181 | 210/210 | 207/212 | 118/124 | 189/205 | 137/147 | 119/127 | 152/168 | 172/214 |
| 461 | M | 474 | 204/204 | 229/243 | 145/159 | 203/203 | 134/151 | 180/202 | 182/182 | 146/152 | 243/245 | 122/216 | 166/174 | 181/183 | 206/210 | 212/212 | 124/141 | 189/197 | 137/141 | 119/127 | 152/152 | 216/218 |
| 462 | M | 475 | 212/212 | 229/237 | 130/176 | 191/197 | 125/134 | 170/170 | 170/170 | 140/178 | 263/263 | 122/144 | 170/174 | 183/185 | 210/210 | 210/212 | 121/141 | 191/197 | 157/174 | 125/131 | 152/157 | 166/212 |
| 463 | M | 476 | 204/212 | 243/247 | 159/159 | 203/203 | 129/129 | 170/202 | 218/228 | 156/160 | 243/243 | 124/220 | 172/174 | 181/183 | 210/210 | 207/212 | 121/141 | 189/189 | 147/171 | 119/119 | 152/168 | 216/216 |
| 464 | M | 477 | 204/212 | 243/247 | 159/159 | 203/203 | 129/129 | 170/202 | 218/228 | 156/160 | 243/243 | 124/226 | 172/1747 | 181/183 | 210/210 | 207/212 | 121/141 | 189/189 | 147/171 | 119/119 | 152/168 | 216/216 |
| 465 | M | 478 | 204/212 | 243/247 | 159/159 | 203/203 | 129/129 | 170/202 | 218/228 | 156/160 | 243/243 | 124/216 | 172/174 | 181/183 | 210/210 | 207/212 | 121/141 | 189/189 | 147/171 | 119/119 | 152/168 | 216/216 |
| 466 | M | 479 | 204/212 | 243/247 | 159/159 | 203/203 | 129/129 | 170/202 | 218/228 | 156/160 | 243/243 | 124/222 | 172/174 | 181/183 | 210/210 | 207/212 | 121/141 | 189/189 | 147/171 | 119/119 | 152/168 | 216/216 |
| 467 | M | 480 | 204/212 | 243/247 | 180/180 | 203/203 | 129/135 | 184/192 | 154/154 | 178/178 | 243/263 | 148/173 | 168/176 | 181/183 | 206/210 | 212/212 | 118/141 | 189/199 | 171/184 | 114/119 | 152/160 | 210/214 |
| 468 | M | 481 | 204/204 | 247/251 | 161/161 | 203/203 | 135/135 | 160/192 | 154/154 | 160/178 | 243/243 | 124/173 | 172/187 | 183/183 | 210/210 | 207/212 | 118/141 | 199/217 | 133/184 | 119/127 | 152/157 | 172/212 |
| 469 | M | 482 | 204/204 | 247/251 | 161/161 | 203/203 | 135/135 | 160/192 | 154/154 | 160/178 | 243/243 | 124/173 | 172/180 | 183/183 | 210/210 | 207/212 | 118/141 | 199/217 | 133/184 | 119/127 | 152/157 | 172/212 |
| 470 | M | 483 | 204/204 | 247/251 | 161/161 | 203/203 | 135/135 | 160/192 | 154/154 | 160/178 | 243/243 | 124/177 | 172/183 | 183/183 | 210/210 | 207/212 | 118/141 | 199/217 | 133/184 | 119/127 | 152/157 | 172/208 |
| 471 | M | 484 | 204/212 | 241/247 | 186/186 | 203/203 | 135/137 | 160/170 | 194/219 | 146/178 | 243/243 | 148/173 | 168/172 | 183/189 | 210/210 | 212/212 | 121/141 | 199/205 | 147/184 | 116/127 | 152/157 | 172/210 |
| 472 | M | 485 | 204/212 | 241/247 | 186/186 | 203/203 | 135/137 | 160/170 | 194/219 | 146/178 | 243/243 | 148/173 | 168/172 | 183/189 | 210/210 | 212/212 | 141/141 | 199/205 | 147/188 | 116/127 | 152/157 | 172/210 |
| 473 | M | 486 | 204/212 | 241/251 | 161/192 | 203/203 | 129/135 | 184/192 | 154/154 | 160/178 | 263/263 | 148/173 | 166/172 | 181/183 | 206/210 | 212/212 | 118/124 | 189/199 | 171/184 | 119/125 | 152/152 | 172/214 |
| 474 | M | 487 | 204/212 | 241/247 | 182/182 | 203/203 | 135/137 | 160/170 | 194/219 | 146/178 | 243/243 | 148/173 | 168/172 | 183/189 | 210/210 | 212/212 | 141/141 | 199/205 | 147/184 | 116/127 | 152/157 | 172/210 |
| 475 | M | 488 | 204/212 | 241/251 | 161/188 | 203/203 | 129/135 | 184/192 | 154/154 | 160/178 | 263/263 | 148/173 | 166/172 | 181/183 | 206/210 | 212/212 | 118/124 | 189/199 | 171/184 | 119/125 | 152/152 | 172/214 |
| 476 | M | 489 | 204/212 | 241/247 | 186/186 | 203/203 | 135/137 | 160/170 | 194/219 | 146/178 | 243/243 | 148/173 | 168/172 | 183/189 | 210/210 | 212/212 | 141/141 | 199/205 | 147/184 | 116/127 | 152/157 | 172/210 |
| 477 | M | 490 | 204/270 | 234/241 | 129/130 | 195/203 | 134/139 | 180/204 | 176/176 | 130/178 | 243/243 | 148/154 | 172/174 | 183/189 | 206/210 | 212/221 | 121/141 | 183/197 | 159/171 | 114/125 | 160/160 | 175/214 |
| 478 | M | 491 | 204/268 | 234/241 | 129/130 | 195/203 | 134/139 | 180/208 | 176/176 | 130/178 | 243/243 | 148/154 | 172/174 | 183/189 | 206/210 | 212/221 | 121/141 | 183/197 | 159/171 | 114/125 | 160/160 | 175/214 |
| 479 | M | 492 | 204/261 | 234/241 | 129/130 | 195/203 | 134/139 | 180/204 | 176/176 | 130/178 | 243/243 | 148/154 | 172/174 | 183/189 | 206/210 | 212/221 | 121/141 | 183/197 | 159/171 | 114/125 | 160/160 | 175/208 |
| 480 | M | 493 | 204/252 | 234/241 | 129/130 | 195/203 | 134/139 | 180/204 | 176/176 | 130/178 | 243/243 | 148/154 | 172/174 | 183/189 | 206/210 | 212/221 | 121/141 | 183/197 | 159/176 | 114/125 | 160/160 | 175/214 |
| 481 | M | 494 | 204/212 | 237/247 | 130/161 | 203/203 | 135/137 | 192/204 | 154/154 | 140/160 | 254/263 | 122/173 | 168/176 | 183/185 | 210/220 | 207/212 | 127/141 | 197/199 | 147/184 | 116/119 | 152/168 | 172/216 |
| 482 | M | 495 | 204/204 | 229/229 | 129/129 | 203/203 | 135/151 | 160/214 | 218/218 | 146/152 | 243/243 | 148/148 | 166/172 | 183/183 | 206/210 | 207/212 | 118/144 | 191/199 | 141/188 | 119/125 | 160/173 | 194/216 |
| 483 | M | 496 | 204/204 | 229/247 | 129/161 | 203/205 | 129/134 | 174/202 | 204/204 | 140/146 | 243/243 | 122/124 | 166/172 | 183/193 | 206/210 | 212/212 | 121/121 | 191/197 | 147/171 | 114/114 | 160/160 | 210/216 |
| 484 | M | 497 | 204/204 | 229/241 | 129/130 | 191/203 | 129/134 | 184/208 | 162/162 | 140/146 | 243/243 | 122/152 | 168/180 | 181/189 | 206/210 | 212/212 | 121/127 | 189/205 | 171/171 | 114/116 | 160/168 | 185/212 |
| 485 | M | 498 | 204/204 | 229/237 | 129/130 | 203/205 | 123/134 | 194/202 | 158/158 | 140/178 | 243/263 | 148/152 | 168/172 | 181/185 | 206/206 | 207/212 | 121/127 | 189/197 | 157/171 | 103/125 | 152/154 | 175/212 |
| 486 | M | 499 | 204/204 | 243/247 | 130/159 | 203/207 | 129/134 | 160/202 | 156/156 | 146/156 | 243/243 | 122/222 | 166/172 | 181/183 | 210/216 | 212/212 | 121/141 | 189/197 | 137/171 | 114/119 | 152/160 | 208/216 |
| 487 | M | 500 | 204/204 | 243/247 | 130/159 | 203/207 | 129/134 | 160/202 | 156/218 | 146/156 | 243/243 | 122/230 | 166/172 | 181/183 | 210/216 | 212/212 | 121/141 | 189/197 | 137/171 | 114/119 | 152/160 | 208/216 |
| 488 | M | 501 | 204/204 | 243/247 | 130/159 | 203/207 | 129/134 | 160/206 | 156/218 | 146/156 | 243/243 | 122/220 | 166/172 | 181/183 | 210/216 | 212/212 | 121/141 | 189/197 | 137/171 | 114/119 | 152/160 | 208/216 |
| 489 | M | 502 | 204/204 | 243/247 | 130/159 | 203/207 | 129/134 | 160/206 | 156/218 | 146/156 | 243/243 | 122/209 | 166/172 | 181/183 | 210/216 | 212/212 | 121/141 | 189/197 | 137/171 | 114/119 | 152/160 | 208/216 |
| 490 | M | 503 | 204/204 | 229/237 | 129/130 | 203/205 | 123/134 | 204/206 | 158/158 | 140/178 | 243/263 | 148/152 | 168/172 | 181/185 | 206/206 | 207/212 | 121/127 | 189/197 | 157/171 | 103/125 | 152/154 | 175/212 |
| 491 | M | 504 | 204/204 | 229/237 | 129/130 | 203/205 | 123/134 | 202/204 | 158/158 | 140/174 | 243/263 | 148/152 | 168/172 | 181/185 | 206/206 | 207/212 | 121/127 | 189/197 | 157/171 | 103/125 | 152/154 | 175/212 |
| 492 | M | 505 | 204/204 | 229/237 | 129/130 | 203/205 | 123/134 | 192/204 | 158/158 | 140/178 | 243/263 | 148/152 | 168/172 | 181/185 | 206/206 | 207/212 | 121/127 | 189/197 | 157/171 | 103/125 | 152/154 | 175/212 |
| 493 | M | 506 | 204/204 | 229/237 | 129/130 | 203/205 | 123/134 | 202/204 | 158/158 | 140/178 | 243/263 | 148/152 | 168/172 | 181/185 | 206/206 | 207/212 | 121/127 | 189/197 | 157/171 | 103/125 | 152/154 | 175/212 |
| 494 | M | 507 | 204/204 | 229/237 | 129/130 | 203/205 | 123/134 | 204/206 | 158/158 | 140/178 | 243/263 | 148/152 | 168/172 | 181/185 | 206/206 | 207/212 | 121/130 | 189/197 | 157/171 | 103/125 | 152/154 | 175/212 |
| 495 | M | 508 | 204/212 | 243/247 | 130/161 | 203/203 | 135/137 | 160/196 |  | 146/160 | 243/263 | 124/148 | 172/180 | 183/189 | 210/210 | 212/212 | 118/124 | 205/217 | 133/147 | 114/127 | 157/160 | 170/214 |
| 496 | M | 509 | 212/212 | 247/251 | 161/161 | 203/203 | 123/129 | 170/196 | 156/156 | 160/178 | 243/263 | 124/144 | 168/170 | 181/183 | 220/220 | 212/212 | 118/124 | 189/189 | 165/176 | 114/116 | 157/168 | 172/214 |
| 497 | M | 510 | 204/225 | 241/247 | 130/130 | 203/207 | 137/137 | 160/194 | 194/230 | 146/152 | 243/267 | 122/173 | 172/180 | 185/189 | 210/210 | 207/212 | 121/124 | 205/205 | 147/147 | 116/131 | 152/164 | 175/212 |
| 498 | M | 511 | 212/214 | 237/241 | 130/150 | 191/197 | 123/134 | 170/204 | 172/172 | 140/146 | 243/263 | 148/216 | 174/180 | 183/185 | 226/226 | 212/225 | 121/127 | 189/197 | 157/171 | 114/116 | 154/168 | 170/175 |
| 499 | M | 512 | 204/204 | 237/241 | 138/186 | 197/203 | 129/137 | 178/194 | 194/218 | 140/160 | 263/263 | 148/183 | 168/172 | 189/189 | 210/210 | 212/212 | 141/141 | 205/205 | 147/147 | 114/127 | 157/160 | 185/214 |
| 500 | M | 513 | 204/212 | 243/247 | 130/161 | 203/203 | 135/137 | 160/170 | 218/221 | 178/178 | 243/263 | 148/175 | 168/180 | 181/183 | 206/210 | 207/212 | 124/141 | 189/199 | 147/184 | 114/119 | 152/160 | 210/214 |
| 501 | M | 514 | 204/212 | 241/247 | 130/161 | 203/203 | 129/135 | 192/196 | 218/240 | 176/178 | 243/263 | 124/148 | 168/180 | 181/183 | 210/210 | 212/212 | 124/141 | 189/217 | 133/176 | 114/119 | 152/157 | 206/214 |
| 502 | M | 515 | 204/214 | 237/243 | 161/161 | 203/203 | 123/129 | 170/192 | 218/242 | 136/178 | 263/263 | 124/148 | 168/170 | 181/183 | 210/210 | 212/230 | 124/141 | 189/189 | 165/176 | 114/116 | 157/168 | 172/179 |
| 503 | M | 516 | 204/214 | 247/253 | 132/132 | 203/207 | 123/137 | 160/180 | 154/156 | 140/146 | 243/267 | 124/152 | 172/180 | 183/189 | 210/210 | 212/212 | 124/141 | 183/205 | 147/157 | 125/127 | 152/157 | 170/175 |
| 504 | M | 517 | 204/204 | 237/243 | 159/186 | 203/205 | 129/137 | 170/192 | 152/152 | 130/146 | 243/254 | 148/179 | 174/176 | 189/193 | 206/210 | 207/212 | 121/141 | 191/205 | 147/147 | 114/116 | 160/168 | 175/175 |
| 505 | M | 518 | 212/214 | 229/237 | 138/178 | 205/213 | 129/163 | 184/198 | 152/152 | 126/178 | 263/263 | 122/143 | 166/183 | 181/183 | 206/220 | 207/212 | 121/141 | 189/191 | 171/171 | 116/131 | 168/168 | 175/194 |
| 506 | M | 519 | 204/204 | 241/251 | 130/161 | 191/203 | 135/137 | 170/192 | 154/154 | 146/178 | 263/263 | 122/173 | 168/180 | 183/185 | 210/210 | 212/212 | 118/141 | 199/205 | 147/184 | 116/127 | 157/168 | 175/210 |
| 507 | M | 520 | 204/212 | 229/237 | 132/186 | 205/213 | 134/137 | 180/182 | 138/138 | 140/178 | 263/263 | 144/154 | 174/183 | 181/189 | 210/210 | 207/212 | 124/141 | 189/205 | 147/176 | 114/114 | 160/160 | 175/212 |
| 508 | M | 521 | 204/214 | 229/232 | 130/159 | 195/203 | 134/137 | 198/204 | 154/156 | 140/178 | 254/263 | 148/181 | 166/168 | 183/189 | 210/226 | 207/212 | 121/124 | 189/197 | 157/176 | 103/114 | 154/160 | 172/175 |
| 509 | M | 522 | 204/214 | 237/241 | 130/186 | 203/203 | 134/139 | 182/202 | 158/162 | 140/140 | 254/263 | 144/148 | 172/180 | 181/183 | 206/210 | 212/212 | 121/121 | 189/191 | 171/171 | 116/125 | 152/168 | 212/218 |
| 510 | M | 523 | 204/204 | 229/247 | 129/129 | 203/203 | 134/135 | 160/202 | 154/154 | 140/160 | 243/263 | 148/175 | 168/172 | 183/185 | 210/210 | 212/212 | 127/141 | 197/217 | 133/171 | 103/127 | 154/157 | 172/175 |
| 511 | M | 524 | 204/204 | 229/251 | 130/161 | 203/205 | 123/135 | 160/202 | 152/154 | 140/178 | 243/243 | 148/173 | 168/172 | 183/185 | 210/210 | 207/212 | 118/121 | 197/207 | 133/157 | 103/119 | 152/154 | 175/210 |
| 512 | M | 525 | 204/204 | 237/247 | 129/161 | 203/205 | 134/135 | 160/202 | 158/158 | 140/160 | 243/243 | 124/148 | 168/180 | 183/185 | 206/210 | 207/212 | 118/121 | 197/199 | 171/184 | 119/125 | 152/152 | 172/175 |
| 513 | M | 526 | 204/204 | 229/251 | 129/161 | 203/203 | 134/135 | 160/202 | 156/156 | 178/178 | 243/263 | 152/173 | 172/172 | 183/185 | 210/210 | 207/212 | 127/141 | 197/217 | 133/171 | 103/119 | 152/154 | 172/212 |
| 514 | M | 527 | 204/216 | 229/229 | 130/130 | 197/203 | 125/151 | 170/170 |  | 152/178 | 243/263 | 144/148 | 172/174 | 183/185 | 206/210 | 207/212 | 118/141 | 191/191 | 141/157 | 119/125 | 152/160 | 212/212 |
| 515 | M | 528 | 204/214 | 229/251 | 125/134 | 203/203 | 132/151 | 180/192 | 154/154 | 146/146 | 261/261 | 122/175 | 166/172 | 183/208 | 210/210 | 212/212 | 118/144 | 191/191 | 141/141 | 119/127 | 160/160 | 194/196 |
| 516 | M | 529 | 214/214 | 229/229 | 125/125 | 203/203 | 132/135 | 180/212 | 154/154 | 146/146 | 261/261 | 122/175 | 166/172 | 183/208 | 206/210 | 207/212 | 144/144 | 191/199 | 141/141 | 114/119 | 160/160 | 175/196 |
| 517 | M | 530 | 204/214 | 229/251 | 125/125 | 203/203 | 135/151 | 160/180 | 154/154 | 146/152 | 243/261 | 148/175 | 166/180 | 183/183 | 210/210 | 212/212 | 118/121 | 191/199 | 141/184 | 119/125 | 160/173 | 194/216 |
| 518 | M | 531 | 204/214 | 229/229 | 129/129 | 203/203 | 135/151 | 192/218 | 154/154 | 146/152 | 243/261 | 122/148 | 172/172 | 183/183 | 206/210 | 207/212 | 144/144 | 191/199 | 141/182 | 114/125 | 160/173 | 175/194 |
| 519 | M | 532 | 204/212 | 229/237 | 130/130 | 197/203 | 134/151 | 170/214 | 170/170 | 146/178 | 261/263 | 122/148 | 170/172 | 183/185 | 206/210 | 210/212 | 141/144 | 191/197 | 141/174 | 125/131 | 157/173 | 166/194 |
| 520 | M | 533 | 204/212 | 237/251 | 130/130 | 191/203 | 125/135 | 170/204 | 156/156 | 140/178 | 254/263 | 144/148 | 170/176 | 183/185 | 210/210 | 212/212 | 141/141 | 197/217 |  |  | 154/157 | 172/212 |
| 521 | M | 534 | 204/214 | 229/251 | 125/125 | 203/203 | 135/151 | 160/204 | 166/166 | 146/160 | 243/261 | 122/175 | 166/170 | 183/183 | 210/235 | 207/212 | 118/135 | 191/199 | 141/184 | 125/127 | 157/173 | 194/196 |
| 522 | M | 535 | 204/216 | 229/229 | 186/186 | 197/203 | 125/135 | 160/170 | 156/230 | 140/152 | 261/263 | 122/148 | 166/170 | 183/183 | 206/206 | 210/212 | 121/144 | 191/199 | 157/188 | 119/131 | 160/160 | 166/216 |
| 523 | M | 536 | 204/204 | 229/229 | 161/161 | 203/203 | 129/135 | 172/192 | 244/244 | 146/146 | 243/261 | 126/152 | 172/178 | 181/181 | 206/210 | 212/212 | 124/144 | 189/199 | 176/184 | 119/127 | 152/160 | 170/194 |
| 524 | M | 537 | 204/212 | 237/251 | 130/186 | 191/203 | 125/137 | 160/170 | 218/230 | 160/178 | 263/263 | 144/152 | 174/176 | 183/185 | 210/210 | 210/212 | 121/141 | 191/197 | 133/157 | 125/134 | 154/154 | 212/212 |
| 525 | M | 538 | 204/214 | 229/229 | 130/161 | 203/203 | 129/134 | 160/172 | 218/218 | 140/142 | 263/263 | 122/122 | 174/174 | 183/185 | 210/216 | 212/212 | 118/135 | 189/217 | 155/171 | 119/125 | 152/160 | 175/210 |
| 526 | M | 539 | 204/214 | 229/229 | 130/130 | 203/203 | 129/134 | 160/192 | 218/244 | 142/146 | 243/263 | 122/152 | 174/178 | 181/183 | 210/210 | 212/212 | 135/144 | 189/217 | 155/176 | 125/127 | 160/160 | 175/194 |
| 527 | M | 540 | 214/214 | 229/255 | 129/161 | 203/209 | 125/151 | 192/214 | 156/218 | 146/178 | 243/261 | 122/143 | 168/172 | 183/183 | 206/210 | 207/219 | 121/144 | 191/191 | 141/157 | 125/131 | 154/173 | 166/175 |
| 528 | M | 541 | 204/204 | 251/251 | 161/161 | 195/203 | 135/151 | 172/218 | 166/166 | 142/146 | 243/261 | 122/179 | 174/174 | 181/181 | 206/210 | 212/212 | 124/141 | 189/203 | 133/184 | 114/119 | 152/160 | 187/210 |
| 529 | M | 542 | 204/212 | 243/247 | 130/161 | 203/203 | 135/137 | 160/196 | 218/238 | 146/160 | 243/263 | 122/173 | 172/180 | 183/189 | 210/210 | 212/212 | 118/124 | 205/217 | 133/147 | 114/127 | 157/160 | 172/216 |
| 530 | M | 543 | 204/204 | 247/247 | 116/129 | 193/203 | 129/134 | 192/204 | 154/154 | 160/160 | 243/243 | 124/203 | 178/178 | 181/185 | 210/216 | 212/212 | 118/121 | 189/203 | 145/147 | 116/119 | 152/152 | 175/208 |
| 531 | M | 544 | 204/204 | 229/251 | 129/130 | 203/203 | 129/137 | 170/204 | 152/152 | 146/160 | 243/243 | 122/124 | 172/176 | 185/193 | 210/216 | 212/212 | 121/141 | 191/205 | 147/147 | 116/127 | 157/168 | 175/214 |
| 532 | M | 545 | 204/212 | 243/247 | 130/161 | 203/203 | 135/139 | 160/182 | 156/156 | 140/160 | 254/263 | 124/152 | 168/176 | 183/183 | 210/220 | 207/212 | 127/141 | 191/217 | 133/133 | 116/127 | 157/168 | 212/212 |
| 533 | M | 546 | 204/212 | 237/247 | 130/130 | 203/203 | 135/139 | 160/182 | 154/154 | 160/178 | 243/263 | 122/175 | 168/176 | 183/183 | 210/220 | 207/212 | 118/121 | 191/217 | 133/133 | 119/134 | 152/154 | 204/216 |
| 534 | M | 547 | 204/212 | 241/247 | 186/186 | 203/203 | 134/135 | 160/170 | 154/154 | 146/160 | 243/263 | 122/124 | 168/172 | 183/185 | 210/210 | 212/212 | 118/141 | 197/217 | 133/171 | 116/119 | 152/168 | 202/208 |
| 535 | M | 548 | 204/204 | 243/247 | 129/165 | 191/205 | 129/137 | 160/202 | 152/152 | 140/178 | 243/243 | 122/148 | 166/180 | 181/183 | 206/210 | 212/212 | 121/141 | 189/217 | 147/171 | 114/127 | 157/160 | 175/214 |
| 536 | M | 549 | 204/204 | 237/243 | 129/161 | 203/205 | 129/135 | 170/192 | 156/194 | 140/140 | 243/243 | 122/124 | 166/174 | 181/183 | 210/216 | 212/212 | 124/127 | 189/217 | 133/171 | 127/127 | 157/160 | 175/198 |
| 537 | M | 550 | 204/204 | 241/243 | 159/186 | 203/203 | 129/137 | 192/204 | 152/152 | 146/178 | 243/243 | 148/152 | 166/174 | 181/189 | 210/216 | 212/212 | 124/127 | 189/205 | 171/171 | 114/125 | 152/160 | 216/221 |
| 538 | M | 551 | 212/229 | 237/241 | 129/152 | 191/191 | 129/145 | 180/206 | 194/194 | 146/146 | 263/263 | 122/148 | 166/168 | 183/185 | 206/206 | 212/212 | 121/121 | 191/205 | 147/171 | 123/125 | 152/152 | 214/214 |
| 539 | M | 552 | 204/212 | 241/241 | 129/186 | 205/211 | 135/137 | 164/184 | 156/156 | 130/130 | 267/267 | 122/148 | 158/172 | 183/183 | 220/220 | 212/212 | 121/124 | 183/217 | 133/147 | 116/131 | 154/168 | 202/202 |
| 540 | M | 553 | 212/240 | 237/241 | 129/152 | 191/191 | 129/147 | 180/206 | 194/242 | 146/146 | 263/263 | 122/148 | 166/168 | 183/185 | 206/206 | 212/212 | 121/121 | 191/205 | 147/171 | 123/125 | 152/152 | 214/216 |
| 541 | M | 554 | 204/261 | 241/247 | 129/129 | 203/205 | 134/135 | 184/192 | 156/218 | 130/174 | 243/267 | 173/173 | 158/180 | 183/183 | 210/220 | 212/230 | 121/141 | 197/217 | 133/171 | 119/131 | 152/154 | 172/175 |
| 542 | M | 555 | 204/204 | 243/247 | 129/129 | 203/205 | 129/137 | 160/202 | 152/152 | 140/146 | 243/243 | 148/152 | 172/172 | 181/189 | 210/216 | 212/212 | 124/141 | 189/205 | 147/163 | 114/116 | 152/160 | 212/212 |
| 543 | M | 556 | 204/268 | 241/251 | 129/129 | 195/203 | 129/139 | 180/192 | 194/194 | 140/178 | 243/263 | 124/148 | 168/174 | 183/193 | 206/216 | 212/212 | 121/141 | 183/191 | 147/161 | 114/119 | 152/160 | 175/175 |
| 544 | M | 557 | 212/268 | 241/241 | 132/190 | 195/203 | 139/145 | 204/204 | 162/176 | 140/178 | 243/243 | 144/148 | 174/180 | 183/183 | 210/210 | 212/212 | 121/127 | 183/191 | 157/157 | 112/116 | 168/168 | 175/175 |
| 545 | M | 558 | 204/214 | 229/251 | 130/130 | 199/203 | 135/151 | 160/192 | 168/168 | 146/178 | 243/254 | 124/173 | 174/180 | 183/189 | 210/224 | 207/207 | 118/141 | 183/217 | 133/155 | 116/127 | 154/168 | 172/210 |
| 546 | M | 559 | 204/204 | 237/251 | 138/138 | 191/209 | 135/137 | 164/192 | 166/166 | 140/160 | 263/263 | 173/173 | 172/174 | 183/183 | 210/220 | 212/252 | 118/121 | 193/199 | 147/184 | 127/131 | 152/157 | 170/210 |
| 547 | M | 560 | 204/204 | 229/247 | 129/129 | 203/203 | 134/135 | 190/204 | 152/152 | 146/160 | 243/243 | 122/173 | 168/172 | 183/189 | 210/210 | 207/212 | 127/141 | 205/217 | 133/171 | 114/119 | 152/160 | 175/210 |
| 548 | M | 561 | 204/204 | 237/247 | 161/161 | 203/203 | 137/145 | 160/160 | 152/178 | 140/166 | 243/263 | 173/173 | 172/176 | 181/181 | 206/220 | 207/212 | 121/124 | 183/189 | 133/194 | 114/131 | 160/160 | 170/172 |
| 549 | M | 562 | 204/204 | 229/247 | 132/132 | 201/203 | 135/137 | 160/184 | 152/152 | 140/178 | 243/263 | 144/173 | 170/172 | 183/183 | 210/220 | 207/212 | 124/141 | 191/217 | 133/155 | 114/127 | 154/157 | 172/175 |
| 550 | M | 563 | 204/214 | 229/243 | 129/161 | 195/203 | 135/155 | 202/204 | 162/162 | 126/178 | 243/243 | 179/230 | 166/170 | 183/183 | 206/220 | 207/212 | 124/141 | 193/193 | 155/184 | 114/131 | 154/154 | 177/177 |
| 551 | M | 564 | 204/214 | 229/241 | 132/192 | 203/211 | 129/153 | 170/184 | 166/166 | 140/160 | 254/263 | 148/165 | 166/172 | 181/181 | 206/220 | 212/212 | 118/124 | 189/191 | 157/171 | 125/131 | 152/154 | 172/196 |
| 552 | M | 565 | 212/219 | 229/241 | 159/184 | 199/203 | 135/153 | 160/204 | 194/218 | 130/146 | 243/254 | 159/173 | 168/170 | 183/183 | 210/212 | 212/212 | 127/141 | 189/199 | 157/184 | 116/131 | 152/164 | 172/175 |
| 553 | M | 566 | 204/232 | 229/229 | 129/132 | 197/203 | 127/134 | 200/200 | 156/156 | 134/146 | 243/267 | 122/152 | 168/170 | 183/189 | 220/220 | 212/256 | 121/121 | 189/205 | 155/171 | 107/114 | 160/164 | 175/185 |
| 554 | M | 567 | 204/240 | 241/251 | 130/186 | 195/203 | 135/139 | 184/204 | 176/194 | 130/134 | 243/243 | 148/171 | 172/176 | 183/183 | 206/216 | 207/221 | 121/141 | 183/183 | 133/159 | 114/119 | 152/160 | 175/198 |
| 555 | M | 568 | 204/212 | 241/241 | 192/192 | 203/203 | 129/137 | 160/184 | 194/218 | 130/160 | 254/263 | 148/173 | 166/183 | 181/189 | 206/210 | 212/212 | 118/124 | 189/205 | 147/171 | 116/125 | 152/152 | 172/214 |
| 556 | M | 569 | 204/204 | 241/241 | 130/130 | 203/203 | 134/145 | 160/204 | 176/194 | 140/178 | 254/263 | 144/154 | 172/174 | 183/189 | 210/210 | 212/212 | 121/141 | 189/197 | 157/171 | 125/127 | 152/157 | 170/214 |
| 557 | M | 570 | 212/212 | 234/243 | 130/163 | 197/205 | 123/129 | 182/184 | 152/152 | 140/178 | 243/243 | 122/144 | 172/172 | 181/183 | 206/210 | 207/212 | 121/124 | 189/193 | 157/171 | 114/134 | 154/160 | 185/216 |
| 558 | M | 571 | 204/212 | 241/243 | 130/130 | 203/203 | 129/137 | 170/184 | 152/152 | 140/146 | 243/243 | 144/148 | 166/168 | 189/193 | 216/216 | 212/240 | 124/127 | 191/205 | 143/147 | 125/140 | 152/152 | 214/216 |
| 559 | M | 572 | 204/214 | 229/243 | 129/186 | 205/209 | 129/151 | 180/184 | 146/146 | 130/146 | 243/263 | 152/173 | 172/180 | 181/185 | 206/210 | 212/212 | 124/141 | 183/191 | 147/157 | 121/125 | 152/160 | 179/179 |
| 560 | M | 573 | 204/212 | 241/247 | 159/161 | 195/203 | 129/137 | 204/204 | 194/242 | 140/178 | 243/263 | 144/152 | 166/176 | 183/193 | 216/216 | 212/212 | 127/127 | 191/191 | 147/155 | 119/131 | 157/157 | 214/214 |
| 561 | M | 574 | 212/265 | 229/241 | 129/186 | 191/211 | 125/134 | 170/170 | 156/156 | 130/182 | 263/263 | 148/152 | 172/174 | 181/185 | 206/206 | 207/212 | 121/127 | 189/197 | 155/171 | 116/131 | 154/168 | 175/214 |
| 562 | M | 575 | 204/204 | 243/251 | 129/161 | 203/205 | 129/135 | 160/202 | 154/154 | 140/160 | 243/263 | 122/126 | 166/172 | 183/185 | 210/220 | 207/212 | 118/127 | 191/199 | 147/184 | 119/134 | 152/154 | 172/175 |
| 563 | M | 576 | 204/212 | 243/247 | 129/186 | 203/207 | 135/139 | 192/204 | 162/182 | 140/178 | 243/254 | 124/143 | 168/170 | 183/183 | 212/220 | 212/212 | 118/121 | 183/199 | 184/184 | 112/119 | 154/154 | 210/210 |
| 564 | M | 577 | 204/214 | 234/247 | 129/129 | 203/203 | 135/141 | 182/192 | 154/162 | 140/160 | 243/254 | 124/143 | 170/172 | 183/183 | 210/212 | 212/212 | 118/121 | 191/199 | 157/184 | 112/127 | 154/157 | 170/210 |
| 565 | M | 578 | 204/214 | 234/241 | 130/186 | 203/211 | 137/147 | 182/198 | 162/164 | 140/178 | 243/263 | 148/159 | 162/180 | 189/189 | 210/226 | 212/225 | 121/124 | 191/205 | 147/157 | 103/125 | 152/154 | 175/212 |
| 566 | M | 579 | 204/204 | 241/251 | 161/190 | 203/203 | 129/135 | 160/170 | 154/154 | 140/180 | 243/263 | 122/124 | 166/183 | 181/183 | 210/210 | 212/212 | 124/141 | 189/199 | 171/184 | 119/125 | 152/152 | 210/214 |
| 567 | M | 580 | 204/214 | 229/241 | 129/130 | 203/205 | 134/137 | 182/194 | 162/162 | 140/178 | 243/263 | 144/152 | 168/174 | 185/189 | 210/210 | 212/212 | 121/127 | 197/205 | 147/176 | 103/116 | 154/168 | 175/212 |
| 568 | M | 581 | 204/212 | 229/243 | 129/165 | 203/205 | 129/129 | 192/202 | 152/152 | 140/146 | 243/243 | 122/152 | 166/174 | 181/193 | 206/216 | 212/212 | 121/124 | 189/191 | 147/161 | 114/127 | 160/160 | 175/218 |
| 569 | M | 582 | 212/268 | 229/241 | 130/186 | 195/203 | 139/139 | 180/202 | 162/176 | 140/178 | 254/263 | 144/148 | 172/180 | 183/189 | 210/220 | 212/221 | 121/141 | 183/205 | 159/159 | 116/125 | 152/168 | 175/212 |
| 570 | M | 583 | 212/274 | 234/251 | 130/130 | 195/203 | 134/145 | 180/204 | 154/154 | 140/178 | 254/263 | 144/148 | 172/174 | 183/183 | 206/210 | 212/221 | 121/141 | 197/199 | 157/171 | 114/134 | 154/160 | 175/210 |
| 571 | M | 584 | 204/212 | 237/241 | 130/186 | 203/203 | 123/137 | 202/204 | 162/162 | 140/140 | 243/263 | 144/148 | 168/174 | 181/183 | 206/220 | 212/212 | 127/127 | 189/191 | 147/157 | 125/134 | 152/154 | 210/216 |
| 572 | M | 585 | 214/216 | 237/241 | 159/186 | 203/203 | 139/143 | 180/182 | 162/162 | 130/178 | 243/263 | 122/124 | 170/174 | 183/189 | 220/222 |  | 121/124 | 183/191 | 157/157 | 116/116 | 160/168 | 175/212 |
| 573 | M | 586 | 214/268 | 234/237 | 129/129 | 191/195 | 134/139 | 170/180 | 154/154 | 158/182 | 243/263 | 124/148 | 158/174 | 183/189 | 210/210 | 207/212 | 121/141 | 183/197 | 157/174 | 114/131 | 154/160 | 175/175 |
| 574 | M | 587 | 204/212 | 237/251 | 129/129 | 203/203 | 135/157 | 160/202 | 154/162 | 146/160 | 243/243 | 124/179 | 168/172 | 181/183 | 206/210 | 212/212 | 118/124 | 193/199 | 155/184 | 119/131 | 152/154 | 177/210 |
| 575 | M | 588 | 204/204 | 241/247 | 159/159 | 203/203 | 129/135 | 160/170 | 152/154 | 164/176 | 243/243 | 122/124 | 168/172 | 181/183 | 206/210 | 212/212 | 124/141 | 189/199 | 171/184 | 119/125 | 152/152 | 212/216 |
| 576 | M | 589 | 204/214 | 241/253 | 138/186 | 195/203 | 134/147 | 170/170 | 168/168 | 134/178 | 243/267 | 143/148 | 154/168 | 189/189 | 210/210 | 207/212 | 121/141 | 183/197 | 151/157 | 116/131 | 152/154 | 175/175 |
| 577 | M | 590 | 212/214 | 229/229 | 129/134 | 197/203 | 139/155 | 180/204 | 162/162 | 140/178 | 243/263 | 144/163 | 172/174 | 183/183 | 210/226 | 207/212 | 121/124 | 191/191 | 157/157 | 131/131 | 154/160 | 187/216 |
| 578 | M | 591 | 204/204 | 229/251 | 130/161 | 203/205 | 123/135 | 160/204 | 154/158 | 160/178 | 263/263 | 124/152 | 168/172 | 181/183 | 206/210 | 207/212 | 127/141 | 189/199 | 157/184 | 103/127 | 154/157 | 210/210 |
| 579 | M | 592 | 212/268 | 234/237 | 130/130 | 195/203 | 137/139 | 180/192 | 154/154 | 178/178 | 243/263 | 148/152 | 166/172 | 185/189 | 210/210 | 212/221 | 121/121 | 183/197 | 147/159 | 114/119 | 152/160 | 212/214 |
| 580 | M | 593 | 204/214 | 229/237 | 129/138 | 191/197 | 123/134 | 164/200 | 152/160 | 140/146 | 243/243 | 122/143 | 172/183 | 183/189 | 220/220 | 207/212 | 121/124 | 189/205 | 171/171 | 125/131 | 154/168 | 175/179 |
| 581 | M | 594 | 204/212 | 241/251 | 129/129 | 203/203 | 134/145 | 160/204 | 154/176 | 140/178 | 254/263 | 144/154 | 174/174 | 183/183 | 206/210 | 212/221 | 141/141 | 189/197 | 157/171 | 125/127 | 152/157 | 170/175 |
| 582 | M | 595 | 204/268 | 229/237 | 148/186 | 191/195 | 134/159 | 182/200 | 152/156 | 126/146 | 243/254 | 143/152 | 168/172 | 189/189 | 216/220 | 210/212 | 121/121 | 183/205 | 157/171 | 114/131 | 160/160 | 172/175 |
| 583 | M | 596 | 204/204 | 241/241 | 129/129 | 203/203 | 135/139 | 160/204 | 194/194 | 140/178 | 254/263 | 148/173 | 172/174 | 183/183 | 206/210 | 212/221 | 121/141 | 183/199 | 159/184 | 125/134 | 154/154 | 170/214 |
| 584 | M | 597 | 204/204 | 241/251 | 129/130 | 203/203 | 135/139 | 160/204 | 154/176 | 140/178 | 243/254 | 144/154 | 172/174 | 183/183 | 210/210 | 212/221 | 121/141 | 183/199 | 159/184 | 114/134 | 154/160 | 170/214 |
| 585 | M | 598 | 204/212 | 229/241 | 130/186 | 197/203 | 134/137 | 160/170 | 170/218 | 140/178 | 243/263 | 122/173 | 170/172 | 183/185 | 210/210 | 212/212 | 121/141 | 197/205 | 147/174 | 125/127 | 152/157 | 212/212 |
| 586 | M | 599 | 212/212 | 237/237 | 146/186 | 191/205 | 129/134 | 170/192 | 166/166 | 140/178 | 254/263 | 122/169 | 168/172 | 181/185 | 206/210 | 212/212 | 121/127 | 191/197 | 147/171 | 114/116 | 160/168 | 170/175 |
| 587 | M | 600 | 204/204 | 237/237 | 129/130 | 203/205 | 123/134 | 202/208 | 158/158 | 140/140 | 243/263 | 148/152 | 168/172 | 181/185 | 206/206 | 207/212 | 121/127 | 189/197 | 157/171 | 103/125 | 152/154 | 175/210 |
| 588 | M | 601 | 204/212 | 241/241 | 129/138 | 195/203 | 134/137 | 160/170 | 160/168 | 134/156 | 243/263 | 124/143 | 154/154 | 183/183 | 206/210 | 207/212 | 141/141 | 197/209 | 157/171 | 131/131 | 154/154 | 175/175 |
| 589 | M | 602 | 204/212 | 229/247 | 129/129 | 203/203 | 135/137 | 182/202 | 154/162 | 140/178 | 243/243 | 144/173 | 174/180 | 183/189 | 210/220 | 207/212 | 118/121 | 205/217 | 133/147 | 119/134 | 152/154 | 175/175 |
| 590 | M | 603 | 204/265 | 241/247 | 161/190 | 195/203 | 134/135 | 180/204 | 156/156 | 134/134 | 243/263 | 144/152 | 166/174 | 183/189 | 210/210 | 212/212 | 124/127 | 197/217 | 174/186 | 119/125 | 152/157 | 175/214 |
| 591 | M | 604 | 204/214 | 239/247 | 142/161 | 199/203 | 135/135 | 160/184 | 154/154 | 126/160 | 263/263 | 124/163 | 166/172 | 183/193 | 206/210 |  | 121/144 | 191/217 | 133/157 | 114/127 | 152/157 | 187/210 |
| 592 | M | 605 | 204/265 | 229/237 | 186/186 | 203/205 | 125/137 | 170/172 | 194/194 | 140/160 | 254/263 | 169/173 | 168/172 | 183/183 | 210/210 | 212/212 | 121/127 | 191/199 | 147/147 | 125/131 | 152/164 | 208/212 |
| 593 | M | 606 | 204/204 | 241/247 | 129/161 | 203/203 | 134/134 | 180/206 | 176/218 | 130/160 | 243/263 | 124/154 | 160/174 | 183/189 | 206/210 | 212/212 | 121/141 | 197/199 | 171/171 | 103/114 | 154/160 | 175/175 |
| 594 | M | 607 | 204/214 | 229/251 | 129/132 | 195/205 | 123/135 | 160/178 | 154/178 | 130/178 | 254/263 | 148/175 | 172/178 | 183/183 | 210/220 | 207/256 | 124/124 | 191/199 | 157/184 | 114/116 | 168/168 | 175/175 |
| 595 | M | 608 | 204/212 | 241/247 | 161/188 | 203/203 | 135/137 | 160/170 | 152/152 | 140/160 | 243/263 | 148/173 | 166/176 | 181/183 | 206/210 | 207/212 | 118/124 | 189/199 | 133/169 | 114/127 | 157/160 | 172/214 |
| 596 | M | 609 | 204/240 | 237/251 | 161/190 | 203/203 | 132/135 | 182/194 | 176/176 | 134/174 | 243/267 | 124/171 | 172/176 | 183/183 | 210/216 | 207/207 | 124/141 | 183/217 | 133/157 | 119/131 | 152/154 | 198/210 |
| 597 | M | 610 | 204/214 | 237/251 | 129/161 | 191/195 | 134/155 | 160/180 | 154/162 | 146/146 | 243/243 | 122/179 | 170/176 | 183/189 | 206/210 | 207/212 | 118/124 | 193/205 | 147/155 | 114/131 | 154/160 | 177/177 |
| 598 | M | 611 | 204/268 | 237/237 | 138/138 | 203/209 | 135/139 | 174/192 | 164/164 | 140/140 | 243/263 | 138/152 | 162/176 | 183/185 | 206/220 | 207/212 | 118/124 | 191/197 | 133/159 | 131/134 | 154/164 | 172/172 |
| 599 | M | 612 | 204/214 | 237/237 | 130/138 | 203/209 | 135/143 | 182/192 | 154/154 | 140/160 | 243/263 | 173/189 | 168/183 | 183/183 | 220/226 | 212/250 | 118/121 | 193/217 | 133/159 | 131/134 | 154/164 | 172/187 |
| 600 | M | 613 | 204/214 | 243/251 | 130/161 | 203/203 | 135/137 | 160/182 | 154/154 | 140/160 | 254/263 | 122/124 | 166/172 | 183/185 | 210/220 | 207/212 | 118/124 | 197/199 | 147/184 | 116/119 | 152/168 | 172/216 |
| 601 | M | 614 | 204/214 | 227/229 | 129/130 | 203/203 | 123/129 | 182/204 | 162/162 | 130/178 | 263/263 | 144/183 | 172/176 | 183/183 | 220/220 | 207/212 | 121/127 | 183/189 | 171/174 | 114/125 | 152/154 | 175/185 |
| 602 | M | 615 | 204/216 | 237/237 | 150/150 | 203/211 | 137/149 | 186/204 | 138/154 | 140/140 | 254/267 | 144/152 | 168/183 | 183/185 | 210/220 | 207/212 | 121/127 | 183/197 | 147/157 | 112/127 | 152/164 | 172/179 |
| 603 | M | 616 | 212/227 | 237/251 | 130/138 | 199/203 | 123/135 | 184/204 | 138/154 | 126/160 | 243/254 | 152/187 | 162/176 | 183/183 | 210/220 | 212/242 | 124/127 | 183/217 | 133/157 | 134/140 | 154/154 | 212/212 |
| 604 | M | 617 | 204/212 | 251/251 | 161/165 | 203/211 | 135/139 | 192/204 | 160/216 | 140/140 | 243/263 | 122/152 | 162/172 | 183/183 | 210/220 | 212/212 | 127/141 | 191/193 | 157/157 | 116/127 | 157/168 | 170/208 |
| 605 | M | 618 | 204/212 | 237/247 | 130/130 | 203/203 | 135/139 | 160/182 | 154/154 | 160/174 | 263/263 | 124/152 | 168/172 | 183/183 | 210/220 | 212/212 | 127/141 | 191/199 | 176/178 | 116/119 | 152/168 | 172/216 |
| 606 | M | 619 | 204/212 | 237/251 | 174/174 | 203/203 | 135/137 | 160/204 | 216/242 | 146/160 | 243/263 | 148/173 | 168/172 | 183/189 | 210/210 | 207/212 | 127/141 | 205/219 | 133/147 | 116/119 | 152/168 | 172/212 |
| 607 | M | 620 | 204/214 | 237/251 | 130/130 | 203/203 | 135/137 | 192/204 | 194/219 | 140/160 | 254/263 | 152/173 | 168/172 | 183/185 | 210/210 | 207/212 | 127/141 | 197/217 | 147/184 | 127/134 | 157/168 | 208/216 |
| 608 | M | 621 | 204/212 | 243/251 | 130/161 | 203/203 | 135/137 | 160/182 | 154/154 | 160/174 | 243/263 | 124/152 | 168/172 | 183/185 | 210/210 | 207/212 | 121/141 | 197/217 | 133/147 | 119/134 | 152/154 | 208/212 |
| 609 | M | 622 | 204/212 | 243/251 | 130/161 | 203/203 | 135/137 | 192/204 | 194/218 | 178/178 | 243/263 | 122/124 | 168/176 | 183/185 | 210/210 | 212/212 | 124/141 | 197/199 | 147/184 | 127/134 | 154/157 | 172/216 |
| 610 | M | 623 | 204/204 | 229/251 | 129/150 | 203/205 | 134/135 | 160/202 | 154/158 | 140/178 | 243/263 | 124/152 | 172/180 | 183/185 | 210/210 | 212/212 | 118/121 | 197/217 | 133/171 | 103/119 | 152/154 | 172/175 |
| 611 | M | 624 | 204/204 | 234/247 | 129/161 | 203/203 | 135/139 | 192/202 | 154/158 | 140/178 | 243/243 | 124/154 | 172/174 | 183/185 | 206/206 | 212/212 | 121/127 | 183/199 | 159/171 | 119/127 | 152/157 | 175/210 |
| 612 | M | 625 | 204/212 | 243/247 | 152/152 | 203/203 | 125/129 | 170/186 | 156/228 | 146/156 | 243/263 | 124/177 | 172/185 | 181/198 | 210/210 | 207/212 | 124/141 | 189/195 | 147/153 | 114/127 | 152/160 | 172/208 |
| 613 | M | 626 | 204/204 | 243/247 | 161/161 | 203/203 | 129/135 | 160/174 | 154/154 | 148/160 | 243/263 | 124/173 | 172/172 | 183/193 | 210/210 | 207/212 | 118/124 | 191/217 | 133/147 | 114/119 | 152/160 | 172/216 |
| 614 | M | 627 | 204/240 | 234/243 | 130/198 | 201/203 | 134/135 | 170/190 | 156/216 | 146/162 | 243/243 | 173/199 | 172/172 | 183/187 | 210/216 | 212/212 | 118/121 | 197/205 | 137/141 | 114/114 | 160/160 | 170/216 |
| 615 | M | 628 | 204/212 | 237/243 | 130/138 | 199/203 | 134/139 | 160/170 | 146/146 | 140/146 | 243/254 | 144/161 | 172/174 | 183/185 | 210/210 | 207/212 | 124/141 | 191/197 | 155/171 | 103/131 | 154/154 | 172/185 |
| 616 | M | 629 | 204/206 | 234/247 | 152/163 | 197/203 | 137/141 | 174/192 | 154/154 | 146/182 | 243/243 | 154/177 | 170/185 | 183/198 | 210/239 | 212/212 | 118/147 | 199/205 | 137/141 | 119/127 | 152/152 | 172/210 |
| 617 | M | 630 | 204/206 | 249/249 | 129/194 | 201/207 | 129/137 | 170/192 | 154/154 | 140/162 | 243/263 | 173/173 | 172/172 | 181/183 | 206/216 | 212/212 | 121/124 | 191/199 | 147/147 | 116/119 | 152/160 | 170/175 |
| 618 | M | 631 | 214/214 | 237/247 | 138/148 | 199/203 | 134/139 | 160/170 | 154/154 | 140/140 | 254/254 | 144/161 | 162/164 | 183/183 | 210/210 | 207/212 | 121/124 | 197/217 | 155/171 | 119/125 | 152/152 | 172/185 |
| 619 | M | 632 | 204/265 | 229/237 | 186/186 | 203/205 | 125/135 | 170/172 | 194/194 | 140/160 | 254/263 | 169/173 | 168/172 | 183/183 | 210/210 | 212/212 | 121/127 | 191/199 | 147/147 | 125/131 | 152/164 | 208/212 |
| 620 | M | 633 | 204/212 | 234/251 | 129/130 | 203/203 | 129/134 | 160/204 | 148/194 | 130/178 | 243/263 | 144/154 | 172/174 | 189/193 | 206/210 | 212/221 | 118/121 | 191/197 | 157/171 | 114/125 | 152/160 | 175/175 |
| 621 | M | 634 | 204/212 | 247/251 | 159/159 | 203/203 | 134/137 | 170/186 | 156/228 | 160/160 | 243/263 | 124/177 | 172/178 | 183/198 | 210/210 | 212/212 | 124/141 | 197/205 | 137/171 | 119/127 | 160/168 | 175/216 |
| 622 | M | 635 | 204/214 | 237/247 | 148/161 | 199/203 | 135/151 | 160/170 | 154/162 | 140/178 | 243/254 | 124/148 | 162/180 | 183/185 | 210/210 | 207/207 | 141/141 | 183/199 | 157/184 | 103/119 | 152/154 | 185/210 |
| 623 | M | 636 | 204/212 | 229/247 | 129/159 | 203/205 | 134/134 | 174/192 | 152/152 | 178/178 | 243/243 | 124/152 | 172/172 | 183/189 | 210/216 | 212/212 | 121/124 | 197/209 | 133/171 | 119/127 | 152/160 | 175/216 |
| 624 | M | 637 | 204/212 | 243/251 | 159/159 | 203/203 | 129/137 | 170/186 | 156/228 | 160/160 | 263/263 | 124/177 | 170/172 | 181/198 | 210/210 | 207/212 | 118/124 | 189/205 | 137/147 | 114/127 | 152/160 | 175/210 |
| 625 | M | 638 | 204/240 | 234/243 | 163/198 | 201/203 | 129/135 | 192/196 | 226/263 | 162/178 | 243/263 | 124/124 | 172/185 | 183/187 | 210/210 | 212/212 | 118/124 | 191/205 | 141/147 | 114/119 | 152/160 | 170/218 |
| 626 | M | 639 | 204/204 | 234/247 | 159/163 | 197/203 | 135/137 | 174/186 | 154/154 | 160/178 | 243/263 | 154/177 | 168/170 | 183/198 | 210/210 | 212/212 | 118/147 | 199/205 | 137/184 | 119/127 | 152/160 | 172/210 |
| 627 | M | 640 | 204/204 | 251/251 | 129/129 | 203/205 | 125/129 | 174/202 | 152/152 | 146/146 | 261/263 | 124/177 | 170/178 | 181/193 | 210/210 | 212/212 | 118/118 | 191/195 | 153/171 | 119/127 | 152/152 | 175/223 |
| 628 | M | 641 | 204/240 | 247/255 | 116/159 | 203/205 | 125/129 | 170/204 | 194/232 | 160/178 | 243/263 | 122/228 | 172/174 | 181/181 | 210/210 | 207/219 | 118/121 | 189/189 | 161/171 | 114/119 | 152/160 | 170/210 |
| 629 | M | 642 | 204/204 | 237/243 | 159/159 | 191/203 | 134/134 | 170/192 | 152/156 | 146/160 | 243/263 | 122/124 | 172/174 | 183/183 | 206/212 | 212/225 | 124/124 | 191/197 | 155/171 | 116/119 | 168/168 | 214/216 |
| 630 | M | 643 | 204/206 | 234/249 | 163/198 | 201/207 | 137/137 | 170/196 | 216/226 | 162/162 | 243/263 | 124/124 | 172/189 | 183/196 | 210/210 | 212/212 | 124/124 | 199/217 | 147/171 | 116/119 | 152/160 | 170/216 |
| 631 | M | 644 | 204/212 | 237/251 | 130/161 | 203/203 | 135/137 | 182/192 | 194/218 | 140/160 | 243/254 | 152/173 | 168/176 | 183/183 | 210/210 | 207/212 | 121/141 | 191/217 | 133/147 | 119/131 | 152/154 | 210/216 |
| 632 | M | 645 | 204/204 | 229/237 | 136/136 | 191/203 | 137/145 | 192/204 | 152/154 | 146/178 | 243/243 | 122/159 | 170/172 | 181/189 | 210/216 | 207/212 | 127/127 | 189/205 | 147/155 | 114/119 | 152/154 | 175/208 |
| 633 | M | 646 | 204/204 | 229/237 | 136/136 | 191/203 | 137/145 | 186/192 | 152/152 | 146/178 | 243/243 | 122/161 | 170/172 | 181/189 | 210/216 | 207/212 | 127/127 | 189/205 | 147/155 | 114/119 | 152/154 | 175/210 |
| 634 | M | 647 | 204/240 | 229/251 | 130/134 | 191/195 | 125/135 | 184/204 | 154/154 | 140/178 | 243/263 | 122/144 | 174/183 | 183/183 | 210/220 | 207/212 | 118/124 | 191/217 | 147/153 | 114/119 | 152/160 | 175/208 |
| 635 | M | 648 | 204/214 | 237/247 | 161/178 | 199/203 | 134/135 | 160/184 | 154/168 | 140/160 | 243/267 | 161/173 | 172/172 | 183/183 | 210/220 | 207/223 | 118/121 | 189/199 | 155/176 | 119/131 | 152/152 | 172/175 |
| 636 | M | 649 | 204/212 | 229/241 | 130/130 | 191/203 | 129/137 | 170/170 | 156/156 | 146/146 | 243/243 | 148/148 | 168/172 | 183/193 | 210/216 | 212/225 | 121/141 | 191/205 | 147/147 | 116/131 | 152/164 | 175/212 |
| 637 | M | 650 | 204/225 | 241/255 | 130/130 | 203/203 | 129/137 | 160/194 | 156/156 | 140/152 | 254/267 | 148/181 | 168/174 | 189/193 | 226/226 | 207/212 | 124/127 | 189/191 | 147/157 | 125/131 | 152/164 | 172/185 |
| 638 | M | 651 | 212/214 | 241/241 | 130/186 | 191/209 | 132/137 | 174/204 | 138/138 | 140/178 | 263/263 | 122/173 | 174/176 | 183/185 | 206/206 | 207/212 | 124/127 | 191/197 | 147/157 | 125/131 | 152/152 | 175/175 |
| 639 | M | 652 | 204/212 | 229/251 | 130/130 | 191/197 | 125/135 | 170/204 | 154/170 | 178/178 | 243/263 | 122/144 | 170/174 | 183/183 | 210/210 | 207/212 | 121/127 | 191/217 | 133/157 | 119/131 | 152/157 | 166/210 |
| 640 | M | 653 | 204/214 | 229/251 | 130/138 | 195/203 | 123/135 | 182/192 | 154/164 | 140/160 | 243/254 | 154/173 | 176/180 | 185/185 | 220/220 | 210/212 | 118/124 | 191/217 | 133/180 | 127/131 | 152/154 | 177/212 |
| 641 | M | 654 | 204/204 | 229/247 | 159/161 | 201/203 | 134/134 | 196/202 | 156/218 | 150/156 | 243/263 | 122/122 | 166/172 | 183/183 | 216/239 | 212/212 | 121/147 | 197/197 | 137/171 | 114/119 | 152/160 | 208/208 |
| 642 | M | 655 | 204/212 | 243/251 | 129/161 | 203/203 | 129/135 | 160/170 | 154/154 | 146/178 | 243/243 | 124/148 | 168/180 | 181/183 | 206/210 | 212/212 | 127/141 | 189/199 | 147/169 | 119/125 | 152/168 | 172/216 |
| 643 | M | 656 | 212/225 | 229/241 | 130/186 | 191/203 | 129/137 | 170/194 | 156/156 | 146/152 | 243/267 | 122/148 | 172/174 | 185/193 | 216/216 | 212/225 | 121/127 | 191/205 | 147/147 | 125/131 | 152/164 | 175/185 |
| 644 | M | 657 | 204/204 | 247/249 | 130/163 | 203/203 | 137/141 | 192/202 | 158/216 | 146/160 | 243/243 | 124/152 | 170/172 | 181/183 | 210/239 | 212/212 | 118/124 | 199/205 | 137/137 | 114/127 | 152/160 | 172/175 |
| 645 | M | 658 | 212/268 | 234/251 | 130/130 | 195/203 | 134/145 | 160/204 | 154/176 | 140/178 | 243/263 | 144/148 | 172/174 | 183/189 | 206/210 | 212/221 | 121/121 | 189/197 | 157/171 | 125/134 | 152/154 | 210/214 |
| 646 | M | 659 | 204/204 | 237/251 | 130/130 | 191/203 | 135/137 | 192/208 | 154/154 | 146/178 | 243/243 | 122/173 | 172/174 | 183/189 | 210/210 | 207/212 | 118/127 | 205/217 | 133/147 | 116/119 | 152/168 | 210/214 |
| 647 | M | 660 | 204/204 | 229/243 | 152/152 | 203/205 | 125/125 | 160/186 | 156/214 | 146/146 | 243/243 | 122/177 | 170/178 | 181/198 | 210/210 | 212/212 | 124/141 | 195/205 | 153/153 | 119/127 | 152/160 | 172/175 |
| 648 | M | 661 | 204/214 | 237/241 | 130/138 | 191/197 | 134/147 | 180/204 | 160/160 | 146/150 | 243/263 | 122/148 | 168/174 | 183/185 | 210/220 | 207/212 | 121/127 | 183/197 | 147/157 | 107/116 | 154/168 | 198/214 |
| 649 | M | 662 | 204/204 | 243/251 | 138/138 | 199/203 | 129/135 | 160/170 | 156/156 | 140/178 | 254/263 | 122/124 | 172/174 | 183/183 | 210/210 | 207/210 | 127/141 | 191/217 | 133/171 | 127/134 | 154/157 | 175/210 |
| 650 | M | 663 | 204/212 | 237/243 | 129/130 | 191/203 | 129/134 | 170/184 | 162/162 | 140/178 | 254/263 | 148/152 | 168/180 | 181/185 | 206/206 | 210/212 | 121/127 | 189/197 | 171/171 | 114/116 | 160/168 | 187/214 |
| 651 | M | 664 | 204/206 | 234/243 | 129/152 | 203/205 | 129/135 | 192/196 | 152/152 | 178/178 | 243/243 | 122/124 | 172/189 | 181/187 | 206/216 | 212/212 | 121/124 | 191/205 | 141/147 | 116/127 | 152/160 | 172/175 |
| 652 | M | 665 | 204/204 | 237/237 | 154/186 | 203/211 | 123/137 | 202/204 | 170/170 | 140/178 | 243/263 | 144/148 | 166/168 | 183/185 | 220/220 | 207/212 | 121/141 | 183/205 | 147/157 | 116/119 | 154/168 | 214/214 |
| 653 | M | 666 | 204/212 | 229/241 | 130/159 | 203/203 | 129/134 | 202/204 | 152/152 | 146/178 | 243/243 | 148/152 | 166/174 | 181/185 | 206/206 | 212/212 | 124/127 | 189/197 | 171/171 | 125/127 | 152/160 | 175/175 |
| 654 | M | 667 | 204/212 | 229/241 | 130/163 | 203/203 | 129/134 | 202/204 | 152/152 | 146/178 | 243/243 | 148/152 | 166/174 | 181/185 | 206/206 | 212/212 | 124/127 | 189/197 | 171/171 | 125/127 | 152/160 | 175/175 |
| 655 | M | 668 | 204/216 | 229/237 | 130/145 | 199/203 | 125/135 | 188/204 | 156/208 | 140/160 | 254/254 | 173/181 | 168/176 | 183/183 | 220/220 | 212/230 | 124/127 | 189/217 | 133/155 | 131/134 | 154/164 | 212/212 |
| 656 | M | 669 | 204/221 | 234/237 | 138/186 | 203/213 | 134/145 | 170/194 | 156/156 | 146/150 | 243/254 | 148/148 | 168/176 | 183/185 | 206/220 | 210/212 | 121/127 | 191/197 | 157/171 | 116/119 | 168/168 | 192/212 |
| 657 | M | 670 | 204/212 | 237/247 | 132/161 | 195/203 | 123/134 | 160/184 | 154/162 | 130/178 | 263/267 | 148/177 | 176/180 | 189/189 | 210/220 | 207/212 | 124/141 | 183/197 | 176/176 | 127/131 | 154/157 | 172/194 |
| 658 | M | 671 | 204/204 | 241/251 | 129/161 | 203/205 | 134/135 | 160/170 | 154/154 | 176/178 | 243/263 | 148/173 | 174/178 | 183/189 | 210/210 | 207/212 | 124/141 | 197/199 | 171/184 | 116/119 | 152/168 | 175/210 |
| 659 | M | 672 | 204/204 | 241/251 | 129/161 | 203/205 | 134/137 | 160/170 | 154/154 | 176/178 | 243/263 | 148/173 | 174/178 | 183/189 | 210/210 | 207/212 | 124/141 | 197/199 | 171/184 | 116/119 | 152/168 | 175/210 |
| 660 | M | 673 | 204/204 | 241/251 | 129/161 | 203/205 | 134/135 | 160/170 | 154/154 | 176/178 | 243/263 | 148/173 | 174/178 | 183/189 | 210/210 | 207/212 | 124/141 | 197/199 | 174/184 | 116/119 | 152/168 | 175/210 |
| 661 | M | 674 | 204/204 | 241/251 | 129/157 | 203/205 | 134/135 | 160/170 | 154/154 | 176/178 | 243/263 | 148/173 | 174/178 | 183/189 | 210/210 | 207/212 | 124/141 | 197/199 | 171/186 | 116/119 | 152/168 | 175/210 |
| 662 | M | 675 | 204/204 | 241/251 | 129/165 | 203/205 | 134/135 | 160/170 | 154/154 | 174/178 | 243/263 | 148/173 | 174/178 | 183/189 | 210/210 | 207/212 | 124/141 | 197/199 | 171/184 | 116/119 | 152/168 | 175/210 |
| 663 | M | 676 | 204/212 | 241/247 | 159/161 | 203/203 | 129/135 | 170/192 | 152/152 | 140/160 | 243/263 | 124/148 | 166/172 | 183/189 | 210/210 | 212/212 | 124/144 | 205/217 | 174/186 | 114/119 | 152/160 | 210/216 |
| 664 | M | 677 | 204/214 | 237/251 | 129/148 | 195/205 | 134/134 | 164/202 | 170/170 | 140/160 | 263/267 | 148/169 | 164/168 | 183/189 | 210/220 | 207/212 | 121/124 | 197/217 | 171/171 | 116/125 | 152/152 | 175/179 |
| 665 | M | 678 | 204/204 | 247/251 | 129/159 | 205/205 | 135/135 | 160/180 | 156/218 | 172/178 | 243/263 | 152/179 | 166/172 | 183/183 | 210/210 | 206/212 | 118/121 | 199/217 | 133/178 | 119/127 | 152/152 | 172/175 |
| 666 | M | 679 | 214/214 | 229/251 | 129/129 | 203/203 | 135/135 | 160/202 | 156/218 | 146/152 | 261/261 | 122/148 | 166/170 | 181/183 | 206/210 | 207/212 | 118/135 | 199/199 | 184/190 | 125/127 | 157/173 | 194/196 |
| 667 | M | 680 | 212/214 | 237/241 | 129/150 | 197/203 | 129/159 | 170/180 | 166/166 | 146/150 | 263/267 | 122/171 | 168/174 | 181/183 | 206/220 | 212/232 | 121/124 | 189/189 | 174/174 | 114/127 | 154/160 | 216/216 |
| 668 | M | 681 | 204/212 | 241/247 | 186/186 | 203/203 | 135/137 | 160/170 | 194/218 | 146/178 | 243/243 | 148/173 | 168/172 | 183/189 | 210/210 | 212/212 | 141/141 | 199/205 | 147/184 | 116/127 | 152/157 | 172/212 |
| 669 | M | 682 | 204/212 | 241/247 | 186/186 | 203/203 | 135/137 | 160/170 | 194/218 | 146/178 | 243/243 | 148/177 | 168/172 | 183/189 | 210/210 | 212/212 | 141/141 | 199/205 | 147/184 | 116/127 | 152/157 | 172/212 |
| 670 | M | 683 | 206/240 | 234/249 | 152/198 | 201/205 | 135/137 | 170/196 | 216/216 | 162/178 | 243/243 | 124/173 | 172/189 | 187/196 | 206/210 | 212/212 | 118/124 | 205/217 | 141/171 | 114/116 | 152/160 | 170/172 |
| 671 | M | 684 | 212/272 | 241/241 | 129/129 | 203/203 | 135/139 | 160/180 | 154/154 | 130/178 | 243/263 | 144/148 | 172/174 | 183/189 | 210/210 | 212/212 | 121/121 | 189/197 | 159/184 | 125/127 | 152/157 | 175/210 |
| 672 | M | 685 | 204/204 | 229/234 | 130/130 | 195/203 | 135/139 | 202/204 | 154/176 | 130/140 | 243/263 | 152/152 | 174/180 | 183/183 | 206/210 | 212/221 | 121/127 | 183/217 | 133/159 | 125/127 | 152/157 | 175/212 |

^1^ In bold represent sharing genotypes between both WOGBM and WOGBC

^2^ C: Cordoba, M: Marrakech.
