# Supplementary material for: Characterization of Worldwide Olive Germplasm Banks of Marrakech (Morocco) and Córdoba (Spain): Towards management and use of olive germplasm in breeding programs
Source: PLoS One. 2019 Oct 17;14(10):e0223716. doi: 10.1371/journal.pone.0223716 (PMC6797134; doi:10.1371/journal.pone.0223716)
Supplement: S4 Table — Shared cultivars and specific to each collection are indicated. (DOCX) [file pone.0223716.s004.docx]

**S4 Table.** List of identified and authenticated cultivars in both collections. Shared cultivars and specific to each collection are indicated.

| **No.** | **WOGBC** | **WOGBM** | **Cultivar Name^1^** | **Cultivation area^2^** | **Code Morpho.** | **Weigth^3^** | **Shape.A^4^** | **Symm.A^5^** | **Symm. B^6^** | **T Diam.B^7^** | **Apex.A^8^** | **Base. A^9^** | **Rugosity^10^** | **N. grooves^11^** | **Dist. grooves^12^** | **Mucro^13^** |
| --- | --- | --- | --- | --- | --- | --- | --- | --- | --- | --- | --- | --- | --- | --- | --- | --- |
| 1 |  | x | Aaleth | ALG | 260 | L | EL | A | S | C | P | P | R | H | R | P |
| 2 |  | x | Abbadi Abou Gabra-610 | SYR | 257 | H | EP | SA | SA | C | P | P | R | M | R | P |
| 3 | x |  | Abbadi Abou Gabra-842 | SYR | 240 | VH | EP | SA | SA | C | P | R | R | M | R | P |
| 4 |  | x | *Abbadi Helo* | SYR |  | No Data | | | | | | | | | | |
| 5 | x | x | Abbadi Shalal | SYR | 234 | VH | EL | SA | SA | C | P | P | R | M | R | P |
| 6 |  | x | Abiad Min Omou | SYR | 316 | VH | S | S | S | C | R | T | SC | L | G | A |
| 7 |  | x | Abou Anaked | SYR |  | No Data | | | | | | | | | | |
| 8 | x |  | Abou Choki-1115 | SYR | 235 | VH | EL | SA | SA | C | P | R | R | L | G | P |
| 9 | x |  | Abou Choki-1126 | SYR | 194 | M | EP | S | S | C | P | R | S | M | R | P |
| 10 | x |  | Abou Kanani | SYR | 234 | VH | EL | SA | SA | C | P | P | R | M | R | P |
| 11 |  | x | Abou Monkar | EGY | 308 | L | EP | A | SA | C | P | P | R | M | R | P |
| 12 |  | x | Abunara | ITA | 45 | H | EP | SA | SA | C | P | R | R | M | R | P |
| 13 | x |  | *Acebuche de Caravaca-800* | SP | 176 | M | EP | SA | SA | C | P | P | S | M | G | P |
| 14 | x | x | **Acebuchera** | SP | 51 | H | EP | SA | S | A | P | R | R | M | G | P |
| 15 | x | x | **Adkam^a^** | SYR | 50 | H | EP | SA | S | A | P | R | R | H | R | A |
| 16 |  | x | Aggezi Akse | EGY | 4 | H | EL | A | SA | C | P | P | R | M | R | P |
| 17 |  | x | Aggezi Oshime | EGY | 305 | H | EL | SA | SA | C | P | P | R | L | R | P |
| 18 |  | x | Aggezi Shami | EGY | 318 | H | EP | SA | SA | C | R | P | SC | H | R | P |
| 19 |  | x | Aglandau | FRA | 255 | M | EP | SA | S | C | R | P | R | M | R | P |
| 20 | x |  | Agouromanakolia | GRC | 221 | M | O | S | S | C | P | T | R | M | R | A |
| 21 |  | x | Agrarez | ALG | 93 | H | O | SA | S | C | P | R | R | M | R | P |
| 22 |  | x | Aguenaou | ALG | 236 | VH | EL | SA | S | C | P | P | SC | M | G | P |
| 23 |  | x | Aharoun | ALG | 4 | H | EL | A | SA | C | P | P | R | M | R | P |
| 24 |  | x | Ahia Ousbaa | ALG | 300 | M | EL | SA | S | A | P | P | R | L | R | P |
| 25 |  | x | Aîmel | ALG | 368 | M | EP | SA | S | A | R | R | S | L | R | P |
| 26 |  | x | Aitana | ITA | 340 | H | EP | A | S | C | R | P | R | L | R | P |
| 27 |  | x | Akenane | ALG | 303 | M | EL | SA | S | C | P | P | R | M | R | P |
| 28 |  | x | Akerma | ALG | 269 | VH | EP | SA | S | C | P | R | R | H | R | P |
| 29 | x | x | **Alameño Blanco** | SP | 81 | H | O | A | SA | C | P | R | R | M | G | P |
| 30 | x |  | **Alameño de Cabra** | SP | 61 | H | EP | SA | S | C | P | R | R | M | R | P |
| 31 | x | x | **Alameño de Montilla** | SP | 66 | H | EP | SA | S | C | P | T | R | M | R | P |
| 32 |  | x | Albatro | ITA | 333 | H | EP | SA | S | C | R | T | SC | M | G | P |
| 33 | x |  | **Alfafara** | SP | 60 | H | EP | SA | S | C | P | R | R | M | G | P |
| 34 |  | x | Allora | ITA | 188 | M | EP | SA | S | C | R | R | R | M | R | P |
| 35 | x |  | **Aloreña de Iznalloz** | SP | 99 | H | O | SA | S | C | R | R | R | M | R | P |
| 36 | x |  | *Aloreña de Iznalloz-778* | SP |  | No Data | | | | | | | | | | |
| 37 | x |  | *Aloreña-829* | ARG |  | No Data | | | | | | | | | | |
| 38 | x | x | **Amargoso** | SP | 167 | M | EP | A | S | C | P | T | R | M | R | P |
| 39 |  | x | Americano | ITA | 286 | M | EP | A | S | A | R | P | S | L | R | P |
| 40 | x |  | **Amygdalolia Nana** | GRC | 229 | VH | EL | A | S | A | P | P | R | M | R | P |
| 41 | x | x | **Arbequina** | SP | 132 | L | O | S | S | C | R | R | R | M | R | A |
| 42 | x |  | **Arbosana** | SP | 128 | L | O | SA | S | C | R | R | S | M | R | P |
| 43 | x |  | **Argudell** | SP | 118 | L | EP | A | SA | B | P | P | R | M | G | P |
| 44 | x |  | *Arroniz-1172* | SP |  | No Data | | | | | | | | | | |
| 45 | x |  | **Arroniz** | SP | 74 | H | EP | S | S | A | P | P | R | L | G | P |
| 46 | x | x | **Ascolana Tenera** | ITA | 39 | H | EP | A | S | C | P | T | SC | H | R | P |
| 47 | x |  | **Asnal** | SP | 58 | H | EP | SA | S | C | P | P | R | M | R | A |
| 48 |  | x | Atounsi Setif | ALG | 269 | VH | EP | SA | S | C | P | P | SC | H | R | P |
| 49 | x |  | **Ayvalik** | TUR | 106 | H | O | S | S | C | R | R | R | M | G | P |
| 50 | x |  | **Azapa** | CHL | 15 | H | EL | A | S | C | P | T | R | M | R | P |
| 51 |  | x | Azeboudj de Khirane | ALG |  | No Data | | | | | | | | | | |
| 52 |  | x | Azeradj Tamokra | ALG | 32 | H | EP | A | S | C | P | P | R | H | R | P |
| 53 |  | x | Azeradj | ALG | 32 | H | EP | A | S | C | P | P | R | H | R | P |
| 54 | x | x | **Azul** | SP | 33 | H | EP | A | S | C | P | P | R | M | R | P |
| 55 | x |  | **Azulejo** | SP | 38 | H | EP | A | S | C | P | T | R | M | R | P |
| 56 |  | x | Baid El Hamam | EGY | 276 | VH | O | SA | S | C | R | R | R | H | R | P |
| 57 |  | x | Baladi | EGY | 307 | VH | EP | SA | S | C | R | P | R | M | R | P |
| 58 | x |  | **Barnea** | ISR | 151 | M | EL | SA | S | C | P | P | R | M | G | A |
| 59 |  | x | Barouni | TUN | 99 | H | O | SA | S | C | R | R | R | M | R | P |
| 60 | x |  | Barri | SYR | 211 | M | O | SA | S | C | P | R | R | M | R | A |
| 61 |  | x | Bed Al Iguel | SYR | 374 | H | O | A | S | B | P | R | R | M | R | P |
| 62 | x | x | **Beladi** | LBN | 153 | M | EL | SA | S | C | P | P | S | M | G | P |
| 63 |  | x | Beladi-577 | LBN | 262 | VH | EL | A | S | C | P | P | R | M | R | P |
| 64 |  | x | Beldi | TUN | 250 | H | O | SA | S | C | R | P | S | M | R | P |
| 65 | x |  | **Belluti** | TUR | 230 | VH | EL | A | S | A | P | T | R | M | G | A |
| 66 | x |  | Bent al Kadi | SYR | 237 | VH | EL | SA | S | C | P | T | R | M | R | P |
| 67 |  | x | Berri Meslal-397 | MOR | 324 | M | EL | A | S | C | P | P | S | M | R | P |
| 68 |  | x | Berri Meslal-532 | MOR | 282 | H | EP | A | S | C | R | R | R | M | R | P |
| 69 |  | x | Besbessi | TUN | 304 | VH | EL | SA | S | A | R | P | R | L | R | A |
| 70 | x |  | Beyaz Yaglik | TUR | 220 | M | O | S | S | A | R | P | R | M | R | A |
| 71 |  | x | Bez El Anza | EGY | 373 | M | EL | A | S | C | P | P | S | L | R | P |
| 72 | x |  | Biancolilla-1814 | ITA | 61 | H | EP | SA | S | C | P | R | R | M | R | P |
| 73 |  | x | Biancolilla-83 | ITA | 67 | H | EP | SA | S | C | R | P | R | M | R | P |
| 74 | x | x | **Bical** | SP | 8 | H | EL | A | S | A | P | P | R | M | G | P |
| 75 |  | x | *Bissani-578* | LBN |  | No Data | | | | | | | | | | |
| 76 | x | x | **Blanqueta** | SP | 130 | L | O | S | S | A | R | P | S | M | G | P |
| 77 | x |  | **Bodoquera** | SP | 101 | H | O | SA | S | C | R | R | SC | M | R | A |
| 78 | x | x | **Bolvino** | SP | 54 | H | EP | SA | S | A | R | P | S | M | R | P |
| 79 | x | x | **Borriolenca** | SP | 61 | H | EP | SA | S | C | P | R | R | M | R | P |
| 80 | x | x | **Bosana** | ITA | 170 | M | EP | SA | SA | A | R | P | R | M | R | P |
| 81 |  | x | Bottone di gallo | ITA | 146 | M | EL | A | S | C | P | P | R | M | R | P |
| 82 |  | x | Bouchouika | MOR | 9 | H | EL | A | S | A | P | P | R | M | R | P |
| 83 |  | x | Bouchouk Lafayette | ALG | 299 | H | EP | SA | S | C | P | P | SC | H | R | P |
| 84 | x | x | **Bouteillan** | FRA | 40 | H | EP | A | S | C | P | T | SC | M | R | P |
| 85 |  | x | Brandofino | ITA | 234 | VH | EL | SA | SA | C | P | P | R | M | R | P |
| 86 | x | x | **Buga^a^** | HRV | 209 | M | O | SA | S | C | P | P | R | M | R | P |
| 87 | x |  | **Buidiego** | SP | 13 | H | EL | A | S | C | P | R | R | M | R | A |
| 88 | x |  | **Caballo** | SP | 69 | H | EP | SA | S | C | R | R | R | M | G | P |
| 89 |  | x | Cairo 7 | EGY | 259 | M | EL | A | S | C | P | P | R | L | R | P |
| 90 |  | x | Calatina | ITA | 341 | H | EL | A | SA | C | P | R | R | M | R | P |
| 91 | x | x | **Callosina** | SP | 140 | M | EL | A | SA | C | P | P | S | M | R | P |
| 92 | x |  | **Canetera** | SP | 149 | M | EL | SA | S | A | P | P | S | M | G | P |
| 93 | x |  | **Caninese** | ITA | 119 | L | EP | A | SA | C | R | P | R | M | R | P |
| 94 | x | x | **Cañivano Negro** | SP | 7 | H | EL | A | SA | C | R | R | R | M | R | P |
| 95 | x |  | *Cañivano Negro-55* | SP | 37 | H | EP | A | S | C | P | T | R | M | G | P |
| 96 |  | x | Cariasina | ITA | 342 | H | O | SA | S | B | P | T | SC | L | G | A |
| 97 | x | x | **Carolea** | ITA | 53 | H | EP | SA | S | A | R | P | R | M | G | P |
| 98 | x |  | **Carrasqueño de Alcaudete** | SP | 168 | M | EP | A | S | C | R | P | R | M | R | P |
| 99 | x | x | **Carrasqueño de Elvas** | PRT | 70 | H | EP | SA | S | C | R | R | R | M | R | P |
| 100 | x | x | **Carrasqueño de Jumilla** | SP | 59 | H | EP | SA | S | C | P | P | R | M | R | P |
| 101 | x |  | **Carrasqueño de la Sierra** | SP | 177 | M | EP | SA | SA | C | P | P | S | M | R | P |
| 102 | x |  | **Carrasqueño de Porcuna** | SP | 59 | H | EP | SA | S | C | P | P | R | M | R | P |
| 103 | x | x | **Carrasquillo** | SP | 170 | M | EP | SA | SA | A | R | P | R | M | R | P |
| 104 |  | x | Cassanese | ITA | 330 | H | EP | SA | S | A | R | R | SC | H | R | A |
| 105 | x |  | **Castellana** | SP | 185 | M | EP | SA | S | C | P | P | R | M | R | P |
| 106 |  | x | Castricianella rapparina | ITA | 345 | M | EP | SA | S | C | P | R | R | L | G | P |
| 107 |  | x | Cavalieri | ITA | 346 | M | EP | SA | S | C | R | R | S | L | G | P |
| 108 |  | x | Cayon | FRA | 185 | M | EP | SA | S | C | P | P | R | M | R | P |
| 109 |  | x | Cerasuola | ITA | 344 | M | O | SA | S | A | R | P | R | M | R | A |
| 110 | x | x | **Cerezuela** | SP | 210 | M | O | SA | S | C | P | R | R | M | G | P |
| 111 | x |  | Chalchali | SYR | 103 | H | O | SA | S | C | R | T | R | M | G | P |
| 112 | x | x | **Chalkidikis^a^** | GRC | 21 | H | EL | SA | S | A | P | P | R | M | R | P |
| 113 | x | x | **Changlot Real** | SP | 169 | M | EP | SA | SA | A | R | P | R | M | G | P |
| 114 |  | x | Chemchali | TUN | 322 | H | O | SA | S | C | R | R | R | L | G | A |
| 115 | x | x | **Chemlal de Kabilye^a^** | ALG | 156 | M | EP | A | SA | C | P | R | R | M | R | A |
| 116 | x |  | Chemlali-744 | TUN | 161 | M | EP | A | S | B | P | R | R | M | R | P |
| 117 | x | x | **Chetoui** | TUN | 147 | M | EL | A | S | C | P | R | R | M | R | P |
| 118 | x |  | **Chorreao de Montefrío** | SP | 30 | H | EP | A | S | A | P | P | R | M | R | P |
| 119 | x |  | *Chorruo de Castro del Río-361* | SP | 93 | H | O | SA | S | C | P | R | R | M | R | P |
| 120 | x | x | **Chorruo** | SP | 205 | M | O | SA | S | A | R | P | S | M | R | P |
| 121 |  | x | Ciciarello | ITA | 326 | M | O | SA | SA | A | P | R | S | M | R | P |
| 122 |  | x | Cima di Melfi | ITA | 343 | M | EP | SA | SA | C | R | R | R | M | G | P |
| 123 | x |  | **Cipresino** | ITA | 65 | H | EP | SA | S | C | P | T | R | M | G | P |
| 124 | x | x | **Cirujal** | SP | 17 | H | EL | SA | SA | A | P | P | S | M | G | P |
| 125 | x | x | **Çobrancosa** | PRT | 3 | H | EL | A | SA | C | P | P | R | M | G | P |
| 126 |  | x | Confetto | ITA | 249 | VH | EP | SA | S | A | R | P | R | M | R | P |
| 127 | x | x | **Coratina** | ITA | 49 | H | EP | SA | S | A | P | P | R | M | R | P |
| 128 | x | x | **Corbella** | SP | 134 | M | EL | A | SA | A | P | P | R | M | R | P |
| 129 | x |  | *Corbella-817* | SP | 145 | M | EL | A | S | A | P | T | R | M | R | P |
| 130 | x |  | Cordobés de Arroyo de la Luz | SP | 212 | M | O | SA | S | C | P | T | R | M | R | P |
| 131 | x |  | Cordovil de Castelo Branço | PRT | 58 | H | EP | SA | S | C | P | P | R | M | R | A |
| 132 | x | x | **Cordovil de Serpa** | PRT | 52 | H | EP | SA | S | A | R | P | R | L | G | P |
| 133 | x | x | **Cornezuelo de Jaen** | SP | 6 | H | EL | A | SA | C | P | P | S | M | R | P |
| 134 | x |  | **Cornicabra de Jerez Caballeros** | SP | 34 | H | EP | A | S | C | P | R | R | M | R | P |
| 135 | x |  | **Cornicabra de Mérida** | SP | 139 | M | EL | A | SA | C | P | P | S | M | G | P |
| 136 | x | x | **Cornicabra** | SP | 137 | M | EL | A | SA | C | P | P | R | M | R | A |
| 137 | x |  | *Corralones de Andujar-790* | SP | 48 | H | EP | SA | S | A | P | P | R | H | R | A |
| 138 |  | x | Craputea | ITA | 261 | M | EP | SA | S | C | P | P | R | H | R | P |
| 139 |  | x | Crastu | ITA | 188 | M | EP | SA | S | C | R | R | R | M | R | P |
| 140 | x |  | Crnica | HRV | 100 | H | O | SA | S | C | R | R | S | M | G | A |
| 141 |  | x | *Crnica-399* | SLV | 98 | H | O | SA | S | C | R | R | R | M | G | P |
| 142 |  | x | Cucca | ITA | 252 | H | O | SA | S | C | R | R | SC | L | R | P |
| 143 |  | x | Cuoricino | ITA | 334 | L | O | S | S | C | R | R | R | M | G | P |
| 144 | x |  | **Curivell** | SP | 190 | M | EP | S | S | A | R | P | R | M | G | P |
| 145 | x |  | **Datilero** | SP | 4 | H | EL | A | SA | C | P | P | R | M | R | P |
| 146 | x |  | De Sal | SP | 203 | M | O | SA | SA | C | P | T | R | M | R | P |
| 147 | x |  | Desconocida 1481 | Unknown |  | No Data | | | | | | | | | | |
| 148 |  | x | Dhokar | TUN | 255 | M | EP | SA | S | C | R | P | R | M | R | P |
| 149 |  | x | Djlot Tadmori | SYR | 206 | M | O | SA | S | A | R | R | R | H | R | P |
| 150 | x |  | Doebli | SYR | 90 | H | O | SA | S | C | P | R | R | L | R | A |
| 151 | x |  | Dokkar | TUR | 115 | L | EL | S | SA | C | P | P | S | M | R | P |
| 152 | x |  | **Dolce Agogia** | ITA | 87 | H | O | SA | S | A | R | R | SC | M | R | P |
| 153 |  | x | Dolce di Rossano | ITA | 328 | M | O | S | S | C | R | R | S | M | R | A |
| 154 | x |  | Dolce | EGY | 124 | L | EP | SA | S | A | R | P | R | M | R | P |
| 155 | x |  | **Domat** | TUR | 25 | H | EL | SA | S | C | R | P | R | L | G | A |
| 156 |  | x | Dressi | TUN | 288 | L | EL | A | S | C | P | P | S | M | R | P |
| 157 |  | x | Dritta di Moscufa | ITA | 376 | M | EP | S | S | A | P | R | R | L | R | P |
| 158 | x |  | Dulzal de Carmona | SP | 42 | H | EP | SA | SA | A | P | R | R | M | R | A |
| 159 | x |  | *Dulzal de Carmona-31* | SP |  | No Data | | | | | | | | | | |
| 160 | x | x | **Dulzal** | SP | 49 | H | EP | SA | S | A | P | P | R | M | R | P |
| 161 | x |  | Dwarf D | USA | 76 | H | EP | S | S | C | P | P | R | M | R | P |
| 162 |  | x | El Lewa | EGY | 258 | M | EP | A | S | C | P | P | S | L | R | P |
| 163 |  | x | El Salam | EGY | 265 | M | EL | A | SA | C | P | P | S | L | R | P |
| 164 | x |  | Elmacik | TUR | 246 | VH | O | SA | S | C | R | T | R | M | R | P |
| 165 |  | x | Emilia | ITA | 274 | M | O | SA | S | C | R | R | R | M | R | P |
| 166 | x | x | **Empeltre** | SP | 142 | M | EL | A | S | A | P | P | R | H | R | P |
| 167 | x | x | **Enagua de Arenas** | SP | 67 | H | EP | SA | S | C | R | P | R | M | R | P |
| 168 | x | x | Ensasi | SYR | 210 | M | O | SA | S | C | P | R | R | M | G | P |
| 169 | x |  | Erbek Yaglik | TUR | 182 | M | EP | SA | S | A | R | P | R | M | R | P |
| 170 | x |  | **Escarabajillo** | SP | 208 | M | O | SA | S | B | R | R | R | H | R | P |
| 171 | x |  | **Escarabajuelo de Atarfe** | SP | 59 | H | EP | SA | S | C | P | P | R | M | R | P |
| 172 | x | x | **Escarabajuelo de Posadas** | SP | 41 | H | EP | A | S | C | R | R | R | M | G | P |
| 173 | x | x | **Escarabajuelo de Úbeda** | SP | 232 | VH | EL | A | S | C | P | T | R | H | R | P |
| 174 | x | x | **Farga** | SP | 136 | M | EL | A | SA | A | P | P | S | M | R | P |
| 175 |  | x | Fasolona | ITA | 347 | H | EP | SA | S | C | R | R | R | L | R | P |
| 176 |  | x | Ferkani | ALG | 291 | M | EP | A | S | A | P | P | S | M | R | P |
| 177 | x |  | Figueretes | SP | 125 | L | EP | SA | S | A | R | P | S | M | R | P |
| 178 | x |  | **Fishomi** | IRA | 56 | H | EP | SA | S | B | P | T | S | M | R | P |
| 179 | x |  | Forastera de Tortosa | SP | 133 | M | EL | A | SA | A | P | P | R | M | R | A |
| 180 |  | x | Fouji vert | TUN | 264 | H | EL | SA | SA | A | P | P | R | M | R | A |
| 181 | x | x | **Frantoio** | ITA | 172 | M | EP | SA | SA | A | R | R | R | H | R | P |
| 182 | x | x | **Fulla de Salze** | SP | 112 | L | EL | A | S | C | P | P | S | M | G | P |
| 183 | x | x | **Galega Vulgar** | PRT | 175 | M | EP | SA | SA | C | P | P | R | M | R | A |
| 184 | x |  | **Gatuno** | SP | 34 | H | EP | A | S | C | P | R | R | M | R | P |
| 185 | x |  | Gaydoyrelia | GRC | 233 | VH | EL | SA | SA | A | P | P | R | M | R | P |
| 186 | x | x | **Gemlik** | TUR | 206 | M | O | SA | S | A | R | R | R | H | R | P |
| 187 | x |  | **Genovesa** | SP | 75 | H | EP | S | S | C | P | P | R | M | G | P |
| 188 |  | x | Gentile di chieti | ITA | 84 | H | O | SA | S | A | R | P | R | M | R | P |
| 189 | x | x | **Gerboui** | TUN | 94 | H | O | SA | S | C | P | T | R | M | G | A |
| 190 |  | x | Gerboui-298 | TUN | 70 | H | EP | SA | S | C | R | R | R | M | R | P |
| 191 |  | x | Giarfara | ITA | 278 | H | O | SA | S | C | R | R | R | M | R | P |
| 192 |  | x | Giarraffa | ITA | 271 | VH | EL | SA | S | C | P | P | SC | H | R | P |
| 193 | x |  | Gjykatesi | ALB |  | No Data | | | | | | | | | | |
| 194 | x | x | **Gordal de Granada** | SP | 61 | H | EP | SA | S | C | P | R | R | M | R | P |
| 195 | x |  | **Gordal de Hellín** | SP | 70 | H | EP | SA | S | C | R | R | R | M | R | P |
| 196 | x |  | Gordal de Vélez Rubio | SP | 214 | M | O | SA | S | C | R | P | S | M | R | P |
| 197 | x | x | **Gordal Sevillana** | SP | 236 | VH | EL | SA | S | C | P | P | SC | M | G | P |
| 198 | x | x | **Grappolo** | ITA | 171 | M | EP | SA | SA | A | R | P | S | M | R | P |
| 199 |  | x | Gremigno di Fauglia | ITA | 280 | L | EP | SA | S | C | R | R | S | L | R | P |
| 200 | x |  | **Grit Eytini** | TUR | 127 | L | EP | SA | S | C | P | P | S | M | R | P |
| 201 | x |  | **Grosal de Cieza** | SP | 200 | M | O | A | S | C | R | R | R | M | G | P |
| 202 | x |  | Grosal Vimbodí | SP | 227 | M | S | SA | S | C | R | R | R | M | R | A |
| 203 |  | x | Grossa di Spagna | ITA | 266 | VH | EL | A | SA | C | P | P | SC | M | R | P |
| 204 |  | x | *Grossane-194* | FRA | 69 | H | EP | SA | S | C | R | R | R | M | G | P |
| 205 | x |  | *Grossanne-67* | FRA | 4 | H | EL | A | SA | C | P | P | R | M | R | P |
| 206 | x |  | Habichuelero de Baena | SP | 184 | M | EP | SA | S | C | P | P | R | M | G | A |
| 207 | x | x | **Habichuelero de Grazalema** | SP | 135 | M | EL | A | SA | A | P | P | S | M | R | A |
| 208 |  | x | Hamed | EGY | 248 | H | EP | SA | S | A | R | P | SC | M | R | P |
| 209 |  | x | Hamra | ALG | 256 | L | EP | SA | S | C | R | P | R | M | R | P |
| 210 | x |  | Hemblasi | SYR |  | No Data | | | | | | | | | | |
| 211 |  | x | *Hemblasi-601* | SYR | 320 | H | EL | SA | SA | C | P | R | SC | H | R | P |
| 212 |  | x | Heraktane | SYR | 20 | H | EL | SA | SA | C | P | R | R | H | R | P |
| 213 | x | x | **Hojiblanca** | SP | 70 | H | EP | SA | S | C | R | R | R | M | R | P |
| 214 |  | x | Humaisi | SYR | 315 | L | EP | A | S | C | P | P | R | M | R | P |
| 215 |  | x | Idleb | SYR | 277 | VH | O | S | S | C | R | R | SC | H | R | P |
| 216 |  | x | Ifiri | ALG | 4 | H | EL | A | SA | C | P | P | R | M | R | P |
| 217 | x |  | Imperial de Jaén | SP | 42 | H | EP | SA | SA | A | P | R | R | M | R | A |
| 218 | x |  | **Imperial** | SP | 205 | M | O | SA | S | A | R | P | S | M | R | P |
| 219 |  | x | Intosso | ITA | 327 | H | EL | SA | S | A | R | P | R | H | R | P |
| 220 | x |  | Istarska Bjelica | HRV | 178 | M | EP | SA | SA | C | P | R | R | M | R | P |
| 221 |  | x | Istarska crnica | HRV | 275 | M | O | S | S | C | R | R | R | H | R | P |
| 222 | x | x | **Itrana** | ITA | 55 | H | EP | SA | S | A | R | R | SC | M | R | P |
| 223 | x |  | **Izmir Sofralik** | TUR | 68 | H | EP | SA | S | C | R | R | R | H | R | P |
| 224 | x |  | Jabali | SYR | 223 | M | O | S | S | C | R | P | S | L | G | A |
| 225 | x | x | **Jabaluna** | SP | 34 | H | EP | A | S | C | P | R | R | M | R | P |
| 226 | x | x | **Jaropo** | SP | 154 | M | EP | A | SA | C | P | P | R | M | R | P |
| 227 | x | x | Jlot | SYR |  | No Data | | | | | | | | | | |
| 228 | x |  | Jlot-841 | SYR | 239 | VH | EP | SA | SA | C | P | R | R | H | R | P |
| 229 | x |  | **Joanenca** | SP | 191 | M | EP | S | S | A | R | P | S | L | G | P |
| 230 |  | x | Kaissy | SYR | 252 | H | O | SA | S | C | R | R | SC | M | R | P |
| 231 | x |  | **Kalamon** | GRC | 11 | H | EL | A | S | C | P | P | R | M | G | A |
| 232 | x |  | **Kalinjot** | ALB | 97 | H | O | SA | S | C | R | R | R | L | G | P |
| 233 | x |  | Kallmet | ALB | 102 | H | O | SA | S | C | R | T | R | L | R | P |
| 234 | x | x | **Kalokerida^a^** | GRC | 174 | M | EP | SA | SA | B | P | R | R | L | G | P |
| 235 | x |  | Kan Çelebi | TUR | 239 | VH | EP | SA | SA | C | P | R | R | H | R | P |
| 236 | x | x | Karamani | SYR | 24 | H | EL | SA | S | C | P | R | R | M | R | P |
| 237 |  | x | Karbuncela | HRV | 70 | H | EP | SA | S | C | R | R | R | M | R | P |
| 238 |  | x | Karme | SYR | 60 | H | EP | SA | S | C | P | R | R | M | G | P |
| 239 |  | x | Karolia | GRC | 23 | H | EL | SA | S | C | P | P | R | M | R | P |
| 240 |  | x | Karydolia | GRC | 356 | VH | EL | A | SA | C | P | R | R | M | R | P |
| 241 | x | x | **Kato Drys** | CYP | 61 | H | EP | SA | S | C | P | R | R | M | R | P |
| 242 | x |  | Kelb et Ter | SYR | 44 | H | EP | SA | SA | C | P | P | R | M | G | P |
| 243 |  | x | Kerdi | SYR |  | No Data | | | | | | | | | | |
| 244 | x |  | Kerkiras | GRC | 120 | L | EP | A | S | C | P | P | S | M | R | P |
| 245 |  | x | Khadraya | ALG | 301 | M | EP | SA | S | C | R | R | S | L | R | P |
| 246 | x |  | Khalkhali | SYR |  | No Data | | | | | | | | | | |
| 247 |  | x | *Khalkhali-629* | SYR | 10 | H | EL | A | S | C | P | P | R | H | R | P |
| 248 | x |  | Khashabi | SYR | 23 | H | EL | SA | S | C | P | P | R | M | R | P |
| 249 |  | x | *Khashabi-631* | SYR |  | No Data | | | | | | | | | | |
| 250 |  | x | Khnfse | SYR | 317 | VH | O | SA | S | B | P | T | SC | H | R | A |
| 251 |  | x | Khodieri | SYR | 187 | M | EP | SA | S | C | P | R | R | M | R | P |
| 252 | x |  | Kiraz | TUR | 94 | H | O | SA | S | C | P | T | R | M | G | A |
| 253 | x |  | *Klon-14-1081-1* | ALB | 218 | M | O | S | SA | A | R | P | S | L | G | P |
| 254 | x |  | *Klon-14-1081-2* | ALB |  | No Data | | | | | | | | | | |
| 255 | x | x | **Kolybada^a^** | GRC | 110 | H | S | SA | S | C | R | R | R | M | R | P |
| 256 | x |  | **Konservolia** | GRC | 64 | H | EP | SA | S | C | P | R | SC | M | G | P |
| 257 | x | x | **Koroneiki** | GRC | 114 | L | EL | SA | S | C | P | P | S | M | R | P |
| 258 |  | x | Kossiem | EGY | 283 | M | EL | A | S | C | R | P | R | M | R | P |
| 259 |  | x | Kothreiki | GRC | 253 | M | O | SA | S | C | R | R | R | L | R | P |
| 260 | x |  | **Kotruvsi** | ALB | 99 | H | O | SA | S | C | R | R | R | M | R | P |
| 261 |  | x | Koutsourelia | GRC | 357 | L | EP | A | S | C | P | P | S | L | G | P |
| 262 | x | x | **Lastovka** | HRV | 22 | H | EL | SA | S | A | P | P | S | M | R | A |
| 263 |  | x | Lastrino | ITA | 331 | L | EL | A | S | C | R | P | S | M | R | P |
| 264 |  | x | Lazzero di prata | ITA | 365 | L | EP | A | S | C | R | P | S | L | R | P |
| 265 |  | x | Lazzero | ITA | 332 | H | EL | A | SA | C | R | P | SC | M | R | P |
| 266 | x | x | **Leccino** | ITA | 158 | M | EP | A | SA | C | R | R | R | H | R | P |
| 267 |  | x | Leccio Maremmano | ITA | 189 | M | EP | SA | S | C | R | R | S | M | R | P |
| 268 | x | x | **Lechin de Granada** | SP | 171 | M | EP | SA | SA | A | R | P | S | M | R | P |
| 269 | x | x | **Lechín de Sevilla** | SP | 177 | M | EP | SA | SA | C | P | P | S | M | R | P |
| 270 | x |  | **Lemeño** | SP | 168 | M | EP | A | S | C | R | P | R | M | R | P |
| 271 | x |  | **Lentisca** | SP | 121 | L | EP | A | S | C | R | R | R | M | R | P |
| 272 |  | x | *Lentisca-206* | PRT | 289 | L | EP | A | S | C | R | R | S | M | R | P |
| 273 |  | x | Lentisca-244 | SP | 146 | M | EL | A | S | C | P | P | R | M | R | P |
| 274 | x |  | **Levantinka** | HRV | 165 | M | EP | A | S | C | P | R | R | M | R | A |
| 275 | x | x | **Limoncillo** | SP | 9 | H | EL | A | S | A | P | P | R | M | R | P |
| 276 | x | x | **Llorón de Atarfe** | SP | 99 | H | O | SA | S | C | R | R | R | M | R | P |
| 277 | x | x | **Llumeta** | SP | 150 | M | EL | SA | S | A | R | R | S | M | R | A |
| 278 | x | x | **Loaime** | SP | 204 | M | O | SA | S | A | R | P | R | M | G | P |
| 279 | x |  | **Lucio** | SP | 58 | H | EP | SA | S | C | P | P | R | M | R | A |
| 280 | x | x | **Lucques** | FRA | 5 | H | EL | A | SA | C | P | P | S | L | G | P |
| 281 |  | x | Lumbardeska | HRV | 268 | H | EP | SA | S | C | P | R | R | H | R | P |
| 282 |  | x | Lumiaro | ITA | 348 | VH | EP | SA | S | C | P | P | R | H | R | P |
| 283 | x |  | Maarri | SYR | 202 | M | O | SA | SA | C | P | R | S | M | R | P |
| 284 | x |  | Macho de Jaén | SP | 12 | H | EL | A | S | C | P | P | R | M | R | A |
| 285 | x | x | **Machorrón** | SP | 226 | M | S | SA | S | C | R | R | R | M | G | P |
| 286 |  | x | Madonna dell 'impruneta | ITA | 292 | M | EP | SA | S | C | R | P | R | H | R | P |
| 287 |  | x | *Madural-208* | PRT | 363 | M | EP | A | S | A | R | P | R | M | R | P |
| 288 | x |  | Mahati-1010 | SYR | 241 | VH | EP | SA | S | A | R | R | R | M | R | P |
| 289 |  | x | *Mahati-615* | SYR |  | No Data | | | | | | | | | | |
| 290 | x |  | Mahati-846 | SYR | 245 | VH | O | SA | S | C | P | T | R | H | R | P |
| 291 |  | x | Maiatica di Ferrandina | ITA | 247 | M | EP | SA | S | A | R | P | S | M | R | P |
| 292 | x |  | Majhol-1059 | SYR | 163 | M | EP | A | S | C | P | P | S | L | G | P |
| 293 | x |  | Majhol-1063 | SYR |  | No Data | | | | | | | | | | |
| 294 | x |  | Majhol-1122 | SYR | 222 | M | O | S | S | C | R | P | R | L | G | P |
| 295 | x |  | Majhol-152 | SYR | 197 | M | EP | S | S | C | R | R | R | M | R | P |
| 296 |  | x | Mançanilha Algarvia | PRT | 362 | VH | O | SA | S | C | R | T | SC | M | R | P |
| 297 |  | x | Mantonica | ITA | 9 | H | EL | A | S | A | P | P | R | M | R | P |
| 298 | x |  | Manzanil de Piedra Buena | SP |  | No Data | | | | | | | | | | |
| 299 | x | x | **Manzanilla Cacereña** | SP | 185 | M | EP | SA | S | C | P | P | R | M | R | P |
| 300 | x | x | **Manzanilla de Abla^a^** | SP | 107 | H | O | S | S | C | R | R | R | M | R | P |
| 301 | x | x | **Manzanilla de Agua** | SP | 204 | M | O | SA | S | A | R | P | R | M | G | P |
| 302 | x |  | **Manzanilla de Almería** | SP | 204 | M | O | SA | S | A | R | P | R | M | G | P |
| 303 | x | x | **Manzanilla de Hellín** | SP | 109 | H | O | S | S | C | R | T | R | H | R | P |
| 304 | x |  | *Manzanilla de Lorca-808 -1* | SP | 125 | L | EP | SA | S | A | R | P | S | M | R | P |
| 305 | x |  | *Manzanilla de Lorca-808* | SP |  | No Data | | | | | | | | | | |
| 306 | x |  | *Manzanilla de Lorca-809* | SP |  | No Data | | | | | | | | | | |
| 307 | x | x | **Manzanilla de Montefrío** | SP | 244 | VH | O | SA | S | C | P | R | SC | H | R | P |
| 308 | x |  | Manzanilla de San Vicente | MEX | 107 | H | O | S | S | C | R | R | R | M | R | P |
| 309 | x | x | **Manzanilla de Sevilla** | SP | 84 | H | O | SA | S | A | R | P | R | M | R | P |
| 310 | x |  | **Manzanilla del Piquito** | SP | 187 | M | EP | SA | S | C | P | R | R | M | R | P |
| 311 | x |  | *Manzanilla del Piquito-269* | SP |  | No Data | | | | | | | | | | |
| 312 | x |  | **Manzanilla Prieta** | SP | 215 | M | O | SA | S | C | R | R | R | H | R | A |
| 313 | x |  | **Manzanillera de Huércal Overa** | SP | 163 | M | EP | SA | SA | A | R | R | S | M | R | P |
| 314 | x |  | Manzanillo de Cabra | SP | 67 | H | EP | SA | S | C | R | P | R | M | R | P |
| 315 |  | x | Maraki | EGY | 306 | VH | O | SA | S | C | R | R | R | M | R | A |
| 316 |  | x | Maremmano | ITA | 366 | H | EL | SA | S | C | R | P | R | M | R | P |
| 317 | x |  | **Mari** | IRA | 144 | M | EL | A | S | A | P | R | R | M | R | P |
| 318 | x | x | Masabi | SYR | 242 | VH | EP | SA | S | C | P | R | R | M | R | P |
| 319 | x | x | **Mastoidis** | GRC | 155 | M | EP | A | SA | C | P | P | S | M | R | P |
| 320 | x | x | **Maurino** | ITA | 188 | M | EP | SA | S | C | R | R | R | M | R | P |
| 321 | x | x | **Mavreya^a^** | GRC | 123 | L | EP | SA | SA | C | P | P | S | M | G | P |
| 322 | x | x | Mawi | SYR | 30 | H | EP | A | S | A | P | P | R | M | R | P |
| 323 | x |  | **Megaritiki** | GRC | 111 | L | EL | A | SA | C | P | P | S | H | R | A |
| 324 |  | x | Meloky | EGY | 248 | H | EP | SA | S | A | R | P | SC | M | R | P |
| 325 | x |  | **Memecik** | TUR | 72 | H | EP | SA | S | C | R | R | SC | M | G | P |
| 326 | x |  | **Menya** | SP | 148 | M | EL | SA | SA | C | P | R | S | M | G | P |
| 327 | x |  | **Merhavia** | ISR | 9 | H | EL | A | S | A | P | P | R | M | R | P |
| 328 | x |  | **Meski** | TUN | 82 | H | O | SA | S | A | P | P | R | M | G | A |
| 329 |  | x | Meslala | SP | 282 | H | EP | A | S | C | R | R | R | M | R | P |
| 330 |  | x | *Mesyaf-641* | SYR | 319 | VH | EL | SA | S | A | P | P | R | M | R | P |
| 331 |  | x | *Mesyaf-662* | SYR |  | No Data | | | | | | | | | | |
| 332 |  | x | Mignolo Cerretano | ITA | 258 | M | EP | A | S | C | P | R | S | L | R | P |
| 333 |  | x | Mignolo | ITA | 336 | M | EP | SA | S | C | R | P | R | L | G | P |
| 334 |  | x | Minekiri | SYR | 257 | H | EP | SA | SA | C | P | P | R | M | R | P |
| 335 | x |  | Mision Moojeski | USA | 61 | H | EP | SA | S | C | P | R | R | M | R | P |
| 336 | x |  | **Mixani** | ALB | 181 | M | EP | SA | S | A | R | P | R | M | R | A |
| 337 |  | x | Mohazam Abou Satl | SYR | 270 | VH | EL | A | SA | C | P | R | SC | H | R | P |
| 338 | x |  | Mollar Basto | SP | 46 | H | EP | SA | SA | C | R | P | R | M | G | P |
| 339 | x | x | **Mollar de Cieza** | SP | 187 | M | EP | SA | S | C | P | R | R | M | R | P |
| 340 | x | x | **Moraiolo** | ITA | 201 | M | O | SA | SA | A | R | R | R | H | R | P |
| 341 |  | x | Morchiaio | ITA | 255 | M | EP | SA | S | C | R | P | R | M | R | P |
| 342 |  | x | Morchione | ITA | 99 | H | O | SA | S | C | R | R | R | M | R | P |
| 343 |  | x | Morcone | ITA | 335 | L | EP | A | S | C | R | P | R | M | R | P |
| 344 |  | x | Morello a punta | ITA | 279 | H | EP | SA | S | C | R | P | R | L | R | P |
| 345 |  | x | Moresca | ITA | 242 | VH | EP | SA | S | C | P | R | R | M | R | P |
| 346 | x |  | Morisca de Mancor | SP | 85 | H | O | SA | S | A | R | P | SC | M | R | A |
| 347 | x | x | **Morisca** | SP | 32 | H | EP | A | S | C | P | P | R | H | R | P |
| 348 | x | x | **Morona** | SP | 70 | H | EP | SA | S | C | R | R | R | M | R | P |
| 349 | x | x | **Morrut** | SP | 54 | H | EP | SA | S | A | R | P | S | M | R | P |
| 350 | x |  | *Morrut-607* | SP |  | No Data | | | | | | | | | | |
| 351 |  | x | Mortellino | ITA | 314 | M | O | S | S | C | R | R | S | M | G | P |
| 352 |  | x | Nasitana Frutto Grosso | ITA | 349 | H | EP | SA | S | A | R | P | S | H | R | P |
| 353 | x |  | Nasuhi | ISR |  | No Data | | | | | | | | | | |
| 354 |  | x | *Neb Jmel-283* | TUN | 284 | M | EP | A | S | C | P | P | S | M | R | P |
| 355 |  | x | *Neb J'mel-452* | ALG | 302 | H | EL | A | SA | C | P | P | R | L | R | P |
| 356 | x |  | Negral de Sabiñan | SP | 71 | H | EP | SA | S | C | R | R | S | M | R | P |
| 357 |  | x | *Negral de Sabiñan-255* | SP | 70 | H | EP | SA | S | C | R | R | R | M | R | P |
| 358 | x | x | **Negrillo de Arjona** | SP | 186 | M | EP | SA | S | C | P | R | R | H | R | P |
| 359 | x | x | **Negrillo de Estepa** | SP | 207 | M | O | SA | S | A | R | R | S | M | R | P |
| 360 | x | x | **Negrillo de Iznalloz** | SP | 185 | M | EP | SA | S | C | P | P | R | M | R | P |
| 361 | x |  | **Negrillo de la Carlota** | SP | 185 | M | EP | SA | S | C | P | P | R | M | R | P |
| 362 | x | x | **Negrillo Redondo** | SP | 213 | M | O | SA | S | C | R | P | R | M | G | P |
| 363 |  | x | Negrita | PRT | 284 | M | EP | A | S | C | P | P | S | M | R | P |
| 364 | x |  | **Negro del Carpio** | SP | 70 | H | EP | SA | S | C | R | R | R | M | R | P |
| 365 |  | x | Nerba | ITA | 4 | H | EL | A | SA | C | P | P | R | M | R | P |
| 366 | x |  | Nevadillo Blanco de Jaén | SP | 59 | H | EP | SA | S | C | P | P | R | M | R | P |
| 367 | x |  | **Nevadillo de Santisteban Pto** | SP | 70 | H | EP | SA | S | C | R | R | R | M | R | P |
| 368 | x | x | **Nevado Azul** | SP | 4 | H | EL | A | SA | C | P | P | R | M | R | P |
| 369 | x | x | **Nevado Basto** | SP | 28 | H | EP | A | SA | C | P | P | R | M | G | P |
| 370 | x | x | **Nevado Rizado** | SP | 187 | M | EP | SA | S | C | P | R | R | M | R | P |
| 371 |  | x | Nocellara del Belice | ITA | 272 | H | O | SA | S | C | R | R | SC | H | R | P |
| 372 |  | x | Nocellara Etnea | ITA | 20 | H | EL | SA | SA | C | P | R | R | H | R | P |
| 373 |  | x | Nociara | ITA | 138 | M | EL | A | SA | C | P | P | R | M | R | P |
| 374 | x |  | **Oblica** | HRV | 73 | H | EP | SA | S | C | R | T | R | H | R | P |
| 375 | x | x | **Ocal** | SP | 20 | H | EL | SA | SA | C | P | R | R | H | R | P |
| 376 | x |  | *Ocal-25* | SP | 106 | H | O | S | S | C | R | R | R | M | G | P |
| 377 | x |  | *Ocal-427* | SP | 187 | M | EP | SA | S | C | P | R | R | M | R | P |
| 378 |  | x | Ogliarola del Bradano | ITA | 120 | L | EP | A | S | C | P | P | S | M | R | P |
| 379 |  | x | Ogliarola del Vulture | ITA | 285 | L | EP | A | S | C | P | P | S | L | R | P |
| 380 | x | x | **Ojo de Liebre** | SP | 165 | M | EP | A | S | C | P | R | R | M | R | A |
| 381 |  | x | Olivastra di Montalcino | ITA | 106 | H | O | S | S | C | R | R | R | M | G | P |
| 382 |  | x | Olivastra di Populonia | ITA | 339 | L | EP | SA | S | A | R | P | S | H | R | P |
| 383 | x | x | **Olivo de Mancha Real** | SP | 2 | H | EL | A | SA | A | R | P | R | H | R | P |
| 384 | x |  | Olivo de Maura | SP | 47 | H | EP | SA | SA | C | R | R | S | M | R | P |
| 385 |  | x | Olivo del Mulino | ITA | 67 | H | EP | SA | S | C | R | P | R | M | R | P |
| 386 |  | x | Olivo di Casavecchia | ITA | 263 | H | EL | A | S | C | P | P | R | M | R | P |
| 387 |  | x | Olivo di Mandanici | ITA | 259 | M | EL | A | S | C | P | P | R | L | R | P |
| 388 |  | x | Olivo di San Lorenzo | ITA | 188 | M | EP | SA | S | C | R | R | R | M | R | P |
| 389 |  | x | Ornellaia | ITA | 4 | H | EL | A | SA | C | P | P | R | M | R | P |
| 390 |  | x | Ottobratica | ITA | 325 | L | EL | A | SA | C | P | P | S | M | R | P |
| 391 | x |  | **Ouslati** | TUN | 126 | L | EP | SA | S | C | P | P | S | M | G | P |
| 392 | x |  | Pajarera | SP |  | No Data | | | | | | | | | | |
| 393 | x |  | **Pajarero** | SP | 19 | H | EL | SA | SA | C | P | P | R | M | R | P |
| 394 | x | x | **Palomar** | SP | 183 | M | EP | SA | S | B | P | R | R | M | R | A |
| 395 |  | x | Passulunara | ITA | 24 | H | EL | SA | S | C | P | R | R | M | R | P |
| 396 | x | x | **Patronet^a^** | SP | 141 | M | EL | A | SA | C | P | R | S | M | G | A |
| 397 | x |  | **Pavo** | SP | 1 | H | EL | A | SA | A | P | P | R | H | R | A |
| 398 | x |  | **Pendolino** | ITA | 159 | M | EP | A | S | A | R | P | S | L | G | P |
| 399 |  | x | *Pendolino-162* | ITA | 354 | L | EP | SA | S | A | R | P | S | M | G | P |
| 400 | x |  | **Pequeña de Casas Ibañez** | SP | 131 | L | O | S | S | C | P | R | S | L | G | A |
| 401 | x |  | Perafort | SP | 225 | M | O | S | S | C | R | T | S | L | G | P |
| 402 |  | x | Piangente | ITA | 337 | H | EP | SA | S | A | R | P | S | L | R | P |
| 403 | x | x | **Picholine Marocaine** | MOR | 162 | M | EP | A | S | C | P | P | R | M | R | P |
| 404 | x | x | **Picholine** | FRA | 143 | M | EL | A | S | A | P | P | R | M | G | P |
| 405 | x | x | **Pico Limón de Grazalema** | SP | 27 | H | EL | S | S | C | P | P | R | M | R | P |
| 406 | x |  | **Pico Limón** | SP | 45 | H | EP | SA | SA | C | P | R | R | M | R | P |
| 407 | x |  | **Picual de Almería** | SP | 43 | H | EP | SA | SA | A | R | P | R | M | R | P |
| 408 | x | x | **Picual** | SP | 36 | H | EP | A | S | C | P | R | SC | M | R | A |
| 409 | x |  | *Picual-1470* | SP |  | No Data | | | | | | | | | | |
| 410 | x |  | *Picual-9* | SP |  | No Data | | | | | | | | | | |
| 411 | x |  | Picudo Blanco de Estepa | SP | 61 | H | EP | SA | S | C | P | R | R | M | R | P |
| 412 | x | x | **Picudo** | SP | 4 | H | EL | A | SA | C | P | P | R | M | R | P |
| 413 |  | x | Pidicuddara | ITA | 274 | M | O | SA | S | C | R | R | R | M | R | P |
| 414 |  | x | Pikrolia | GRC | 358 | L | EP | SA | S | C | R | R | R | M | R | A |
| 415 | x |  | *Piñonera-76* | SP | 189 | M | EP | SA | S | C | R | R | S | M | R | P |
| 416 | x | x | **Plementa Bjelica^a^** | HRV | 195 | M | EP | S | S | C | R | P | R | M | R | P |
| 417 | x |  | Polinizador | MEX | 92 | H | O | SA | S | C | P | R | R | M | R | A |
| 418 | x |  | Pulazeqin | ALB | 104 | H | O | S | S | A | R | R | R | L | R | A |
| 419 |  | x | Puntoza | HRV | 64 | H | EP | SA | S | C | P | R | SC | M | G | P |
| 420 | x |  | Rachati | GRC | 229 | VH | EL | A | S | A | P | P | R | M | R | P |
| 421 |  | x | *Rachati-181* | GRC | 359 | L | EP | A | S | C | P | R | R | M | R | P |
| 422 | x |  | Racimal de Jaén | SP | 175 | M | EP | SA | SA | C | P | P | R | M | R | A |
| 423 | x | x | **Racimal** | SP | 166 | M | EP | A | S | C | P | R | R | M | R | P |
| 424 | x | x | **Rapasayo** | SP | 164 | M | EP | A | S | C | P | R | R | H | R | P |
| 425 |  | x | Ravece | ITA | 83 | H | O | SA | S | A | R | P | R | H | R | P |
| 426 |  | x | Razzaio | ITA | 254 | L | EP | SA | S | C | R | R | R | M | R | P |
| 427 | x |  | Real Sevillana | SP | 14 | H | EL | A | S | C | P | R | SC | M | R | P |
| 428 | x | x | **Rechino** | SP | 199 | M | O | A | SA | C | P | P | R | M | R | A |
| 429 |  | x | Redondal | PRT | 364 | H | EP | SA | SA | C | R | R | R | M | R | P |
| 430 | x |  | Redondilla de Grazalema | SP | 231 | VH | EL | A | S | C | P | R | R | M | R | P |
| 431 | x |  | **Redondilla de Logroño** | SP | 224 | M | O | S | S | C | R | R | R | M | R | P |
| 432 | x |  | Reixonenca | SP | 108 | H | O | S | S | C | R | R | S | L | G | P |
| 433 |  | x | Remmani | LBN | 369 | VH | S | S | S | C | R | T | SC | M | R | A |
| 434 |  | x | Ronde de la Ménara | MOR | 351 | VH | O | SA | S | C | R | R | SC | H | R | P |
| 435 |  | x | Ronde de Miliana | ALG | 273 | VH | EP | SA | S | C | R | R | SC | M | R | A |
| 436 | x |  | **Rosciola** | ITA | 122 | L | EP | SA | SA | A | R | P | R | M | R | P |
| 437 |  | x | Rossellino Cerretano | ITA | 265 | M | EL | A | SA | C | P | P | S | L | R | P |
| 438 |  | x | Rossellino | ITA | 274 | M | O | SA | S | A | R | R | R | M | R | P |
| 439 |  | x | Rossello | ITA | 254 | L | EP | SA | S | C | R | R | R | M | R | P |
| 440 |  | x | Rougette de Mitidja | ALG | 284 | M | EP | A | S | C | P | P | S | M | R | P |
| 441 | x |  | **Rowghani** | IRA | 91 | H | O | SA | S | C | P | R | R | L | R | P |
| 442 | x |  | **Royal de Calatayud** | SP | 180 | M | EP | SA | S | A | P | P | R | M | R | P |
| 443 | x |  | *Royal de Calatayud-47* | SP | 179 | M | EP | SA | S | A | P | P | R | L | G | P |
| 444 | x | x | **Royal de Cazorla** | SP | 18 | H | EL | SA | SA | A | P | P | S | M | R | P |
| 445 | x |  | Royal de Sabiñan | SP | 88 | H | O | SA | S | B | R | R | S | M | R | P |
| 446 | x | x | **Sabatera** | SP | 216 | M | O | SA | S | C | R | R | R | M | G | P |
| 447 | x |  | Safrawi | SYR | 78 | H | EP | S | S | C | P | R | R | M | R | P |
| 448 | x |  | Salgar Redondo | SP | 96 | H | O | SA | S | C | R | R | R | H | R | P |
| 449 |  | x | Salicino | ITA | 338 | H | O | S | S | C | R | R | R | L | G | P |
| 450 | x | x | **Salonenque** | FRA | 75 | H | EP | S | S | A | R | P | S | M | G | A |
| 451 |  | x | Samo | SLV | 296 | VH | EP | SA | S | C | P | R | SC | H | R | P |
| 452 |  | x | San Francesco | ITA | 262 | VH | EL | A | S | C | P | P | R | M | R | P |
| 453 | x |  | Sandalio | SP | 217 | M | O | SA | S | C | R | R | S | M | R | P |
| 454 | x | x | **Sant Agostino** | ITA | 63 | H | EP | SA | S | C | P | R | SC | H | G | P |
| 455 |  | x | Santa Martinenga | ITA | 367 | L | O | SA | S | C | R | T | R | H | G | P |
| 456 |  | x | Sayali | TUN | 321 | M | EP | SA | SA | C | P | R | SC | L | R | A |
| 457 | x |  | Sayfi | SYR | 61 | H | EP | SA | S | C | P | R | R | M | R | P |
| 458 |  | x | Sebhawy | EGY | 372 | M | EP | A | S | B | P | R | R | L | R | P |
| 459 | x |  | Selvatico | ITA |  | No Data | | | | | | | | | | |
| 460 | x |  | Sevillana de Abla | SP | 238 | VH | EP | A | S | C | P | R | R | M | R | P |
| 461 | x | x | **Sevillano de Jumilla** | SP | 79 | H | EP | S | S | C | R | R | R | L | G | P |
| 462 | x | x | **Sevillenca** | SP | 152 | M | EL | SA | S | C | P | P | R | M | G | P |
| 463 | x |  | Shami | SYR | 62 | H | EP | SA | S | C | P | R | S | M | R | P |
| 464 | x |  | **Shengue** | IRA | 242 | VH | EP | SA | S | C | P | R | R | M | R | P |
| 465 |  | x | Simjaca | HRV | 309 | VH | EP | SA | S | C | R | R | SC | M | G | P |
| 466 | x |  | Sin nombre | ITA |  | No Data | | | | | | | | | | |
| 467 | x |  | Sinop | TUR | 129 | L | O | SA | S | C | R | T | R | M | R | P |
| 468 |  | x | Sinopolese | ITA |  | No Data | | | | | | | | | | |
| 469 |  | x | Sivigliana da Olio | ITA | 352 | L | O | SA | S | A | R | P | S | L | R | A |
| 470 | x |  | **Sollana** | SP | 16 | H | EL | SA | SA | A | P | P | R | M | R | P |
| 471 | x |  | Sorani | SYR | 180 | M | EP | SA | S | A | P | P | R | M | R | P |
| 472 |  | x | Souidi | ALG | 287 | L | EP | SA | S | C | R | P | S | M | R | P |
| 473 | x |  | Sourani Red | SYR | 117 | L | EL | S | S | C | P | P | S | M | R | P |
| 474 |  | x | Štorta | SLV | 9 | H | EL | A | S | A | P | P | R | M | R | P |
| 475 |  | x | Sukkare | SYR |  | No Data | | | | | | | | | | |
| 476 |  | x | Tabelout | ALG | 290 | M | EL | A | S | A | P | P | S | M | R | P |
| 477 | x |  | **Tanche**^12^ | FRA | 98 | H | O | SA | S | C | R | R | R | M | G | P |
| 478 | x |  | *Tanche-74* | FRA |  | No Data | | | | | | | | | | |
| 479 |  | x | Tarabelsi | SYR | 294 | VH | EL | SA | SA | C | P | P | R | H | R | P |
| 480 |  | x | Tebabs | SYR | 242 | VH | EP | SA | S | C | P | R | R | M | R | P |
| 481 |  | x | Teffah | ALG | 298 | VH | O | S | S | C | R | R | SC | M | R | A |
| 482 | x |  | **Tempranillo de Yeste** | SP | 160 | M | EP | A | S | A | R | P | S | M | R | P |
| 483 |  | x | *Tempranillo de Yeste-274* | SP | 54 | H | EP | SA | S | A | R | P | S | M | R | P |
| 484 | x |  | Temprano | SP | 26 | H | EL | SA | S | C | R | T | SC | H | R | P |
| 485 | x |  | *Toffahi-1000* | SYR | 77 | H | EP | S | S | C | P | P | SC | M | R | P |
| 486 | x |  | **Toffahi** | EGY | 243 | VH | O | SA | S | C | P | R | S | L | R | A |
| 487 |  | x | *Toffahi-486* | EGY | 371 | VH | O | SA | S | C | P | T | SC | M | R | P |
| 488 |  | x | *Toffahi-621* | SYR | 375 | H | EP | A | SA | C | P | P | R | H | R | P |
| 489 |  | x | Tonda Iblea | ITA | 329 | H | O | S | S | C | R | R | SC | H | R | A |
| 490 | x |  | Toruno | SP | 89 | H | O | SA | S | C | P | P | R | H | R | P |
| 491 |  | x | Tounsi-461 | TUN |  | No Data | | | | | | | | | | |
| 492 |  | x | Trillo | ITA | 295 | M | EP | SA | S | A | R | P | S | L | R | P |
| 493 | x |  | *Trylia -992* | SYR |  | No Data | | | | | | | | | | |
| 494 | x |  | Ulliri i Bardhe i Tiranes | ALB | 80 | H | EP | S | S | C | R | R | R | M | R | P |
| 495 | x |  | Ulliri i Kuq | ALB | 202 | M | O | SA | SA | C | P | R | S | M | R | P |
| 496 |  | x | Unkown-OT2-537 | MOR | 311 | L | O | SA | S | A | R | P | S | M | R | P |
| 497 |  | x | Unkown-OZ1-538 | MOR | 312 | L | EL | A | SA | C | P | R | S | L | R | P |
| 498 |  | x | Unkown-VS1-544 | MOR | 258 | M | EP | A | S | C | P | P | S | L | R | P |
| 499 |  | x | Unkown-VS2-545 | MOR | 128 | L | O | SA | S | C | R | R | S | M | R | P |
| 500 |  | x | Unkown-VS5-547 | MOR | 313 | M | EL | A | S | C | P | R | S | M | R | P |
| 501 | x | x | **Uovo di Piccione** | ITA | 192 | VH | O | SA | S | C | R | T | SC | M | R | A |
| 502 | x |  | **Uslu** | TUR | 10 | H | EL | A | S | C | P | P | R | H | R | P |
| 503 |  | x | Vaddarica | ITA | 355 | H | EP | SA | S | A | P | P | R | M | G | P |
| 504 | x |  | **Valanolia** | GRC | 52 | H | EP | SA | S | A | R | P | R | L | G | P |
| 505 | x |  | **Vallesa** | SP | 171 | M | EP | SA | SA | A | R | P | S | M | R | P |
| 506 | x |  | **Vaneta** | SP | 113 | L | EL | SA | SA | C | P | R | R | M | G | P |
| 507 | x | x | **Varudo** | SP | 157 | M | EP | A | SA | C | P | R | R | M | R | P |
| 508 |  | x | *Varudo-275* | SP | 281 | H | EP | A | SA | C | P | P | R | M | R | P |
| 509 |  | x | Vasilikada | GRC | 360 | H | EP | A | S | B | P | R | R | H | R | P |
| 510 |  | x | Velika Lastovka | HRV | 310 | VH | EL | A | S | A | R | P | R | M | R | P |
| 511 | x | x | **Vera** | SP | 86 | H | O | SA | S | A | R | R | R | M | G | P |
| 512 | x |  | Verdal de Manresa | SP | 59 | H | EP | SA | S | C | P | P | R | M | R | P |
| 513 | x | x | **Verdala** | SP | 146 | M | EL | A | S | C | P | P | R | M | R | P |
| 514 | x | x | **Verdale** | FRA | 219 | M | O | S | S | A | R | P | R | M | G | A |
| 515 | x |  | Verde Verdelho | PRT | 193 | M | EP | S | S | C | P | R | S | H | R | P |
| 516 | x | x | **Verdial de Badajoz** | SP | 31 | H | EP | A | S | A | R | P | R | M | R | P |
| 517 | x |  | **Verdial de Cádiz** | SP | 35 | H | EP | A | S | C | P | R | SC | H | R | A |
| 518 | x | x | **Verdial de Huévar** | SP | 83 | H | O | SA | S | A | R | P | R | H | R | P |
| 519 | x |  | **Verdial de Vélez-Málaga** | SP | 210 | M | O | SA | S | C | P | R | R | M | G | P |
| 520 | x |  | *Verdial de Vélez-Málaga-51* | SP | 138 | M | EL | A | SA | C | P | P | R | M | R | P |
| 521 |  | x | Verdial transmontana | PRT | 34 | H | EP | A | S | C | P | R | R | M | R | P |
| 522 | x | x | **Verdiell** | SP | 125 | L | EP | SA | S | A | R | P | S | M | R | P |
| 523 | x | x | **Villalonga** | SP | 95 | H | O | SA | S | C | P | T | R | M | G | P |
| 524 | x |  | Vinyols | SP | 228 | M | S | S | S | C | R | P | S | M | G | P |
| 525 | x |  | Wardan | EGY | 182 | M | EP | SA | S | A | R | P | R | M | R | P |
| 526 |  | x | Wateken | EGY | 370 | VH | EP | A | S | A | R | P | R | M | R | P |
| 527 |  | x | Zael Al Muhra | SYR | 10 | H | EL | A | S | C | P | P | R | H | R | P |
| 528 | x |  | *Zaity -788* | SYR |  | No Data | | | | | | | | | | |
| 529 | x | x | **Zaity** | SYR | 196 | M | EP | S | S | C | R | R | R | H | R | P |
| 530 | x |  | **Zalmati** | TUN | 116 | L | EL | S | S | C | P | P | S | M | G | P |
| 531 |  | x | *Zalmati-299* | TUN | 323 | L | EL | SA | S | A | R | P | S | M | R | P |
| 532 | x |  | **Zard** | IRA | 105 | H | O | S | S | C | P | R | R | M | R | P |
| 533 | x | x | **Zarza** | SP | 198 | M | O | A | SA | C | R | R | SC | H | R | A |
| 534 |  | x | Zeboudj boudoudane | ALG | 20 | H | EL | SA | SA | C | P | R | R | H | R | P |
| 535 |  | x | Zeletni | ALG | 297 | M | EL | SA | SA | C | P | P | R | M | R | P |

^1^ Authentic cultivars in bold, ^a^ not authenticated by Trujillo et al. (2014).

^2^ ALB, Albania; ALG, Algeria; ARG, Argentina, CYP, Cyprus; HRV, Croatia; EGY, Egypt; FRA, France; GRC, Greece; ISR, Israel; ITA, Italy; IRA, Iran; LBN, Lebanon; MAR, Morocco; MEX, Mexico; PRT, Portugal; ESP, Spain; SYR, Syria; TUN, Tunisia; TUR, Turkey; USA, United States of America.

^3^ Weight: low = L (< 0.3 g); medium = M (0.3-0.45 g); high = H (0.45-0.7 g); very high = VH (> 0.7 g).

^4^ Shape in position A: spherical = S (length/width < 1.4); ovoid = O (length/width 1.4-1.8); elliptic = EP (length/width 1.8-2.2); elongated = EL (length/width > 2.2).

^5^ Symmetry in position A: symmetric = S; slightly asymmetric = SA; asymmetric = A.

^6^ Symmetry of position B: symmetric = S; slightly asymmetric = SA.

^7^ Position of the maximum transversal diameter in position B: towards base = B; central = C; towards apex = A.

^8^ Shape of apex in position A: pointed = P; rounded = R.

^9^ Shape of base in position A: pointed = P; truncated = T; rounded = R.

^10^ Rugosity of surface: smooth = S; rough = R; scabrous= SC.

^11^ Number of grooves on basal end: low = L (< 7); medium = M (7- 10); high = H (> 10).

^12^ Distribution of grooves on basal endo: regular = R; grouped around the suture = G.

^13^ Presence of mucro: present = P; absent = A.
