# Supplementary material for: Characterization of Worldwide Olive Germplasm Banks of Marrakech (Morocco) and Córdoba (Spain): Towards management and use of olive germplasm in breeding programs
Source: PLoS One. 2019 Oct 17;14(10):e0223716. doi: 10.1371/journal.pone.0223716 (PMC6797134; doi:10.1371/journal.pone.0223716)
Supplement: S5 Table — Number of alleles (Na), expected (He) and observed (Ho) heterozygosity, allelic richness (Ar). (DOCX) [file pone.0223716.s005.docx]

**S5 Table.** Summary of genetic parameters of different loci regarding shared genotypes and those specific to each collection. Number of alleles (Na), expected (He) and observed (Ho) heterozygosity, allelic richness (Ar).

| **N** | **Loci** | **Shared genotypes (130)** | | | |  | **Specific to WOGBM (272)** | | | |  | **Specific to WOGBC (270)** | | | |
| --- | --- | --- | --- | --- | --- | --- | --- | --- | --- | --- | --- | --- | --- | --- | --- |
|  |  | **Na** | **He** | **Ho** | **Ar^1^** |  | **Na** | **He** | **Ho** | **Ar^1^** |  | **Na** | **He** | **Ho** | **Ar^1^** |
| **1** | DCA01 | 10 | 0.650 | 0.838 | 7.2 |  | 19 | 0.605 | 0.669 | 10.4 |  | 13 | 0.597 | 0.732 | 6.8 |
| **2** | DCA03 | 10 | 0.829 | 0.954 | 9.2 |  | 13 | 0.855 | 0.879 | 9.6 |  | 15 | 0.853 | 0.926 | 11.7 |
| **3** | DCA04 | 19 | 0.780 | 0.636 | 15.2 |  | 32 | 0.873 | 0.643 | 21.2 |  | 26 | 0.830 | 0.658 | 16.4 |
| **4** | DCA05 | 9 | 0.326 | 0.333 | 7.5 |  | 12 | 0.551 | 0.563 | 10.2 |  | 10 | 0.425 | 0.410 | 9.0 |
| **5** | DCA08 | 12 | 0.782 | 0.566 | 10.3 |  | 20 | 0.845 | 0.868 | 14.0 |  | 17 | 0.815 | 0.649 | 12.3 |
| **6** | DCA09 | 16 | 0.850 | 0.969 | 13.5 |  | 25 | 0.893 | 0.949 | 18.8 |  | 22 | 0.875 | 0.937 | 17.4 |
| **7** | DCA10 | 18 | 0.781 | 0.217 | 15.4 |  | 34 | 0.880 | 0.367 | 23.3 |  | 32 | 0.826 | 0.190 | 21.7 |
| **8** | DCA11 | 18 | 0.805 | 0.923 | 13.8 |  | 22 | 0.833 | 0.879 | 16.2 |  | 24 | 0.831 | 0.922 | 15.9 |
| **9** | DCA15 | 5 | 0.523 | 0.292 | 4.0 |  | 6 | 0.613 | 0.540 | 5.2 |  | 7 | 0.497 | 0.193 | 4.8 |
| **10** | DCA16 | 19 | 0.849 | 0.985 | 14.7 |  | 32 | 0.880 | 0.956 | 19.2 |  | 30 | 0.850 | 0.948 | 15.9 |
| **11** | DCA18 | 10 | 0.808 | 0.923 | 9.2 |  | 17 | 0.833 | 0.912 | 13.2 |  | 16 | 0.825 | 0.919 | 12.8 |
| **12** | EMO90 | 7 | 0.590 | 0.643 | 6.0 |  | 9 | 0.693 | 0.739 | 7.4 |  | 10 | 0.668 | 0.732 | 7.8 |
| **13** | GAPU59 | 8 | 0.586 | 0.685 | 6.4 |  | 10 | 0.624 | 0.563 | 7.4 |  | 10 | 0.645 | 0.626 | 6.6 |
| **14** | GAPU71A | 7 | 0.445 | 0.535 | 5.4 |  | 15 | 0.465 | 0.548 | 7.9 |  | 8 | 0.423 | 0.459 | 5.4 |
| **15** | GAPU71B | 6 | 0.797 | 0.954 | 5.5 |  | 9 | 0.808 | 0.882 | 7.6 |  | 8 | 0.805 | 0.926 | 7.2 |
| **16** | GAPU101 | 9 | 0.829 | 0.985 | 8.7 |  | 13 | 0.858 | 0.923 | 10.0 |  | 13 | 0.840 | 0.967 | 9.9 |
| **17** | GAPU103A | 16 | 0.786 | 0.708 | 12.6 |  | 26 | 0.866 | 0.849 | 16.9 |  | 26 | 0.833 | 0.756 | 16.3 |
| **18** | UDO99-11 | 10 | 0.829 | 0.992 | 9.2 |  | 13 | 0.855 | 0.956 | 10.3 |  | 14 | 0.827 | 0.926 | 9.9 |
| **19** | UDO99-17 | 5 | 0.774 | 0.791 | 5.0 |  | 7 | 0.782 | 0.783 | 6.8 |  | 6 | 0.787 | 0.765 | 5.7 |
| **20** | UDO99-43 | 19 | 0.863 | 0.892 | 14.2 |  | 24 | 0.879 | 0.848 | 18.2 |  | 22 | 0.881 | 0.878 | 16.8 |
| **Mean** |  | **11.65** | **0.724^a^** | **0.741** | **9.7^a^** |  | **17.90** | **0.774^b^** | **0.765** | **12.7^a^** |  | **16.45** | **0.746^ab^** | **0.745** | **11.5^a^** |
| **Total** |  | **233** |  |  | **194** |  | **358** |  |  | **254** |  | **329** |  |  | **230** |

^1^Computed at G value of 130

^a, b^ index of significance at p-value < 0.05.
