# Supplementary material for: Characterization of Worldwide Olive Germplasm Banks of Marrakech (Morocco) and Córdoba (Spain): Towards management and use of olive germplasm in breeding programs
Source: PLoS One. 2019 Oct 17;14(10):e0223716. doi: 10.1371/journal.pone.0223716 (PMC6797134; doi:10.1371/journal.pone.0223716)
Supplement: S6 Table — (DOCX) [file pone.0223716.s006.docx]

**S6 Table.** Cases of cultivars showing molecular variants with their cultivation area, the number of molecular variants (No. MV) in both collections and the number of distinct alleles.

| **Cultivars** | **Cultivation area** | **No. MV** | | | **No. Distinct alleles range** |
| --- | --- | --- | --- | --- | --- |
|  |  | **Total** | **WOGBM** | **WOGBC** |  |
| Adkam | Syria | 2 | 1 | 1 | 1 |
| Alameño de Montilla | Spain | 3 | 1 | 3 | 1-2 |
| Alfafara | Spain | 2 |  | 2 | 2 |
| Atounsi Setif | Algeria | 2 | 2 |  | 2 |
| Beladi | Lebanon | 8 | 8 | 1 | 1-3 |
| Belluti | Turkey | 2 |  | 2 | 1 |
| Blanqueta | Spain | 2 | 1 | 2 | 1 |
| Bolvino | Spain | 3 | 1 | 3 | 1-2 |
| Bosana | Spain | 3 | 2 | 1 | 2 |
| Buga | HRV | 3 | 2 | 1 | 1-2 |
| Callosina | Spain | 2 | 1 | 2 | 1 |
| Carolea | Italy | 4 | 2 | 2 | 2 |
| Carrasquenho de Elvas | Portugal | 2 | 1 | 2 | 2 |
| Cerezuela | Spain | 2 | 1 | 2 | 1 |
| Chalchali | Syria | 4 |  | 4 | 1-2 |
| Chetoui | Tunisia | 3 | 3 | 1 | 1-2 |
| Cirujal | Spain | 10 | 6 | 5 | 1-3 |
| Confetto | Italy | 5 | 5 |  | 1-3 |
| Corbella-817 | Spain | 2 |  | 2 | 1 |
| Cordovil de Serpa | Portugal | 2 | 1 | 2 | 1 |
| Dolce Agogia | Italy | 2 |  | 2 | 2 |
| Frantoio | Italy | 9 | 5 | 5 | 1-3 |
| Gemlik | Turkey | 2 | 2 | 2 | 1 |
| Gerboui | Tunisia | 2 | 1 | 2 | 1 |
| Giarfara | Italy | 2 | 2 |  | 1 |
| Giarraffa | Italy | 3 | 3 |  | 2-4 |
| Gordal de Granada | Spain | 3 | 2 | 3 | 1-2 |
| Gordal de Hellín | Spain | 2 |  | 2 | 3 |
| Gordal Sevillana | Spain | 6 | 3 | 4 | 1-4 |
| Grappolo | Italy | 2 | 2 | 2 | 2 |
| Hojiblanca | Spain | 3 | 1 | 3 | 1-2 |
| Idleb | Syria | 2 | 2 |  | 1 |
| Itrana | Italy | 2 | 2 | 1 | 1 |
| Jlot | Syria | 5 | 4 | 1 | 1-4 |
| Kalokerida | Greece | 2 | 2 | 1 | 2 |
| Karamani | Syria | 3 | 2 | 1 | 2 |
| Kato Drys | Cyprus | 6 | 4 | 2 | 1-3 |
| Khnfse | Syria | 2 | 2 |  | 1 |
| Leccino | Italy | 3 | 2 | 2 | 1-3 |
| Lechín de Sevilla | Spain | 2 | 1 | 2 | 2 |
| Maiatica di Ferrandina | Italy | 2 | 2 |  | 1 |
| Manzanilla Cacereña | Spain | 3 | 1 | 3 | 1-3 |
| Manzanilla de Agua | Spain | 2 | 1 | 2 | 3 |
| Manzanilla de Sevilla | Spain | 7 | 3 | 6 | 1-4 |
| Masabi | Syria | 2 | 1 | 1 | 2 |
| Mastoidis | Greece | 2 | 1 | 1 | 3 |
| Maurino | Italy | 2 | 2 | 1 | 3 |
| Mawi | Syria | 3 | 1 | 2 | 1 |
| Mollar de Cieza | Spain | 4 | 2 | 4 | 2 |
| Moraiolo | Italy | 7 | 6 | 2 | 1-3 |
| Morchiaio | Italy | 6 | 6 |  | 1-3 |
| Moresca | Italy | 3 | 3 |  | 2 |
| Morisca | Spain | 3 | 2 | 1 | 1-2 |
| Morrut | Spain | 2 | 2 | 2 | 1 |
| Negrillo de Arjona | Spain | 2 | 1 | 2 | 1 |
| Nocellara del Belice | Italy | 4 | 4 |  | 1-3 |
| Ocal | Spain | 5 | 3 | 4 | 1-4 |
| Ogliarola del Vulture | Italy | 2 | 2 |  | 1 |
| Passulunara | Italy | 2 | 2 |  | 1 |
| Pavo | Spain | 2 |  | 2 | 2 |
| Pequeña de Casas Ibañez^1^ | Spain | 2 |  | 2 | 1 |
| Picholine | France | 2 | 1 | 2 | 1 |
| Picholine Marocaine | Morocco | 3 | 2 | 3 | 1-2 |
| Picual | Spain | 3 | 2 | 3 | 1-2 |
| Picudo | Spain | 2 | 1 | 2 | 2 |
| Ravece | Italy | 2 | 2 |  | 1 |
| Sant Agostino | Italy | 2 | 2 | 1 | 2 |
| Uovo di Piccione | Italy | 2 | 1 | 2 | 1 |
| Valanolia | Greece | 3 |  | 3 | 2-4 |
| Varudo | Spain | 3 | 1 | 3 | 2-4 |
| Verdale | France | 2 | 1 | 2 | 2 |
| Verdial de Badajoz | Spain | 4 | 1 | 4 | 1-4 |
| Verdial de Huévar | Spain | 2 | 1 | 2 | 1 |
| Zaity | Syria | 2 | 2 | 1 | 1 |
| **Total (74)** |  | **228** | **142 (13^1^)** | **136 (9^2^)** |  |

^1^13 varieties showed variants only in WOGBM (i.e. Atounsi Setif ; Confetto ; Idleb ; Maiatica di Ferrandina ; Giarfara; Giarraffa ; Ogliarola del Vulture ; Passulunara ; Ravece ; Khnfse ; Moresca ; Nocellara del Belice ; Morchiaio).

^2^9 varieties showed variants only in WOGBC (i.e. Alfafara ; Belluti ; Chalchali; Corbella-817 ; Dolce Agogia ; Gordal de Hellín ; Pavo ; Pequeña de Casas Ibañez; Valanolia).
