# Supplementary material for: Characterization of Worldwide Olive Germplasm Banks of Marrakech (Morocco) and Córdoba (Spain): Towards management and use of olive germplasm in breeding programs
Source: PLoS One. 2019 Oct 17;14(10):e0223716. doi: 10.1371/journal.pone.0223716 (PMC6797134; doi:10.1371/journal.pone.0223716)
Supplement: S7 Table — (DOCX) [file pone.0223716.s007.docx]

**S7 Table.** Cases of synonyms found in the identification process in Marrakech and Cordoba collections.

| **Cultivar (origin)** | **Synonyms (origin)** | **References** |
| --- | --- | --- |
| Abbadi^1^ (Syr) | Abou Akfa^2^ (Syr) |  |
| Aaleth^2^ (Alg) | Abani^2^ (Alg) |  |
| Aguenaou^2^ (Alg) | Grosse du Hamma^2^ (Alg) |  |
| Alfafara^1^ (Sp) | Royal de Villena^1^ (Sp) | [1] |
| Americano^2^ (It) | Americano Itafir^2^ (It) and Firenzuolo^2^ (It) | Firenzuolo [2] |
| Atounsi Setif^2^ (Alg) | Aghchren de Titest^2^ (Alg) |  |
| Ayvalik^1^ (Tur) | Edremit YagliK^1^ (Tur) | [3] |
| Azapa^1^ (Arg) | Arauco^1^ (Chl) | [3] |
| Azeradj^2^ (Alg) | Bouchouk Soummam^2^ (Alg) and Aberkane^2^ (Alg) |  |
| Azeradj Tamokra^2^ (Alb) | Aghchren d'el Ousseur^2^ (Alg) |  |
| Beladi^2^ (Lbn) | Agii Trymithias^2^ (Cyp), Anafotia^2^ (Cyp), Analiontas^2^ (Cyp), Athalassa^2^ (Cyp), Arediou^2^ (Cyp), Bakuri^2^ (Syr), Lagoudera^2^ (Cyp), Lefkara^2^ (Cyp), Lefkosia^2^ (Cyp), Lythrodontas^2^ (Cyp), Mazotos^2^ (Cyp), Paliometocho^2^ (Cyp), Roumani Kana^2^ (LBN) | Analiontas/Paliometocho and lefkara/ Lythrodontas [4] |
| Belluti^1^ (Tur) | Yun Gelebi^1^ (Tur) | [1] |
| Bosana^1^ (It) | Peranzana^1^ (It), Palma^2^ (It), Olieddu^2^ (It) and Sassarese^2^ (It) | Peranzana and Sassarese [5] and Palma [6] |
| Buga^12^ (HRV) | Plominka Bjelica^2^ (HRV) | [7] |
| Callosina^1^ (Sp) | Dahbia^2^ (Mor) |  |
| Cerezuela^1^ (Sp) | Morejona^12^ (Sp) | [1] |
| Changlot Real^12^ (Sp) | Torcio de Cabra^1^ (Sp) | [1] |
| Chetoui^12^ (Tun) | Blanquette de gastu^2^ (Alg), Blanquette de Guelma^2^ (Alg), Bouchoukra^2^ (Alg) and Chaîbi Antha^2^ (Tun) | Blanquette de gastu and Blanquette de Guelma [8] |
| Cirujal^1^ (Sp) | Throumbolia^2^ (Gre), Gremignolo di Bolgheri^2^ (It), Grossolana^2^ (It) |  |
| Confetto^2^ (It) | Tonda Di Cagliari^2^ (It), Maiorca^2^ (It), Manna^2^ (It), Strogylolia^2^ (Gre), Sivigliana da Mensa^2^ (It) and Nera di Gonnos^2^ (It) | Tonda Di Cagliari and Maiorca [9], Nera di Gonnos by [10] and Manna by [11,19] |
| Cordovil de Serpa^12^ (Por) | Madural^1^ (Por) | [12] |
| Craputea^2^ (It) | Leccione^2^ (It) |  |
| Dhokar^2^ (Tun) | Dhokar Tataouine^2^ (Tun) |  |
| Dressi^2^ (Tun) | Deras^2^ (Tun) and R'khami^2^ (Tun) |  |
| Ferkani^2^ (Alg) | Jemri bouchouka^2^ (Tun) and Mekki^2^ (Alg) |  |
| Frantoio^12^ (It) | Frantoio A. Corsini^1^ (It), Oblonga^1^ (It), Maelia^1^ (Isr), Razzola^1^ (It), Augellina^2^ (It), Arancino^2^ (It), Cailletier^2^ (Fr), Correggiolo di pallesse^2^ (It), Corsicana da olio^2^ (It), Larcianese^2^ (It), Puntino^2^ (It), Razzo^2^ (It) and San Lazzaro^2^ (It) | Frantoio A. Corsini, Oblonga, Maelia, and Razzola [1,13], Corsicana da olio [10], Razzo [14], Larcianese [2] and Correggiolo di pallesse [15] |
| Gemlik^1^ (Tur) | Samsun Tuzlamalik^1^ (Tur), Kfar Zita^2^ (Syr) | [1] |
| Gerboui^1^ (Tun) | Marsaline^12^ (Tun) | [1] |
| Giarraffa^2^ (It) | Pizzo di corvo^2^ (It) | [16,17] |
| Gordal de Granada^1^ (Sp) | Manzanilla del Centro^12^ (Sp), Manzanilla de Jaén^1^ (Sp), Gordalejo^12^ (Sp) and Nabali^1^ (Isr) | [1] |
| Gordal Sevillana^12^ (Sp) | Santa Caterina^12^ (It) | [12, 18] |
| Grappolo^12^ (It) | Leccio di Corno^12^ (It) and Marzio^2^ (It) | Leccio di Corno [18] |
| Hamra^2^ (Alg) | Boukaïla^2^ (ALG) and Bouricha^2^ (Alg) |  |
| Idleb^2^ (Syr) | Khello^2^ (Syr), Killin^2^ (Syr) and Djbali kini^2^ (Syr) |  |
| Itrana^12^ (It) | Corsicana da mensa^2^ (It), Nera di Oliena^2^ (It), Terza grande^2^ (It), Terza piccola^2^ (It), Tonda di Villacidro^2^ (It) and Paschixedda^2^ (It) | Corsicana da mensa, Nera di Oliena and Tonda di Villacidro [10], Terza piccolo [15,19] and Paschixedda [15] |
| Jlot^2^ (Syr) | Faruke^2^ (Syr), Djlot shami^2^ (Syr), Shami Modabl^2^ (Syr), Korakou^2^ (Cyp) |  |
| Kaissy^2^ (Syr) | Alkkei^2^ (Syr) |  |
| Kalokerida^12^ (Gre) | Olivière^2^ (Fr) |  |
| Kato Drys^12^ (Cyp) | Klirou^1^ (Cyp), Flasou^2^ (Cyp), Kiti^2^ (Cyp), Evrychou^2^ (Cyp), Meniko^2^ (Cyp) and Peristerona^2^ (Cyp) | Klirou [1], Flasou and evryshou [4] |
| Leccino^12^ (It) | Gremignolo^2^ (It) |  |
| Lechín de Granada^12^ (Sp) | Dafnelia^2^ (Gre) |  |
| Lumbardeska^2^ (HRV) | Samo Nova Vas^2^ (HRV) |  |
| Maiatica di Ferrandina^2^ (It) | Žabarka^2^ (HRV) and Uljarica^2^ (HRV) |  |
| Manzanilla Cacereña^12^ (Sp) | Azeitera^12^ (Por) and Negrinha^12^ (Sp, Por) | [3, 18] |
| Manzanilla de Sevilla^12^ (Sp) | Chesna^12^ (Sp) and Redondil^1^ (Sp, Por) | [1, 18] |
| Maurino^12^ (It) | Ginestrino^2^ (It) and Boise^2^ (Slv) | Ginestrino [15] |
| Menya^1^ (Sp) | Manzanilla Picua^1^ (Sp) | [1] |
| Mignolo Cerretano^2^ (It) | Gremigna Tonde^2^ (It) |  |
| Mixani^1^ (Alb) | Ulliri i Bardhe Berat^1^ (Alb) | [1] |
| Mollar de Cieza^12^ (Sp) | Meloncillo^12^ (Sp), Ojúa^1^ (Sp) and Verdalón^1^ (Sp) | [1] |
| Moraiolo^12^ (It) | Carboncella^1^ (It), Moraiolo T. Corsini^1^ (It), Morcaio^2^ (It), Filare^2^ (It), Tondello^2^ (It), Alethriko^2^ (Cyp) | Carboncella [3] and Moraiolo T. Corsini [1] |
| Morchiaio^2^ (It) | Mezanica^2^ (HRV), Sitnica^2^ (HRV), Giogolino^2^ (It), Drobnica^2^ (HRV and Slv) | Sitnica and Drobnica [20] |
| Moresca^2^ (It) | Nerba Catanese^2^ (It) and Olivo di Castiglione^2^ (It) | Nerba Catanese [21] and Olivo di Castiglione [15] |
| Morisca^12^ (Sp) | Santulhana^2^ (Por) |  |
| Morona^1^ (Sp) | Llorón de Ronda^1^ (Sp) | [1] |
| Nocellara del Belice^2^ (It) | Nocellara Messinese^2^ (It) and Misnica^2^ (Slv) |  |
| Ocal^12^ (Sp) | Gordal de Archidona^1^ (Sp) and Verdal de Alhama^12^ (Sp) | [1] |
| Olivastra di Montalcino^2^ (It) | Olivastra Seggianese^2^ (It) and Oleaster^2^ (HRV) | Olivastra Seggianese [22] |
| Passulunara^2^ (It) | Castriciana^2^ (It) |  |
| Picholine Marocaine^12^ (Mor) | Alameño de Marchena^12^ (Sp), Mision de San Vicente^1^ (Mex), Mission Nieland^1^ (USA), Sigoise^12^ (DAZ), Haouzia^12^ (Mor), Menara^12^ (Mor), Cañivano Blanco^1^ (Sp), Aghenfas^2^ (ALG), Hamrani^2^ (Mor), Limli^2^ (ALG), Sinawy^2^ (Egy), Zitoune DK^2^ (Mor), Zitoune Kellal^2^ (Mor) | Alameño de Marchena, Mision de San Vicente, Mission Nieland [3,12,18], Sigoise [23], Haouzia and Menara [24,25], and Zitoune [26] |
| Picual^12^ (Sp) | Olivo Macho de Santisteban Pto^12^ (Sp), Picual de Hoja Oscura^1^ (Sp), Picual de Hoja Clara^12^ (Sp) | [1,27] |
| Picudo^12^ (Sp) | Picudo de Fruto Rojo^1^ (Sp) | [27] |
| Plementa Bjelica^1^ (HRV) | Bianchera^2^ (It) | [7,28] |
| Ravece^2^ (It) | Rotondella^2^ (It) |  |
| Rechino^12^ (Sp) | Palomillo^1^ (Sp) | [1] |
| Remmani ^2^ (Syr) | Dermlali^2^ (Syr) |  |
| Ronde de Miliana^2^ (Alg) | Longue de Miliana^2^ (Alg) |  |
| Rossellino^2^ (It) | Ciliegino^2^ (It), Pesciatino^2^ (It) and Rosino^2^ (It) | Rosino [29] |
| Sant Agostino^12^ (It) | Amphisis^2^ (Gre) |  |
| Tabelout^2^ (Alg) | Takesrit^2^ (Alg) |  |
| Uovo di Piccione^12^ (It) | Novo^1^ (Isr) | [1] |
| Valanolia^1^ (Gre) | Çakir^1^ (Tur) | [3,18] |
| Varudo^1^ (Sp) | Picudo de Montoro^1^ (Sp) | [1] |
| Verdial de Badajoz^1^ (Sp) | Llorón de Iznalloz^1^ (Sp) and Corneja^1^ (Sp) | [1] |
| Verdial de Huévar^2^ (Sp) | Verdial Alentejana^2^ (Por) | [30,31] |
| Villalonga^1^ (Sp) | Branquita de Elvas^1^ (Por) | [3] |
| Zael Al Muhra^2^ (Syr) | Munkar Kak^2^ (Syr) |  |
| Zaity^12^ (Syr) | Khuokhe^2^ (Syr) |  |
| **Total (78)** | **Total (175)** |  |

^1^ Cultivars observed in WOGBC.

^2^ Cultivars observed in WOGBM.

**References**

1. Trujillo I, Ojeda MA, Urdiroz NM, Potter D, Barranco D, Rallo L, et al. Identification of the Worldwide Olive Germplasm Bank of Córdoba (Spain) using SSR and morphological markers. Tree Genet Genomes. 2014;10(1): 141-155.
2. Cantini C, Cimato A, Autino A, Redi A, Cresti. Assessment of the Tuscan olive germplasm by microsatellite markers reveals genetic identities and different discrimination capacity among and within cultivars. J Amer Soc Hort Sci. 2008;133(4): 598-604.
3. Barranco D, Cimato A, Fiorino P, Rallo L, Touzani A, Castañeda C, et al. World olive catalogue of olive varieties. International Olive Oil Council, Madrid, Spain; 2000.
4. Anestiadou K, Nikoloudakis N, Hagidimitriou M, Katsiotis A. Monumental olive trees of Cyprus contributed to the establishment of the contemporary olive germplasm. Plos One. 2017;12(11): e0187697.
5. Erre P, Chessa I, Munoz-Diez C, Belaj A, Rallo L, Trujillo I. Genetic diversity and relationships between wild and cultivated olives (*Olea europaea* L.) in Sardinia as assessed by SSR markers. Genet Resour Crop Evol. 2010;57: 41–54.
6. Chessa I, Erre P, Nieddu M, Nieddu G. Microsatellites characterization of Sardinia olive genetic resources. Olivebioteq. 2006;1: 147-150.
7. Poljuha D, Sladonja B, Seti E, Miloti A, Bandelj D, Jakse J, et al. DNA fingerprinting of olive varieties in Istria (Croatia) by microsatellite marker. Scientia Hortic. 2008;115(3): 223-230.
8. Cimato A and Attilio C. Conservation, characterization, collection and utilization of the genetic resources in olive. Projet CFC/IOC/03; 2003. pp 62.
9. Angiolillo A, Baldoni L, Bandino G, Mulas M. Analisi molecolare con marcatori AFLP delle risorse genetiche di olivo della Sardegna. 4° Convegno Nazionale Biodiversità, germoplasma locale e sua valorizzazione; 2000: 413-416.
10. Baldoni L. Analisi molecolare delle cultivar di olivo italiane. Tornata di Studio nel Lazio, Viterbo 2002; 2004: 81-96.
11. Milella A. L'olivo in Sardegna. L’Italia Agricola; 1965;102: 515-525.
12. Trujillo I, Rallo L, Arus P. Identifying olive cultivars by isozyme analysis. J Am Soc Hortic Sci. 1995;120: 318–324.
13. Barranco D, Trujillo I, Rallo L. Are ‘Oblonga’ and ‘Frantoio’ the same cultivar? HortScience. 2000;35: 1323-1325.
14. Bracci F. Le varietà d’olivo coltivate in Toscana. Le varietà di olivo coltivate in Italia; 1937: 3-16.
15. Muzzalupo I, Vendramin GG, Chiappetta A. Genetic Biodiversity of Italian Olives (*Olea europaea*) Germplasm Analyzed by SSR Markers. Hindawi, Scientific World J. 2014. Article ID 296590, pp 12.
16. Perri E, Lombardo N, Palopoli A, Miele D. Indagine sul germoplasma di olivo della Sicilia mediante marcatori RAPD. 5° Convegno Nazionale Biodiversità ; 1999: 299-304.
17. Fodale AS, Mulé R, Muzzalupo I, Pellegrino M, Perri E (2006) Caratterizzazione del germoplasma di olivo della Sicilia mediante marcatori RAPD. Italus Hortus. 2006;13: 239-241.
18. Belaj A, Satovic Z, Rallo L, Trujillo I. Genetic diversity and relationships in olive (*Olea europaea* L.) germplasm collections as determined by randomly amplified polymorphic DNA. Theor Appl Genet. 2002;105: 638–644.
19. Muzzalupo I, Stefanizzi F, Perri E. Evaluation of Olives Cultivated in Southern Italy by Simple Sequence Repeat Markers. HortScience. 2009;44(3): 582-588.
20. Stambuk S, Sutlovic D, Bakaric P, Petricevic S, Andelinovic S. Forensic botany: potential usefulness of microsatellite-based genotyping of Croatian olive (*Olea europaea* L.) in forensic casework. Croat Med J. 2007;48(4): 556-562.
21. Lombarda P, Fontanazza G. I marcatori AFLP nello studio della biodiversità di olivo (*Olea europaea* L.) in Sicilia con particolare riferimento al germoplasma Ennese. Conv. Naz. Germoplasma olivicolo e tipicità dell’olio. 2003; 1: 196-201.
22. Cimato A, Cantini C, Sani G. L’olivo in Toscana: il germoplasma autoctono. 2001; pp 217.
23. Besnard G, Breton C, Baradat P, Khadari B, Bervillé A. Cultivar identification in the olive (*Olea europaea* L.) based on RAPDS. J Am Soc Hortic Sci. 2001;126: 668-675.
24. Charafi J, El Meziane A, Moukhli A, Boulouha B, El Modafar C, Khadari B. Menara gardens: a Moroccan olive germplasm collection identified by a SSR locus-based genetic study. Genet Resour Crop Evol. 2008;55: 893-900.
25. Zine El Aabidine A, Charafi J, Grout C, Doligez A, Santoni S, Moukhli A, et al. Construction of a Genetic Linkage Map for the Olive Based on AFLP and SSR Markers. Crop Science. 2010;50(6): 2291-2302.
26. Khadari B, Charafi J, Moukhli A and Ater M. Substantial genetic diversity in cultivated Moroccan olive despite a single major variety: a paradoxical situation evidenced by the use of SSR loci. Tree Genet Genomes. 2008;4: 213-221.
27. Barranco D, Trujillo I, Rallo L. Elaiografía Hispanica. In: Rallo L, Barranco D, Caballero JM, Del Rio C, Martin A, Tous J, Trujillo I (eds) Variedades de olivo en España. Mundi-Prensa, Madrid; 2005.
28. Sarri V, Baldoni L, Porceddu A, Cultrera NGM, Contento A, Frediani M, et al. Microsatellite markers are powerful tools for discriminating among olive cultivars and assigning them to geographically defined populations. Genome. 2006;49: 1606-1615.
29. Tavanti G. Trattato teorico-pratico completo sull'ulivo 1. 1819; pp 259.
30. Barranco D, Fernandez-Escobar R, Rallo L. El cultivo del olivo. 1997; pp 605.
31. Belaj A, Trujillo I, De la Rosa R, Rallo L. Polymorphism and Discrimination capacity of randomly amplified polymorphic markers in an olive germplasm bank. J Amer Soc Hort Sci. 2001;126(1): 64-71.
