# Supplementary material for: Characterization of Worldwide Olive Germplasm Banks of Marrakech (Morocco) and Córdoba (Spain): Towards management and use of olive germplasm in breeding programs
Source: PLoS One. 2019 Oct 17;14(10):e0223716. doi: 10.1371/journal.pone.0223716 (PMC6797134; doi:10.1371/journal.pone.0223716)
Supplement: S8 Table — (DOCX) [file pone.0223716.s008.docx]

**S8 Table.** Cases of homonyms found in the identification process in Marrakech and Cordoba collections.

| **Cultivar (Origin)** | **Homonym** |
| --- | --- |
| Abbadi Shalal^12^, Abbadi Abou Gabra-842^1^, Abbadi Abou Gabra-1033^1^ (Bent al Kadi)^a^, Abbadi Abou Gabra-610^2^, Abbadi Helo^2^ | Abbadi |
| Abou Choki-1115^1^, Abou Choki-1126^1^, Abou Kanani^1^, Abou Monkar^2^, Abou Akfa^2^ (Abbadi)^a^, Abou Anaked^2^, Abou Shawka Tadmori^2^ (Abbadi)^a^ | Abou |
| Aggezi Akse^2^, Aggezi Oshime^2^ and Aggezi Shami^2^ | Aggezi |
| Aghchren de Titest^2^ (Atounsi Setif) ^a^ and Aghchren d'el Ousseur^2^ (Azeradj Tamokra)^a^ | Aghchren |
| Alameño Blanco^12^, Alameño de Cabra^1^, Alameño de Marchena^12^ (Picholine Marocaine)^a^, Alameño de Montilla^12^ | Alameño |
| Azeradj^2^ and Azeradj Tamokra^2^ | Azeradj |
| Berri Meslal-397^2^ and Berri Meslal-532^2^ | Berri Meslal |
| Bouchouk Laghlid and Rkik^2^(Ocal)^a^, Bouchouk Lafayette^2^, Bouchouk Soummam^2^ (Azeradj)^a^ | Bouchouk |
| Cañivano Negro^12^ and Cañivano Blanco^1^ (PicholineMarocaine)^a^ | Cañivano |
| Carrasqueño de Elvas^12^, Carrasqueño de Alcaudete^1^, Carrasqueño de Jumilla^12^, Carrasqueño de la Sierra^1^ and Carrasqueño de Porcuna^1^ | Carrasqueño |
| Cordovil de Castelo Branço^1^ and Cordovil de Serpa^12^ | Cordovil |
| Cornicabra Murciana^1^ (Callosina)^a^, Cornicabra^12^, Cornicabra de Jerez Caballeros^1^ and Cornicabra de Mérida^1^ | Cornicabra |
| Corsicana da mensa^2^ (Itrana)^a^ and Corsicana da olio^2^ (Frantoio)^a^ | Corsicana |
| Crnica^1^ and Crnica-399^2^ | Crnica |
| Djlot Shami^2^ (Jlot)^a^ and Djlot Tadmori^2^ | Djlot |
| Dolce^1^, Dolce Agogia^1^ and Dolce di Rossano^2^ | Dolce |
| Dulzal^12^ and Dulzal de Carmona^1^ | Dulzal |
| Escarabajuelo de Atarfe^1^, Escarabajuelo de Posadas^12^ and Escarabajuelo de Úbeda^12^ | Escarabajuelo |
| Gordal de Granada^1^, Gordal de Hellín^1^, Gordal de Vélez Rubio^1^, Gordal Sevillana^12^ and Gordal de Archidona^1^ (Ocal)^a^ | Gordal |
| Gremignolo di Bolgheri^2^ (Cirujal)^a^ and Gremignolo^2^ (Leccino)^a^ | Gremignolo |
| Grosal de Cieza^1^ and Grosal Vimbodí^1^ | Grosal |
| Habichuelero de Baena^1^ and Habichuelero de Grazalema^12^ | Habichuelero |
| Jlot^1^ and Jlot-841^1^ | Jlot |
| Lazzero^2^ and Lazzero di prata^2^ | Lazzero |
| Leccio del Corno^12^ (Grappolo) and Leccio Maremmano^2^ | Leccio |
| Lechín de Granada^12^ and Lechín de Sevilla^12^ | Lechín |
| Lentisca^1^, Lentisca-244^2^ and Lentisca-206^2^ | Lentisca |
| Llorón de Atarfe^12^, Llorón de Iznalloz^12^ (Verdial de Badajoza)^a^ and Llorón de Ronda^12^ (Morona)^a^ | Llorón |
| Mahati-846^1^, Mahati-1010^1^ and Mahati-615^2^ | Mahati |
| Majhol-152^1^, Majhol-1059^1^, Majhol-1063^1^ and Majhol-1122^1^ | Majhol |
| Manzanilla de Jaén^1^ (Gordal de Granada)^a^, Manzanilla Cacereña^12^, Manzanilla de Abla^12^, Manzanilla de Agua^12^, Manzanilla de Almería^1^, Manzanilla de Hellín^12^, Manzanilla de Montefrío^12^, Manzanilla de Sevilla^12^, Manzanilla del Piquito^1^, Manzanilla del Centro^12^ (Gordal de Granada)^a^, Manzanilla Picua^1^ (Menya)^a^, Manzanilla de San Vicente^1^, Manzanilla Prieta^1^ | Manzanilla |
| Mignolo^1^ and Mignolo Cerretano^1^ | Mignolo |
| Mission Moojeski^1^ and Mission Nieland^1^ (Picholine Marocaine)^a^ | Mission |
| Mollar Basto^1^ and Mollar de Cieza^12^ | Mollar |
| Morisca^12^ and Morisca de Mancor^1^ | Morisca |
| Neb Jmel-283^2^ and Neb Jmel-452^2^ | Neb Jmel |
| Negrillo de Arjona^12^, Negrillo de Estepa^12^, Negrillo de Iznalloz^12^, Negrillo de la Carlota^1^ and Negrillo Redondo^12^ | Negrillo |
| Nera di Oliena^2^ (Itrana)^a^ and Nera di Gonnos^2^ (Confetto)^a^ | Nera |
| Nerba^2^ and Nerba Catanese^2^ (Moresca) ^a^ | Nerba |
| Nevadillo de Santisteban Pto^1^ and Nevadillo Blanco de Jaén^1^ | Nevadillo |
| Nevado Azul^12^, Nevado Basto^1^ and Nevado Rizado^12^ | Nevado |
| Nocellara del Belice^2^ and Nocellara Etnea^2^ | Nocellara |
| Ogliarola del Bradano^2^ and Ogliarola del Vulture^2^ | Ogliarola |
| Olivastra di Populonia^2^ and Olivastra di Montalcino^2^ | Olivastra |
| Olivo Macho de Santisteban Pto^12^ (Picual)^a^, Olivo de Mancha real^12^, Olivo de Maura^1^, Olivo del Mulino^2^, Olivo di Casavecchia^2^, Olivo di Mandanici^2^, Olivo di San Lorenzo^2^, Olivo di Castiglione^2^ (Moresca)^a^ | Olivo |
| Pico Limón^12^ and Pico Limón de Grazalema^12^ | Pico Limón |
| Picual^12^ and Picual de Almería^1^ | Picual |
| Picudo^12^, Picudo Blanco de Estepa^1^, Picudo de Montoro^1^ (Varudo)^a^ | Picudo |
| Redondilla de Grazalema^1^ and Redondilla de Logroño^1^ | Redondilla |
| Ronde de la Ménara^2^ and Ronde de Miliana^2^ | Ronde |
| Rossellino^2^ and Rossellino Cerretano^2^ | Rossellino |
| Royal de Calatayud^12^, Royal de Cazorla^12^, Royal de Sabiñan^1^ and Royal de Villena^1^ (Alfafara)^a^ | Royal |
| Samo^2^ and Samo Nova Vas^2^ (Lumbardeska)^a^ | Samo |
| Shami^2^ and Shami Modabl^2^ (Jlot)^a^ | Shami |
| Sivigliana da Olio^2^ and Sivigliana da Mensa^2^ (Confetto)^a^ | Sivigliana |
| Toffahi^1^, Toffahi-1000^1^, Toffahi-486^2^ and Toffahi-621^2^ | Toffahi |
| Tonda di Villacidro^2^ (Itrana)^a^, Tonda Iblea^2^, Tonda Di Cagliari^2^ (Confetto)^a^ | Tonda |
| Ulliri i Bardhe Berat^1^ (Mixani)^a^, Ullirii BardheiTiranes^1^ and Ullirii Kuq^1^ | Ulliri |
| Verdal de Alhama^12^ (Ocal)^a^ and Verdal de Manresa^1^ | Verdal |
| Verdial de Badajoz^1^, Verdial de Huévar^1^, Verdial de Cádiz^1^ and Verdial de Vélez-Málaga^1^ and Verdial transmontana^2^ | Verdial |
| **Total (179)** | **Total (60)** |

^1^Cultivars observed in WOGBC, ^2^ Cultivars observed in WOGBM, ^a^Between brackets represents synonymous identified after identification process using both SSR markers and morphological traits.
