# Supplementary material for: Characterization of Worldwide Olive Germplasm Banks of Marrakech (Morocco) and Córdoba (Spain): Towards management and use of olive germplasm in breeding programs
Source: PLoS One. 2019 Oct 17;14(10):e0223716. doi: 10.1371/journal.pone.0223716 (PMC6797134; doi:10.1371/journal.pone.0223716)
Supplement: S10 Table — (DOCX) [file pone.0223716.s010.docx]

**S10 Table.** SSR markers involved in the description of molecular variants (MV) in the two collections, their repetitive motifs and alleles showing variation compared to reference alleles.

| **Loci** | **Repeat motif** | **MV** | **Variant alleles (reference alleles-reference cultivar)** |
| --- | --- | --- | --- |
| DCA01 | (**GA**)22 | Yes | 204/**252**, 204/**261**, 204/**268**, 204/**270**, 204/**274** (204/272-Frantoio)  204/**261** (204/258-Leccino) |
| DCA03 | (**GA**)19 | Yes | 243/**249** (243/247-Beladi)  **249**/251 (247/251-Gordal Sevillana)  237/**249** (237/247-Picual)  229/**251** (229/253-Sant Agostino)  243/**251** (243/253-Verdial de Badajoz) |
| DCA04 | (**GA**)16 | Yes | 129/**186** (129/184-Bolvino)  **130**/161 (138-161-Buga)  159/**186** (159/182-Dolce Agogia)  **165**/**165** (161/161-Giarraffa)  130/**163** (130/159-Maiatica di Ferrandina)  190/**192** (182/190-Mastoidis)  **186**/194 (184/194-Morrut)  **148**/186 (146/186-Ogliarola del Vulture)  161/**192** (161/188-Passulunara)  129/**190** (129/186-Valanolia) |
| DCA05 | (**GA**)15 | Yes | 199/**203** (199/205-Bosana) |
| DCA08 | (**GA**)18 | Yes | 135/**141** (135/139-Itrana)  **135**/139 (139/139-Manzanilla de Agua)  139/**145** (139/139-Maurino)  **135**/137 (134/137-Mollar de Cieza)  129/**129** (129/135-Uovo di Piccione)  135/**141** (135/139-Verdial de Huévar) |
| DCA09 | (**GA**)23 | Yes | 160/**196** (160/198-Carolea)  **192**/202 (184/202-Cirujal)  180/**204** (180/208-Frantoio)  192/**204** (192/202-Gemlik)  **182**/192 (174/192-Gordal de Granada)  160/**202** (160/206-Jlot)  170/**202** (170/206-Kato Drys)  160/**208** (160/204-Leccino)  160/**198** (160/204-Lechín de Sevilla)  160/**200** (160/204-Manzanilla de Sevilla)  182/**202**, 182/**208**, 182/**210** (182/204-Moraiolo)  160/**208** (160/204-Ocal)  192/**202** (192/204-Picholine Marocaine) |
| DCA10 | (TA)14(**GA**)17 | Yes | **156**/**156** (154/154-Gordal Sevillana)  **152**/**152** (154/154-Manzanilla Cacereña)  **164**/**164** (162/162-Moraiolo)  194/**221** (194/218-Ocal) |
| DCA11 | (**GA**)26(GGGA)4 | Yes | **158**/**178**; 160/**178** (160/180-Alameño de Montilla)  160/**174** (160/178-Atounsi Setif)  **148**/160 (146/160-Beladi)  146/**176** (146/178-Carolea)  **178**/178 (176/178-Confetto)  160/**182**, **164**/**182** (160/178-Gordale Sevillana)  140/**174** (140/178-Mollar de Cieza)  140/**174** (140/178-Morchiaio)  **140**/178 (178/178-Moresca)  176/**176** (176/178-Pavo)  140/**178** (140/182-Picudo) |
| DCA15 | (CA)3G(AC)14 | Yes | 243/**263** (243/243-Alfafara)  **263**/263 (243/263-Gordal de Hellín)  243/**254** (243/243-Grappolo)  **263**/263 (261/263-Mastoidis)  **243**/**243** (263/263-Verdial de Badajoz) |
| DCA16 | (GT)13(**GA**)29 | Yes | 152/**175** (152/173-Alfafara)  122/**179** (122/173-Chetoui)  148/**177** (148/173-Giarfara)  124/**179** (124/177-Idleb)  122/**218**, 122/**222**, 122/**230** (122/220-Jlot)  124/**216**, 124/**218**, 124/**220**, 124/**228** (124/226-Kato Drys)  122/**216** (122/224-Mawi)  152/**175** (152/173-Morisca)  122/**177** (122/175-Negrillo de Arjona)  152/**177** (152/173-Ocal)  124/**179** (124/177-Zaity) |
| DCA18 | (CA)4CT(CA)3(**GA**)19 | Yes | 172/**172** (172/174-Beladi)  168/**168** (168/172-Callosina)  172/**172** (172/174-Frantoio)  172/**180** (172/176-Gordale Sevillana)  172/**172** (172/174-Kato Drys)  164/**170** (164/172-Lechín de Sevilla)  168/**168** (168/172-Mollar de Cieza)  **176**/180 (172/180-Chalchali) |
| EMO90 | (CA)10 | No |  |
| GAPU59 | (CT)9 | Yes | **206**/210 (210/210-Ocal)  **206**/210 (210/210-Verdale) |
| GAPU71A | (AG)10 | No |  |
| GAPU71B | GA(AG)6(AAG)8 | Yes | **121**/**121** (124/124-Masabi)  121/**130** (121/127-Morchiaio)  **121**/141 (127/141-Pavo) |
| GAPU101 | (**GA**)8G3(AG)3 | Yes | **189**/**219** (191/217-Carrasqueño de Elvas) |
| GAPU103 | (TC)26 | Yes | 147/**174** (147/171-Bolvino)  147/**184** (147/190-Cerezuela)  147/**174**, 147/**178** (147/171-Cirujal)  **171**/184, **171**/**186** (174/184-Confetto)  171/**182** (171/184-Corbella-817)  159/**176** (159/171-Frantoio)  133/**188** (133/184-Giarraffa)  171/**184** (171/186-Grappolo)  147/**184** (147/186-Hojiblanca)  137/**174** (137/171-Jlot)  171/**188** (171/184-Leccino)  147/**188** (147/184-Nocellara del Belice)  157/**190** (157/184-Pequeña de Casas Ibañez)  **147**/188 (149/188-Picholine)  147/**184** (147/171-Ravece)  147/**186** (147/184-Chalchali) |
| UDO011 | (CT)7(CA)10(CT)2(CA)2  CT(CA)2CT(CA)9 | Yes | 127/**131** (127/134-Gordal de Hellín)  119/**131** (119/134-Manzanilla de Sevilla) |
| UDO017 | (TG)11 | No |  |
| UDO043 | (GT)12 | Yes | 170/**216** (170/218-Adkam)  172/**216** (172/214-Atounsi Setif)  175/**218** (175/216-Blanqueta)  175/**185** (175/187-Bosana)  **170**/175 (172/175-Carolea)  175/**214** (175/216-Cirujal)  208/**218** (208/212-Cordovil de Serpa)  175/**208**, 175/**212** (175/214-Frantoio)  175/**212** (175/214-Gerboui)  172/**218** (172/216-Gordal de Granada)  172/**218** (172/216-Gordal de Hellín)  208/**218** (208/216-Hojiblanca)  172/**214** (172/216-Karamani)  172/212 (172/216-Manzanilla Cacereña)  172/218 (172/216-Manzanilla de Agua)  **208**/**208**, 210/**210**, **208**/212, 210/**214** (210/212-Manzanilla de Sevilla)  172/**216** (172/214-Mollar de Cieza)  172/**210** (172/212-Picholine Marocaine)  208/**214** (208/212-Picual)  **162**/212 (172/212-Verdial de Badajoz)  170/**216** (170/218-Chalchali) |
