# Supplementary figures and images for: Characterization of Worldwide Olive Germplasm Banks of Marrakech (Morocco) and Córdoba (Spain): Towards management and use of olive germplasm in breeding programs
Source: PLoS One. 2019 Oct 17;14(10):e0223716. doi: 10.1371/journal.pone.0223716 (PMC6797134; doi:10.1371/journal.pone.0223716)

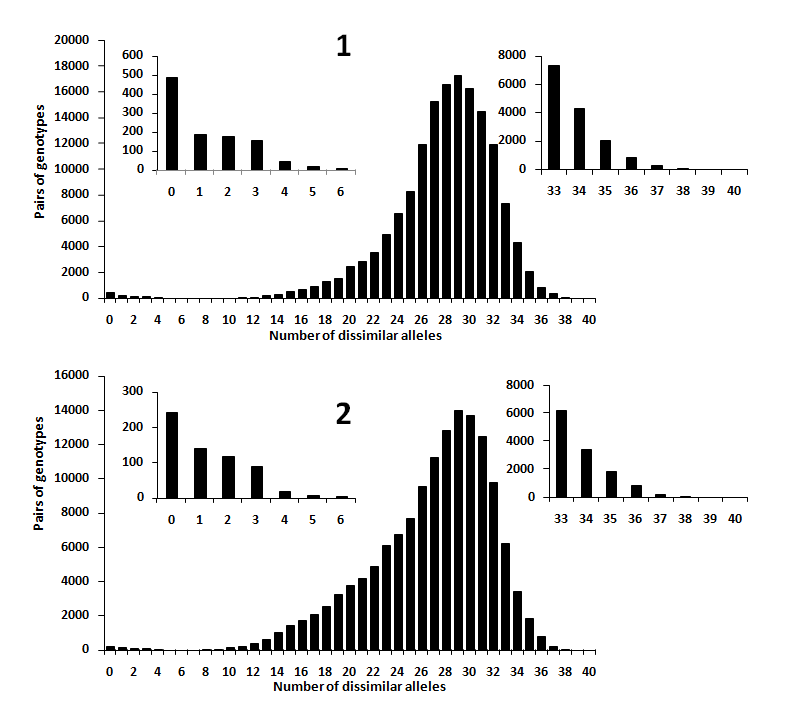

Supplement: S1 Fig — (TIF) [file pone.0223716.s011.tif]

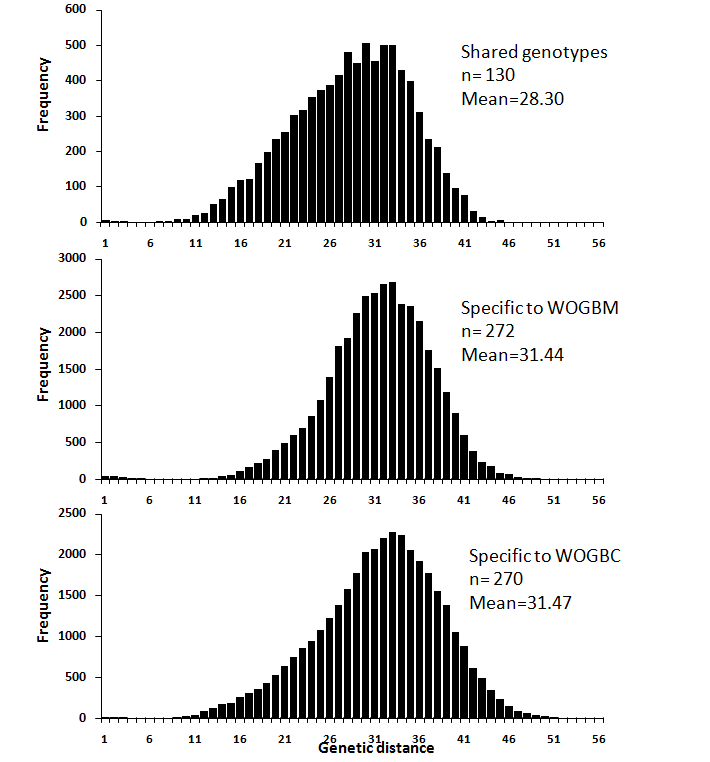

Supplement: S2 Fig — (TIF) [file pone.0223716.s012.tif]

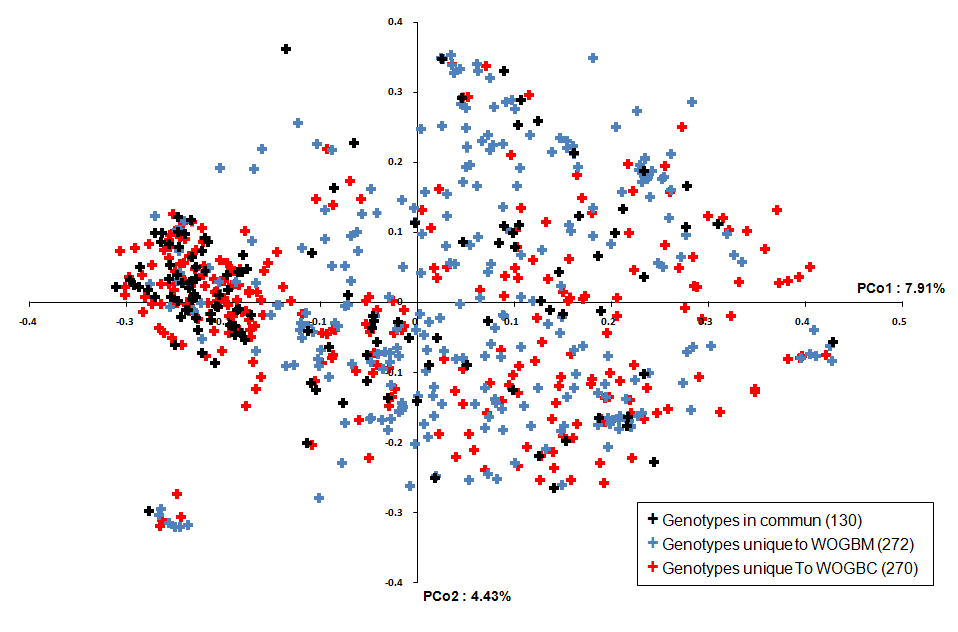

Supplement: S3 Fig — (TIF) [file pone.0223716.s013.tif]

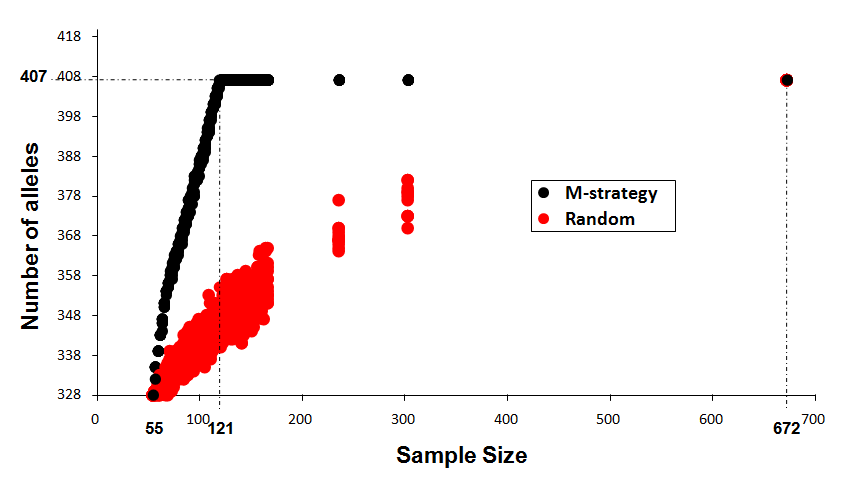

Supplement: S4 Fig — (TIF) [file pone.0223716.s014.tif]
